# Contribution of changes in the orexin system and energy sensors in the brain in depressive disorder - a study in an animal model

**DOI:** 10.1007/s43440-023-00559-0

**Published:** 2024-01-09

**Authors:** Katarzyna Głombik, Magdalena Kukla-Bartoszek, Katarzyna Curzytek, Agnieszka Basta-Kaim, Bogusława Budziszewska

**Affiliations:** Laboratory of Immunoendocrinology, Department of Experimental Neuroendocrinology, Maj Institute of Pharmacology, Polish Academy of Sciences, Smętna 12, 31-343 Kraków, Poland

**Keywords:** Brain, Depression, Dexamethasone, Orexins, Metabolism

## Abstract

**Background:**

Maternal elevated glucocorticoid levels during pregnancy can affect the developing fetus, permanently altering the structure and function of its brain throughout life. Excessive action of these hormones is known to contribute to psychiatric disorders, including depression.

**Materials:**

The study was performed in a rat model of depression based on prenatal administration of dexamethasone (DEX) in late pregnancy (0.1 mg/kg, days 14–21). We evaluated the effects of prenatal DEX treatment on the cognition and bioenergetic signaling pathways in the brain of adult male rats, in the frontal cortex and hippocampus, and in response to stress in adulthood, using behavioral and biochemical test batteries.

**Results:**

We revealed cognitive deficits in rats prenatally treated with DEX. At the molecular level, a decrease in the orexin A and orexin B levels and downregulation of the AMPK-SIRT1-PGC1α transduction pathway in the frontal cortex of these animals were observed. In the hippocampus, a decreased expression of orexin B was found and changes in the MR/GR ratio were demonstrated. Furthermore, an increase in HDAC5 level triggered by the prenatal DEX treatment in both brain structures and a decrease in MeCP2 level in the hippocampus were reported.

**Conclusions:**

Our study demonstrated that prenatal DEX treatment is associated with cognitive dysfunction and alterations in various proteins leading to metabolic changes in the frontal cortex, while in the hippocampus adaptation mechanisms were activated. The presented results imply that different pathophysiological metabolic processes may be involved in depression development, which may be useful in the search for novel therapies.

**Supplementary Information:**

The online version contains supplementary material available at 10.1007/s43440-023-00559-0.

## Introduction

Despite well-known facts about the brain’s high energy demand, an understanding of how disturbances in the processes of energy transformations of the brain affect the functioning of this organ remains unclear. Among many other factors, orexins, neuropeptides with a multidirectional activity spectrum, play an important role in regulating brain energy homeostasis. These are factors synthesized mostly by neurons located in the lateral part of the hypothalamus, but projections of hypothalamic orexin-releasing neurons spread far, reaching many CNS structures. The widespread distribution of orexins and their receptors indicates their pleiotropic actions as neurotransmitters, neuromodulators, and hormones [1]. The role of orexins includes the control of energy metabolism processes, the regulation of tropic hormone release, and the regulation of sleep/wake rhythm. Orexins A and B act via G-protein type 1 and type 2 coupled receptors (OxR1 and OxR2, respectively). Orexins are known to contribute to cognitive function [2] and the stress response [3]. A reduction in orexin system function in animal models of depression was observed in a model of endogenous depression in Wistar-Kyoto rats [4] and in a model of social stress [5]. The expression of orexins is regulated by glucocorticoids, and the activity of orexin neurons rapidly changes in response to stress [6]. Many brain regions of dense orexin localization contribute to depressive behaviors after stress; however, this depends on the duration, intensity, and type of stressor [3]. Additionally, other data link stress-related mental disorders with perturbations in energy metabolism [7], and inappropriate distribution of systemic energy resources in response to stress seems to be a predisposing factor for depression [8].

**Fig. 1:**
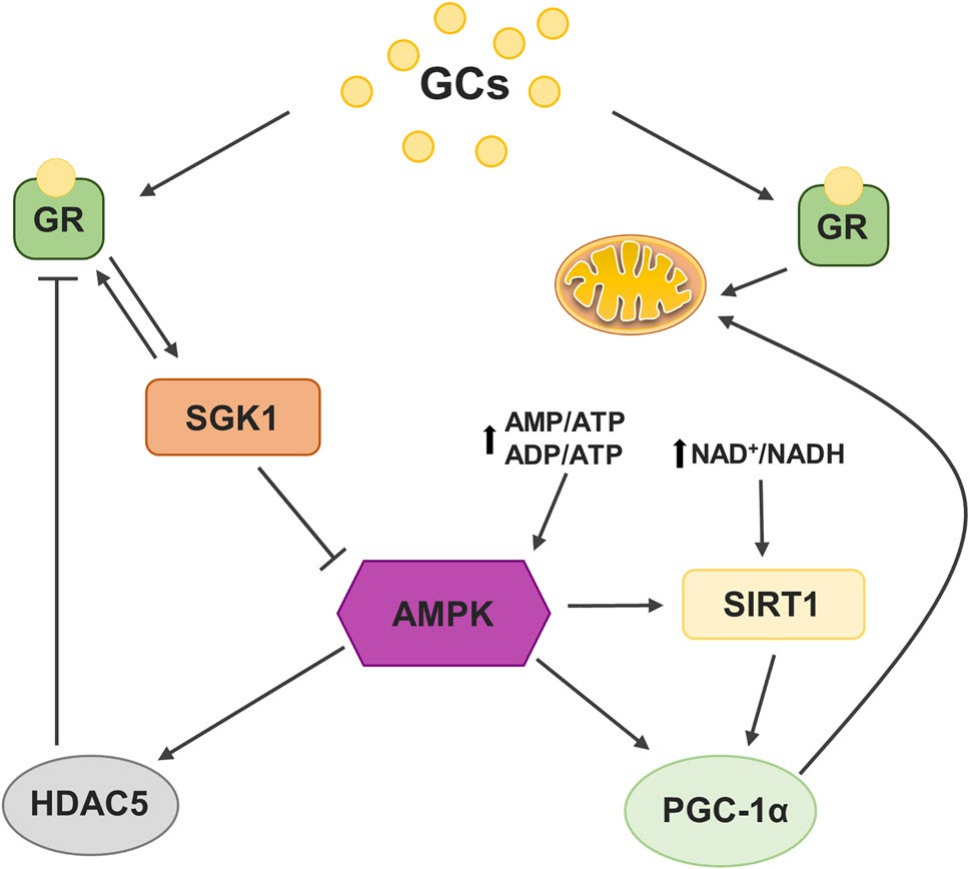
Schematic diagram of the glucocorticoids (GCs) effects on AMPK-SIRT1-PGC1α transduction pathway. GCs inhibit AMPK kinase activation by SGK1 kinase stimulation. Inhibited AMPK is involved in the HDAC5 upregulation, which suppresses the transcription of many genes including NR3C1 encoding GR, by histone deacetylation and inducing tight DNA wrapping. AMPK may be also activated by the changes in AMP/ATP or ADP/ATP ratios, in turn affecting SIRT1 and PGC1α, involved in mitochondrial functioning and biogenesis and thus cellular energy homeostasis

AMP-activated protein kinase (AMPK) is an energy sensor and guardian of mitochondrial homeostasis that plays a key role in cellular energy metabolism; it regulates glucose and lipid metabolism and integrates signaling pathways between the periphery and the hypothalamus to regulate food consumption and whole-body energy expenditure [9]. To meet the needs of the growing energy demand, AMPK can increase the expression of genes related to glucose transport and glycolysis while simultaneously decreasing genes involved in lipid synthesis [10]. Decreased phosphorylation, which leads to the inactivation of AMPK, has been demonstrated to be involved in depression-like behavior development in rodents subjected to chronic stress [11]. AMPK is also an essential element engaged in the maintenance of glucocorticoid receptor (GR) function. Glucocorticoids (GCs) stimulate serum/glucocorticoid-regulated kinase 1 (SGK1) and inhibit AMPK activation. AMPK inhibition promotes the activation of histone deacetylase 5 (HDAC5), leading to a decrease in GR after chronic exposure to GCs, and AMPK stimulation reverses induced by the GCs: depressive-like phenotype and decrease in GR [12]. Moreover, AMPK can also be activated via alterations in the cellular AMP/ATP and/or ADP/ATP ratios, and together with sirtuin 1 (SIRT1) and peroxisome proliferator-activated receptor gamma coactivator 1-alpha (PGC1α), they are involved in the regulation of the AMPK/SIRT1/PGC1α signaling axis [10]. SIRT1 directly links metabolic dysfunction with transcriptional outputs and modulates the function of transcription factors and coregulators, including peroxisome proliferator-activated receptor gamma (PPARγ) [13]. PGC1α, a PRARγ receptor coactivator, regulates the biogenesis of new mitochondria and plays a decisive role in maintaining carbohydrate, lipid, and energy homeostasis in mammalian cells, which is extremely important in the brain due to the high energy requirement of the neurotransmission process [14]. SIRT1 enhances the activity of PGC1α by deacetylation, which results in tissue readiness to be prepared for changing energy conditions [10]. SIRT1-related regulation of PGC1α was considered to be dependent on the activation of AMPK, but the molecular dependency between AMPK and SIRTs is still unclear (Fig. 1).

Diseases of civilization, such as cancer, metabolic disorders, and mood disorders, remain persistently resistant to traditional medical approaches. For this reason, the search for new mechanisms underlying these disorders and an understanding of the exact mechanics of abnormalities in cell and tissue interactions, with each other and with the environment, are substantial. Options for depression treatment are limited, and patients are dealing with poor or unsatisfactory drug responses [15]. Recently, in addition to the classical theories of depression onset, the hypothesis that mitochondrial dysfunction is associated with mood disorders [16] emerged and is supported by studies that have linked mitochondrial dysfunctions with psychiatric disturbances, mainly in mood and cognition processes [17]. Mitochondria play numerous roles in maintaining homeostasis, responding to the energy needs of cells, and contributing to a wide range of physiological and pathophysiological processes in the brain, including neutralization of reactive oxygen species (ROS), neurogenesis, cell maturation, neural plasticity, and neurodegeneration. The importance of proper mitochondrial morphology and function in the regulation of energy demands, neuronal development, adult neurogenesis, and their impact on memory processes have been raised recently [18–20]. Neuronal differentiation depends on mitochondrial dynamics, and these organelles play a significant role in adult neurogenesis and hippocampal-dependent memory formation. Mitochondrial activity can model, regulate, and maintain memory [21]. This is confirmed by the fact that mitochondrial impairments have been widely observed in neurodegenerative disorders, which are associated with a large number of cognitive deficiencies [22].

We aimed to examine molecular, energetic dysfunctions underlying depression induced by prenatal dexamethasone treatment (excessive glucocorticoid action) and to determine possible different responses to acute stress in adulthood. Furthermore, our goal was to determine whether changes in brain metabolism may affect higher brain functions, such as memory processes which are also altered in depression.

To achieve this set of goals, a rat model of depression based on prenatal dexamethasone (DEX) administration was applied. The current investigation was developed based on the findings of our previous study [23] conducted in this model, in which we observed not only behavioral deficits (depression-like behavior measured in the forced swim test and anxiety behavior demonstrated in the elevated plus maze test), but also a reduction in the oxidative phosphorylation process resulting in a decrease in ATP synthesis and a weakening of the Krebs cycle with the accumulation of glycolysis products. Moreover, the impairment of pyruvate dehydrogenase action and lactate transport to neurons and pyruvate transport to the mitochondria in the DEX-based model were demonstrated by us in the frontal cortex. In the hippocampus, protective mechanisms against changes in energy production were revealed [23]. This study is an attempt to search for the molecular mechanisms responsible for energy changes in the brain resulting from prenatal exposure to DEX.

## Materials and methods

### Animals and treatment

All experiments were conducted to minimize animal suffering and to reduce the number of animals used (3R policy). The study was approved by the Local Ethics Committee in Kraków, Poland (permission no. 211/2021 of 01.07.2021).

Sprague ‒ Dawley rats obtained from Charles River Laboratories (Sulzfeld, Germany) were housed under typical conditions, including a 12-h day/night cycle and room temperature of 22 ± 2 °C, with free access to food and water. As described previously [23], after a quarantine, female rats with identified proestrus phase, based on the afternoon vaginal smear examination, were placed in cages with breeding males overnight. Each mating was confirmed by the presence of sperm the next morning. After confirmation of pregnancy, dams (*n* = 12) were randomly divided into groups (control or DEX-treated) and relocated into separate cages. In the experimental group, animals were subjected to subcutaneous injection of the dexamethasone 21-phosphate disodium salt (DEX, Sigma‒Aldrich, Saint Louis, MO, United States, Cat. no.: D1159) at a concentration of 0.13 mg/kg/ml (equal to 0.1 mg/kg dexamethasone) dissolved in 0.9% saline, each day from 14th day of pregnancy until delivery (21st day). Selected treatment time and a dose were chosen to approximate the conditions of dexamethasone use in the clinic in pregnant women. The control female rats received saline (0.9%) during the same period of pregnancy.

**Fig. 2:**
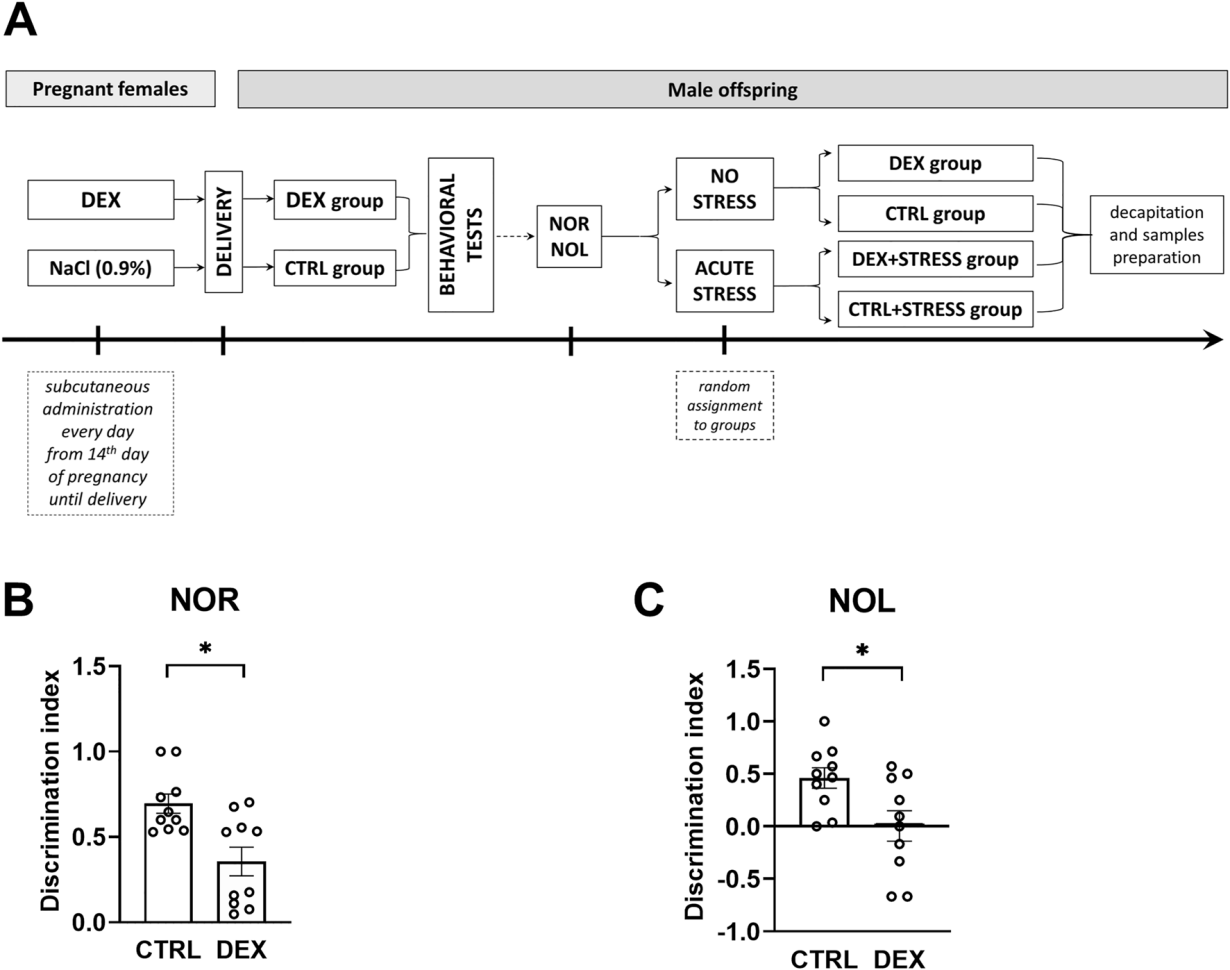
Schematic diagram of the experimental design (**A**). The effect of prenatal dexamethasone treatment on recognition memory was measured by the NOR test (**B**), and the spatial working memory of the animals was measured by the NOL test (**C**). The results are expressed as the mean ± SEM. Statistics: Student’s *t*-test; *n* = 10 for each test; *p < 0.05

Male offspring (DEX *n* = 30, CTRL *n* = 30) were kept with mothers until weaning (3 weeks). Animals born from different litters but in the same DEX or control group were mixed and housed in groups of three to five per cage until 10 weeks of age. Behavioral studies, including the novel object recognition (NOR) and location (NOL) tests, were conducted at the age of 10 weeks. At the final stage of the experiment, subgroups of test and control groups were put into tight plastic cages for one hour of acute immobilization stress and then were returned to their home cages for another hour. Finally, under nonstressed conditions, they were sacrificed by rapid decapitation (between 9 a.m. and 12 p.m.). A schematic overview of the experiment is presented in Fig. 2A.

### Behavioral tests: Novel object recognition and location (NOR and NOL) test

For the estimation of cognition processes, the novel object recognition (NOR) test and the novel object localization (NOL) test were used [24]. In the first phase of the test, each animal was exposed to the apparatus without any object. After 24 h, animals were placed for 3 min in the experimental box with two identical objects placed diagonally. After 1 h, the animals were returned to the container for 3 min, where one object (A) was changed to a different object (B) (NOR test) or the place of one object was changed (NOL test). During this phase, the time spent by every animal exploring each object was measured. In both tests, the discrimination index (DI) based on exploration time (E) of two objects was calculated according to the formula: DI = (EB − EA)/(EA + EB).

### Tissues and blood preparation

After decapitation, trunk blood samples were collected into tubes containing EDTA (Kabe Labortechnik, Nümbrecht, Germany) and centrifuged at 3,000 rpm for 20 min at 4 °C to separate the plasma fraction, which was then transferred into new tubes, frozen, and stored at − 20 °C for further analyses. Moreover, the rat brains were isolated, and the desired structures (frontal cortices and hippocampi) were promptly subjected to dissection on ice-cold glass plates. Isolated brain tissue was collected into tubes, frozen, and stored at − 80 °C until use.

### Whole tissue homogenate sample preparation

The whole tissue homogenate samples were prepared according to the TissueLyser Handbook with slight modifications. The tissues were submerged in PBS or 2% SDS buffers enriched in Halt™ Protease and Phosphatase Inhibitor Cocktail (Thermo Scientific™ Waltham, MA, United States, Cat. no.: 78440) when subjected to ELISA or Western blotting analysis, respectively, and homogenized in 2-mL tubes with stainless steel bead in each using Tissue Lyser II (Qiagen Inc., Valencia, CA, USA) for 5 min at 30 Hz. In the next step, the samples were shaken for 20 min on ice and centrifuged at 14,000 rpm for 20 min at 4 °C. Total protein concentration was assessed in the collected supernatants using the Pierce™ BCA Protein Assay Kit (Thermo Fisher Scientific, Waltham, MA, United States, Cat. no.: 23227). The samples were frozen and stored at − 80 °C until use.

### Nuclear fraction isolation

 The levels of selected nuclear proteins (i.e., HDAC1, HDAC2, HDAC4, HDAC5, MeCP2, Dnmt1, Dnmt3a, and Dnmt3b) were assessed specifically in the nuclear fraction. The fraction was isolated from the investigated brain tissues—frontal cortices and hippocampi using a Nuclear Extraction Kit (Abcam, Cambridge, UK, Cat. no.: ab113474) according to the manufacturer’s protocol. Briefly, the brain structures were prepared for extraction by homogenization in ice-cold homogenization buffer enriched with DTT and protease inhibitor cocktail (Protease Inhibitor Cocktail, Sigma-Aldrich, Saint Louis, MO, United States, Cat. no.: P8340) (1:1000) using a Teflon-glass homogenizer, incubated for 15 min on ice and centrifuged for 10 min at 12,000 rpm at 4 °C. After that, the extraction buffer, also containing DTT and protease inhibitor cocktail, was added to the pellets, and samples were incubated on ice for 15 min with vortexing every 3 min. The suspensions were centrifuged for 10 min at 14,000 rpm at 4 °C, and the supernatants were transferred into new tubes. The protein concentration in the extracted nuclear fractions was assessed using Bradford Reagent (Abcam, Cambridge, UK, Cat. no.: ab119216) according to the manufacturer’s protocol.

### Western blotting assay

Before the Western blotting analysis, the protein concentration in each sample was normalized to 3 μg/µl. Then, 20 μg of total protein was mixed with 4 × Laemmli Sample Buffer (Bio-Rad, Hercules, CA, USA, Cat. no.: 1610747) and boiled for 5 min at 95 °C. Criterion™ TGX™ Precast Midi Protein Gels (Bio-Rad, Hercules, CA, USA, Cat. no.: 5671095) were used for protein separation, which was conducted under a constant voltage of 150 V for 1 h, followed by 30 min of semidry transfer to PVDF membranes (Sigma-Aldrich, Saint Louis, MO, USA, Cat. no.: 3010040001). To perform incubation with various antibodies at the same time, membranes were cut into smaller pieces and then blocked with 5% skim milk dissolved in Tris-buffered saline (TBS) with 0.05% Tween 20 added (BioShop, Burlington, Canada, Cat. no.: TWN508) for 1 h at room temperature. Membranes were then incubated with primary antibodies at 4 ℃ overnight. In this study, we used antibodies against: glucocorticoid receptor (**GR**, Santa Cruz, Cat. no.: sc-1004), mineralocorticoid receptor (**MR**, Santa Cruz Biotechnology, Inc., Dallas, TX, USA, Cat. no.: sc-11412), phospho-cAMP response element-binding protein (**pCREB**, Millipore, Burlington, MA, USA, Cat. no.: 06-519), cAMP response element-binding protein (**CREB**, Millipore, Burlington, MA, USA, Cat. no.: 06-863), brain-derived neurotrophic factor (**BDNF**, Abcam, Cambridge, UK, Cat. no.: ab108319), glucocorticoid-induced leucine zipper protein (**GILZ**, Santa Cruz Biotechnology, Inc., Dallas, TX, USA, Cat. no.: sc-515835), serum/glucocorticoid-regulated kinase 1 (**SGK1**, Santa Cruz Biotechnology, Inc., Dallas, TX, USA, Cat. no.: sc-377360), methyl-CpG-binding protein 2 (**MeCP2**, Santa Cruz Biotechnology, Inc., Dallas, TX, USA, Cat. no.: sc-137070), histone deacetylase 1 (**HDAC1**, Santa Cruz Biotechnology, Inc., Dallas, TX, USA, Cat. no.: sc-81598), histone deacetylase 2 (**HDAC2**, Santa Cruz Biotechnology, Inc., Dallas, TX, USA, Cat. no.: sc-9959), histone deacetylase 4 (**HDAC4**, Santa Cruz Biotechnology, Inc., Dallas, TX, USA, Cat. no.: sc-46672), histone deacetylase 5 (**HDAC5**, Santa Cruz Biotechnology, Inc., Dallas, TX, USA, Cat. no.: sc-133106), DNA methyltransferase 1 (**Dnmt1**, Santa Cruz Biotechnology, Inc., Dallas, TX, USA, Cat. no.: sc-271729), DNA methyltransferase 3a (**Dnmt3a**, Santa Cruz Biotechnology, Inc., Dallas, TX, USA, Cat. no.: sc-365769), DNA methyltransferase 3b (**Dnmt3b**, Santa Cruz Biotechnology, Inc., Dallas, TX, USA, Cat. no.: sc-376043), phospho-insulin-like growth factor 1 (**p-IGF1R beta**, Invitrogen, Waltham, MA, USA, Cat. no.: PA5-37602), and insulin-like growth factor 1 (**IGF1R beta**, Invitrogen, Waltham, MA, USA, Cat. no.: AHO1292). The next day, excess antibodies were washed off the membranes by washing four times with TBS with 0.1% Tween 20 (TBS-T) (10 min each), followed by incubation with appropriate secondary antibody: horse anti-mouse or goat anti-rabbit IgG HRP peroxidase-conjugated secondary antibody (both Vector Laboratories, Peterborough, UK, Cat. no.: PI-2000 -1 and Cat no.: PI-1000, respectively) for 1 h at room temperature, and additional round of four washes in TBS-T (10 min each). The BM Chemiluminescence Western blotting Substrate (POD) (Roche, Mannheim, Germany, Cat. no.: 11500708001) was then used for the detection of the targeted protein bands and a luminescent image analyzer Fujifilm LAS-1000 System (Fujifilm, Tokyo, Japan) for their visualization. Densitometry measurements were applied for relative levels of protein concentration assessment and were conducted using Fujifilm Multi Gauge software (Fujifilm, Tokyo, Japan). In some cases, the membranes were subjected to further analysis. Therefore, they were stripped by shaking in stripping buffer (100 ml of Tris–HCl, pH = 6.7, 2% SDS, and 700 µl of 2-mercaptoethanol, all from Sigma-Aldrich, Saint Louis, MO, USA) for 30 min at 50 °C. After that, membranes were washed 3 times (10 min each) in TBS-T, blocked with 5% skim milk again, and reprobed with another primary antibody. Anti-β-actin (Sigma‒Aldrich, Saint Louis, MO, USA, Cat. no.: A5441) or anti-vinculin (Sigma-Aldrich, Saint Louis, MO, USA, Cat. no.: V9264) antibodies were used as an internal loading control. The original pictures of the membranes are included in the Supplementary Materials.

### Enzyme-linked immunosorbent assay

The levels of selected proteins level were measured in brain tissue homogenates using commercially available ELISA kits, according to the protocols provided by the manufacturers. They included **rat orexin A (**Bioassay Technology Laboratory, Shanghai, China, Cat. no.: E0105Ra), **orexin B (**Bioassay Technology Laboratory, Shanghai, China, Cat. no.: E1767Ra), **orexin receptor type 1** (Bioassay Technology Laboratory, Shanghai, China, Cat. no.: E3317Ra), **orexin receptor type 2** (Bioassay Technology Laboratory, Shanghai, China, Cat. no.: E33185Ra), FK506-binding protein 51 (**FKBP51**; ELK Biotechnology, Wuhan, China, Cat. no.: ELK0879), rat peroxisome proliferator-activated receptor gamma coactivator-1 alpha (**PGC1α**; Bioassay Technology Laboratory, Shanghai, China, Cat. no.: E2088Ra), sirtuin 1 (**SIRT1**; ELK Biotechnology, Wuhan, China, Cat. no.: ELK6192), phospho-AMP-activated protein kinase (**pAMPK**; Bioassay Technology Laboratory, Shanghai, China, Cat. no.: E0436Ra), AMP-activated protein kinase (**AMPK**; Bioassay Technology Laboratory, Shanghai, China, Cat. no.: E2094Ra), insulin growth factor 1 (**IGF-1**; ELK Biotechnology, Wuhan, China, Cat. no.: ER1486), **reelin** (ELK Biotechnology, Wuhan, China, Cat. no.: ELK6691), and fibronectin type III domain-containing protein 5 (**FNDC5**; Fine Test, Wuhan, China, Cat. no.: ER0563).

In the plasma samples, corticosterone levels (**CORT** Fine Test, Wuhan, China, Cat.: EU3108) were additionally assessed.

In each case, all samples along with standards, blanks, and positive controls were transferred to a 96-well pre-coated plate. Standard curves were used to calculate the analyzed marker concentrations, which were then presented as ng/g or ng/mg of protein.

### Statistical analysis and visualization

The obtained results were statistically evaluated using *Statistica 13.3* software. Student’s *t*-test for behavioral studies and two-way analysis of variance (ANOVA) followed by the Duncan post hoc test, when appropriate, for a biochemical part, were applied. Differences were considered significant at *p* < 0.05. The t-test results are reported as *t*-value and ANOVA results as an *F*-statistic and its associated degrees of freedom. All graphs were prepared using GraphPad Prism 8.

## Results

### Behavioral study

#### The effects of prenatal dexamethasone treatment on the memory processes of adult rats

To assess short-term memory, the NOR test was performed, and DEX-treated animals displayed a diminished discrimination index in comparison to control rats (*t* = 3.357; *p* = 0.004) (Fig. 2B). The same effect was observed in the NOL test—prenatal DEX administration resulted in a decrease in the discrimination index, which showed dysregulation in hippocampus-dependent spatial memory (*t* = 2.614; *p* = 0.018) (Fig. 2C).

### Biochemical study

#### The effects of dexamethasone treatment and acute stress on the hypocretin/orexin system—levels of orexin A and B proteins and their receptors, type 1 (OxR1) and type 2 (OxR2), in frontocortical and hippocampal tissue homogenates

**Fig. 3:**
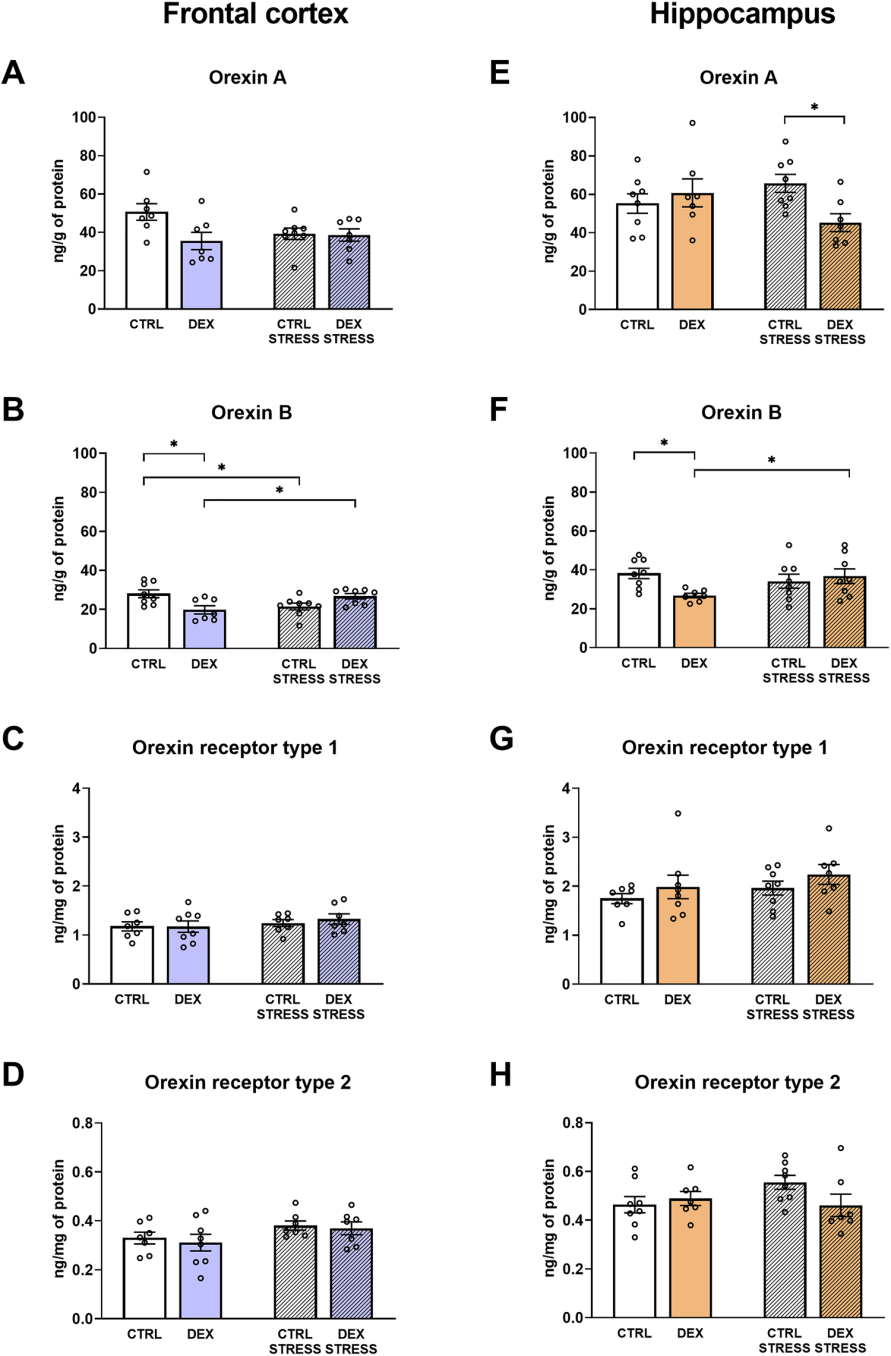
Effect of prenatal dexamethasone treatment and acute stress in adulthood on the level of orexin A (**A**) and B (**B**), as well as orexin receptor type 1 (**C**) and 2 (**D**) in the frontal cortex and hippocampus (**E**, **F**, **G**, and **H**, respectively) of male rats; the level of markers was assessed with ELISAs and expressed in ng/g and ng/mg of protein; bar graphs represent the mean ± SEM. Statistics: two-way ANOVA, followed by the Duncan post hoc test; *n* = 7–8; *p < 0.05; in the case of orexin A level in the frontal cortex, DEX main effect was significant at *F*_1,25_ = 4.352; *p* = 0.047)

When measuring orexin A in the frontal cortex, this hypocretin was decreased in DEX-treated rats (DEX effect *F*_1,25_ = 4.352; *p* = 0.047) (Fig. 3A). In the hippocampus, acute stress in adulthood resulted in the orexin A downregulation in DEX animals compared to similarly treated controls (DEX × stress effect *F*_1,26_ = 4.976; *p* = 0.035) (Fig. 3E). However, orexin B levels in the frontal cortex were diminished both by the DEX and stress (DEX × stress effect *F*_1,27_ = 13.791; *p* = 0.0009) (Fig. 3B), while in the hippocampus only in animals exposed to DEX in the prenatal period without the acute stress of immobilization in adult life, this factor was decreased (DEX × stress effect *F*_1,27_ = 5.078; *p* = 0.033). Moreover, in the DEX stressed group, orexin B was increased vs. adequate nonstressed rats in both examined brain areas (Fig. 3B, F).

No differences in receptor levels (OxR1, OxR2) were observed in either examined brain areas (Fig. 3C, D, G, and H).

#### The effects of dexamethasone treatment and acute stress on the AMPK-SIRT1-PGC1α pathway in frontocortical and hippocampal tissue homogenates

**Fig. 4:**
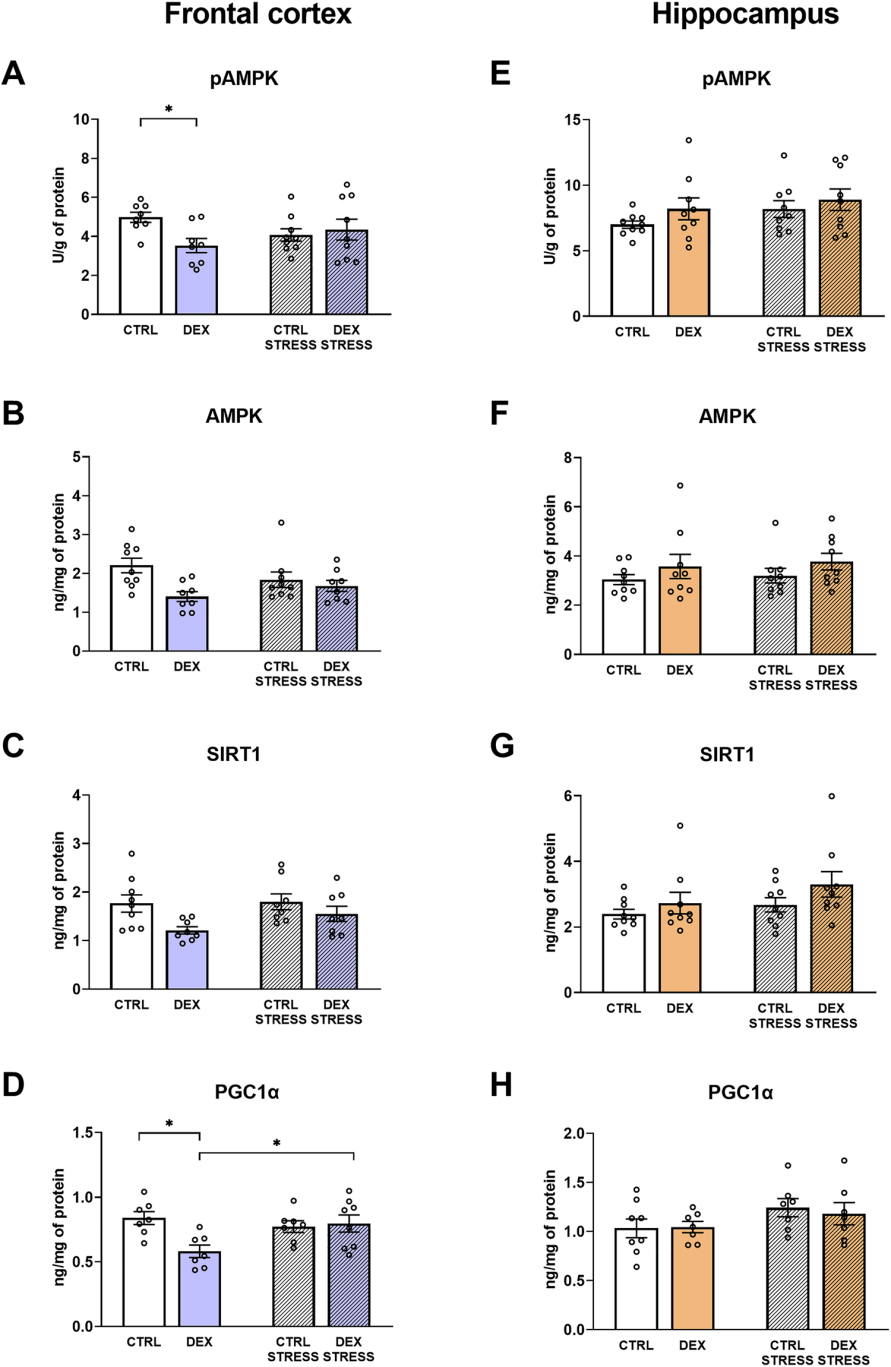
Effect of prenatal dexamethasone treatment and acute stress in adulthood on the level of phospho-AMPK (**A**), AMPK (**B**), deacetylase SIRT1 (**C**), and PGC1α receptor (**D**) in the frontal cortex and hippocampus (**E**, **F**, **G**, and **H**, respectively) of male rats; the level of proteins was assessed with ELISAs and expressed in U/g of protein (pAMPK) or ng/mg of protein (AMPK, SIRT1, and PGC1α); bar graphs represent the mean ± SEM. Statistics: two-way ANOVA, followed by the Duncan post hoc test; *n* = 7–9; *p < 0.05; in the case of AMPK and SIRT1 levels in the frontal cortex, DEX main effect was significant at *F*_1,30_ = 7.800; *p* = 0.009 and *F*_1,29_ = 7.051; *p* = 0.013, respectively

In the frontal cortex of rats prenatally treated with DEX, the phosphorylated form of AMPK was decreased (DEX × stress *F*_1,30_ = 4.838; *p* = 0.036) (Fig. 4A). The downregulation was exerted by DEX in the case of the total form of AMPK in this brain structure (DEX effect *F*_1,30_ = 7.800; *p* = 0.009) (Fig. 4B). Simultaneously, examining the frontocortical SIRT1 protein level the lowering effect of DEX was also demonstrated (DEX effect *F*_1,29_ = 7.051; *p* = 0.013) (Fig. 4C). PGC1α was lower in the DEX-treated group in comparison to the control, whereas after stress exposure this effect was not observed (DEX × stress effect F_1,25_ = 6.666; *p* = 0.016) (Fig. 4D).

In the hippocampus, the studied pathway was not affected by either DEX or stress (Fig. 4E, F, G, and H).

#### The effects of dexamethasone treatment and acute stress on the irisin precursor fibronectin type III domain-containing protein 5 (FNDC5), irisin, and brain-derived neurotrophic factor (BDNF) in frontocortical and hippocampal tissue homogenates

**Fig. 5:**
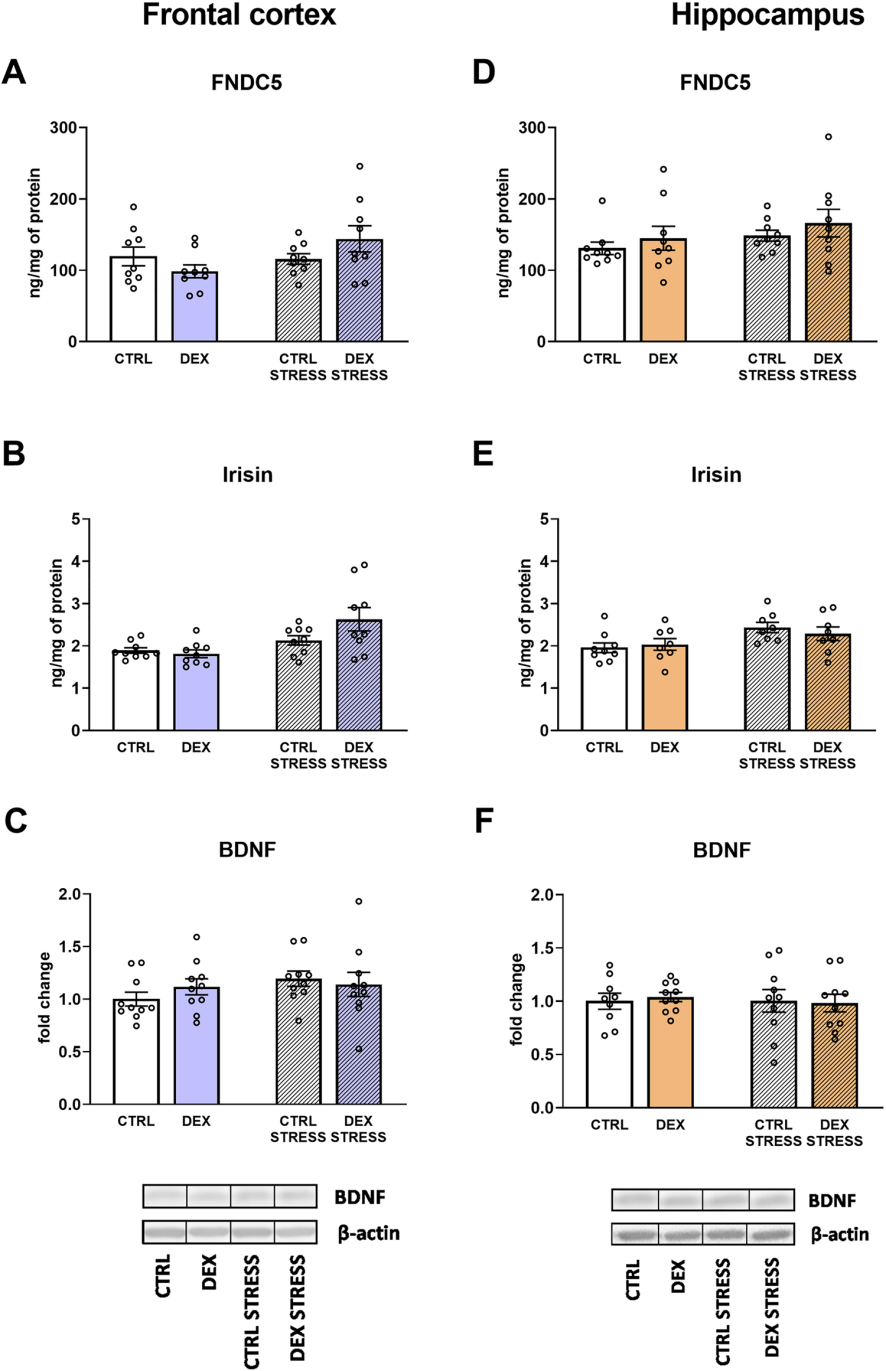
Effect of prenatal dexamethasone treatment and acute stress in adulthood on the level of FNDC5 protein (**A**), its cleaved form, irisin (**B**), and BDNF (**C**) in the frontal cortex and hippocampus (**D**, **E**, and **F**, respectively) of male rats; the level of the two former proteins was measured using ELISA and expressed in ng/mg of protein, and the latter was estimated by Western blot and expressed as a fold change; bar graphs represent the mean ± SEM. Statistics: two-way ANOVA; *n* = 8–10; in the case of irisin, stress main effect was significant at *F*_1,32_ = 11.096; *p* = 0.002 in the frontal cortex and *F*_1,29_ = 7.462; *p* = 0.011 in the hippocampus

No changes in FNDC5 protein level were observed in either studied brain structure (Fig. 5A, D), whereas the glycosylated polypeptide hormone that regulates energy metabolism, irisin, was increased by the stress in both examined brain areas (stress effect *F*_1,32_ = 11.096; *p* = 0.002 for frontal cortex; stress effect *F*_1,29_ = 7.462; *p* = 0.011for hippocampus) (Fig. 5B, E). BDNF levels did not differ between the measured groups in either examined brain area (Fig. 5C, F).

#### The effects of dexamethasone treatment and acute stress on insulin-like growth factor 1 (IGF-1) and the phosphorylated and total forms of its receptor in frontocortical and hippocampal tissue homogenates

**Fig. 6:**
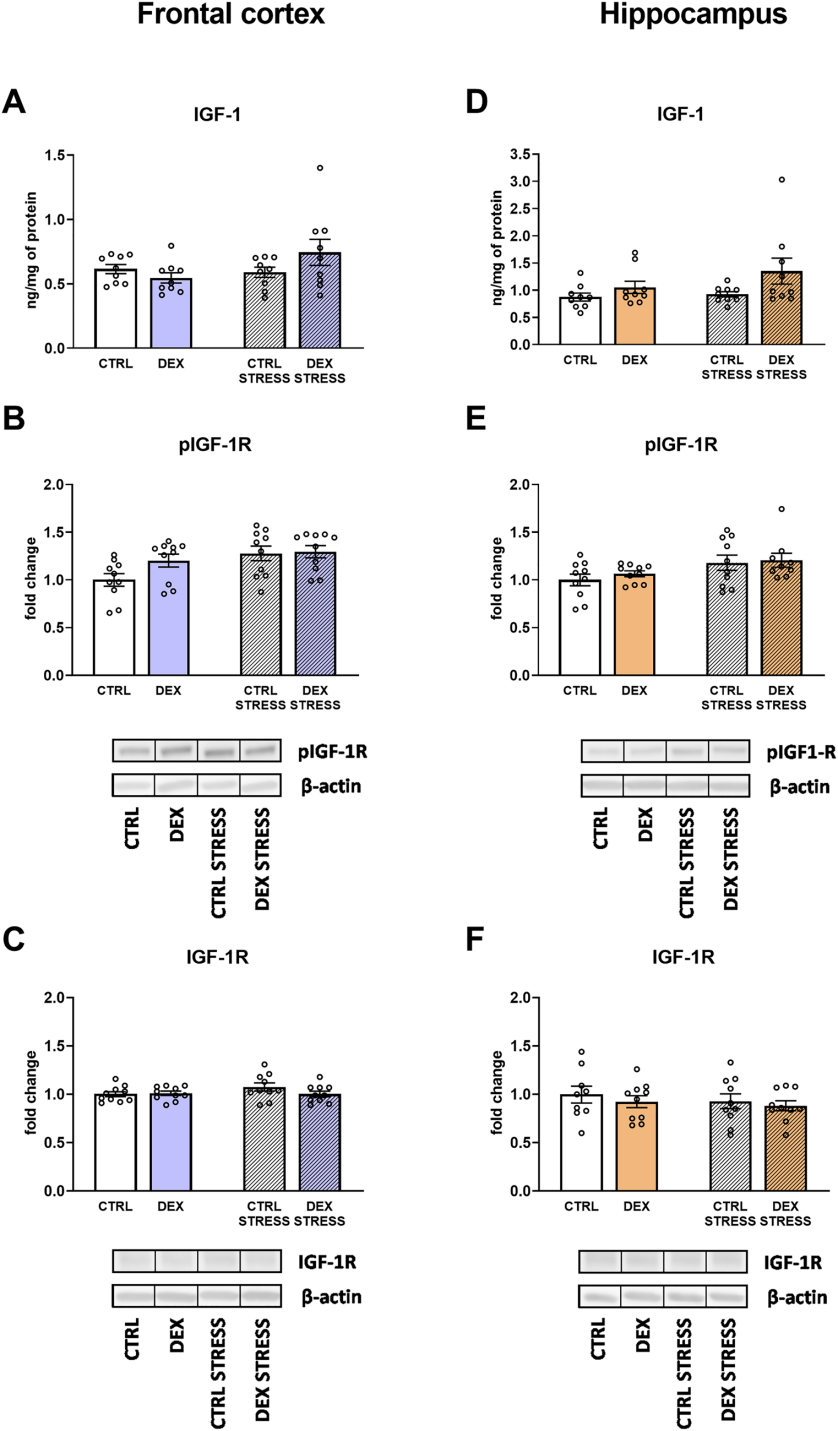
Effect of prenatal dexamethasone treatment and acute stress in adulthood on the level of IGF-1 (**A**) and its receptor phospho-IGF-1R (**B**) and total form IGF-1R (**C**) in the frontal cortex and hippocampus (**D**, **E**, and **F**, respectively) of male rats; the level of IGF-1 protein was assessed using ELISA and expressed in ng/mg of protein, while pIGF-1R and IGF-1R were estimated with Western blot and expressed as a fold change; bar graphs represent the mean ± SEM. Statistics: two-way ANOVA; *n* = 9–10; in the case of IGF-1 in the hippocampus, DEX main effect was significant at *F*_1,32_ = 4.722; *p* = 0.037, while stress effect in the case of pIGF-1R at *F*_1,36_ = 7.272; *p* = 0.011 in the frontal cortex and *F*_1,35_ = 6.364; *p* = 0.016 in the hippocampus

The level of IGF-1 in the frontal cortex did not differ between the groups, while in the hippocampus, DEX triggered an increase in this protein level (DEX effect *F*_1,32_ = 4.722; *p* = 0.037). (Fig. 6A, D).

In both the frontal cortex and the hippocampus, a stress-induced increase in p-IGF1R was observed (stress effect *F*_1,36_ = 7.272; *p* = 0.011 for frontal cortex, *F*_1,35_ = 6.364; *p* = 0.016 for hippocampus) (Fig. 6B, E). Levels of total IGF-1R did not differ between the groups (Fig. 6C, F).

#### The effects of dexamethasone treatment and acute stress on cAMP response element-binding protein (CREB) in phosphorylated and total forms in frontocortical and hippocampal tissue homogenates

**Fig. 7:**
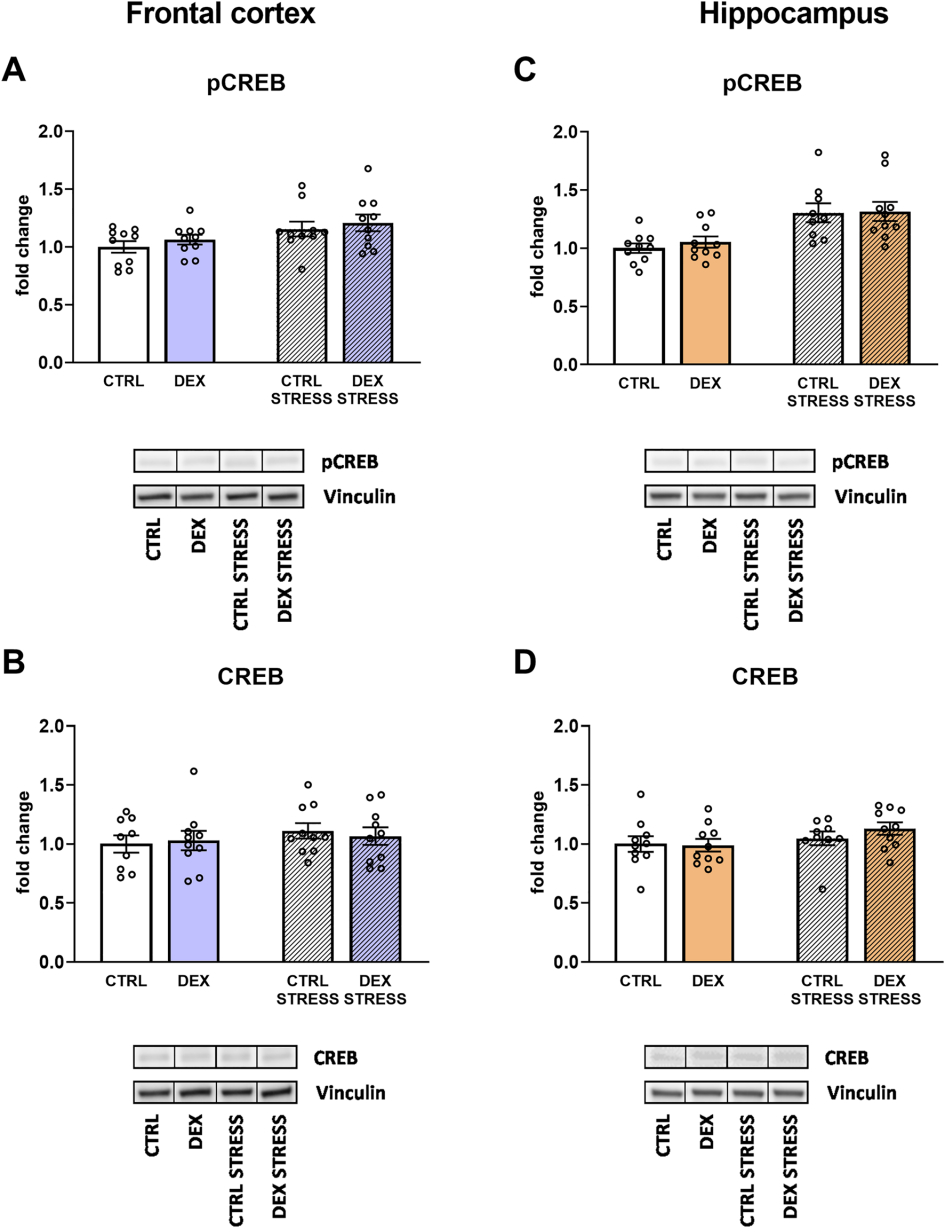
Effect of prenatal dexamethasone treatment and acute stress in adulthood on the level of phospho-CREB (**A**) and CREB transcription factor (**B**) in the frontal cortex and hippocampus (**C** and **D**, respectively) of male rats; the level of pCREB and CREB was estimated with Western blot and expressed as a fold change; bar graphs represent the mean ± SEM. Statistics: two-way ANOVA; *n* = 9–10; in the case of pCREB, stress main effect was significant at *F*_1,36_ = 6.541; *p* = 0.015 in the frontal cortex and *F*_1,35_ = 18.844; *p* = 0.0001 in the hippocampus

Upon measuring the level of pCREB, the effect of stress was established in both studied brain areas, and stress in adulthood led to an increase in pCREB (stress effect *F*_1,36_ = 6.541; *p* = 0.015 for frontal cortex; *F*_1,35_ = 18.844; *p* = 0.0001 for hippocampus) (Fig. 7A, C). No changes were detected in the total form of this transcription factor in the frontal cortex or the hippocampus (Fig. 7B, D).

#### The effects of dexamethasone treatment and acute stress on HPA function—plasma corticosterone level, GR, MR, and their regulatory factors—FKBP51, SGK1, and GILZ - in frontocortical and hippocampal tissue homogenates

To assess whether DEX alters glucocorticoid levels in the brain, the concentrations of corticosterone in the plasma and GR and MR in the tissues were measured.

**Fig. 8:**
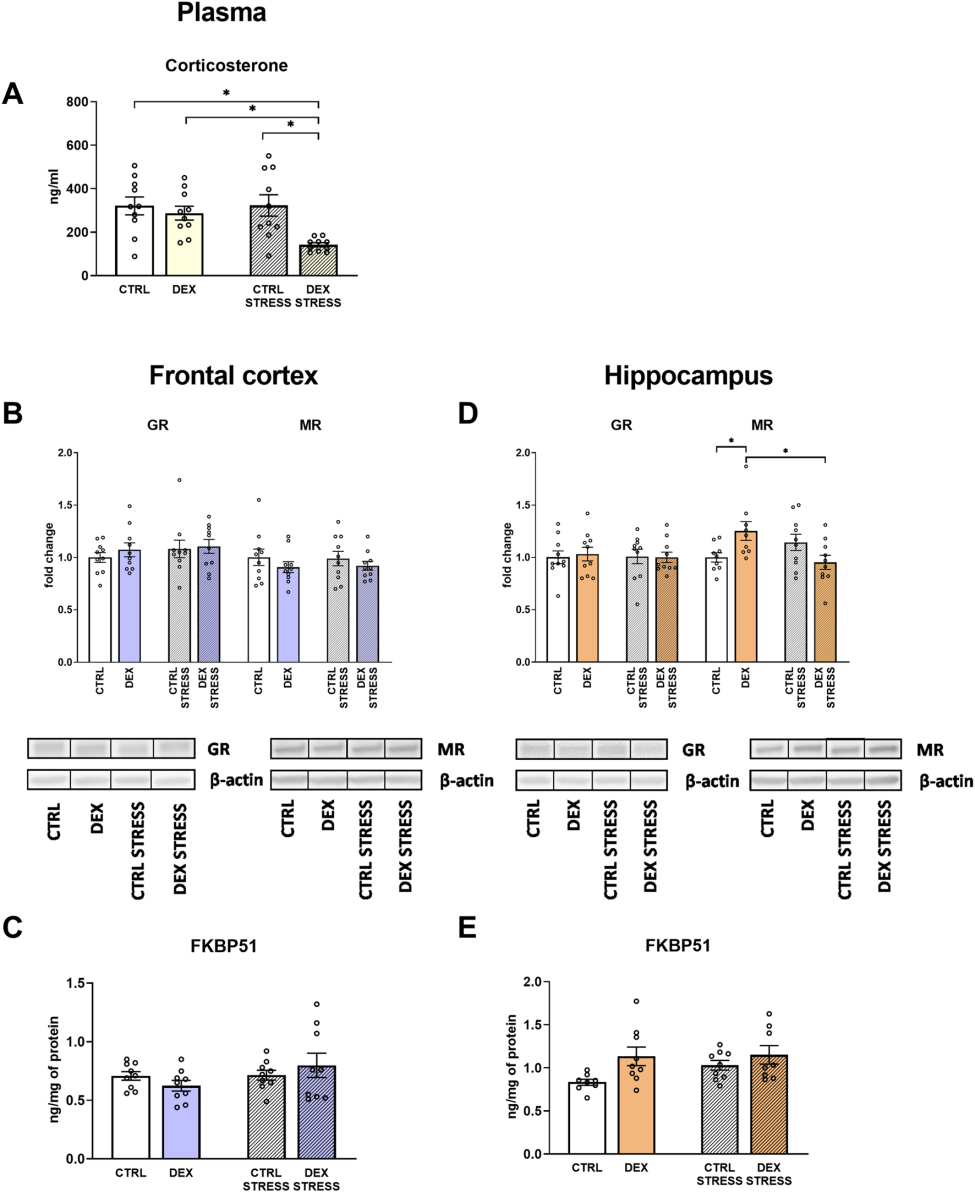
Effect of prenatal dexamethasone treatment and acute stress in adulthood on the level of corticosterone in the serum (**A**), as well as glucocorticoid and mineralocorticoid receptors (**B**), and FKBP1 protein (**C**) in the frontal cortex and hippocampus (**D** and **E**, respectively) of male rats; the level of corticosterone and FKBP1 protein was measured using ELISA and expressed in ng/ml and ng/mg of protein, respectively, while glucocorticoid and mineralocorticoid receptors level was determined with Western blot and expressed as a fold change; bar graphs represent the mean ± SEM. Statistics: two-way ANOVA, followed by the Duncan post hoc test; *n* = 8–10; *p < 0.05; in the case of FKBP51 in the hippocampus, DEX main effect was significant at *F*_1,30_ = 6.419; *p* = 0.017

The corticosterone level in plasma was diminished in rats prenatally treated with DEX and stressed in adulthood in comparison to all other groups (DEX × stress effect *F*_1,36_ = 4.194; *p* = 0.048) (Fig. 8A).

No differences between the groups were shown for GR concentration in the frontal cortex or the hippocampus (Fig. 8B, D). MR levels differed between the DEX and control groups only in the hippocampus; DEX-treated animals displayed an increase in the measured receptor level, whereas after the stress procedure, this effect was reversed (DEX × stress effect *F*_1,34_ = 9.307; *p* = 0.004) (Fig. 8B, D).

No changes in the level of FKBP51 were shown in the frontal cortex, whereas its elevation was observed in DEX-treated animals in the hippocampus (DEX effect *F*_1,30_ = 6.419; p = 0.017) (Fig. 8C, E).

**Fig. 9:**
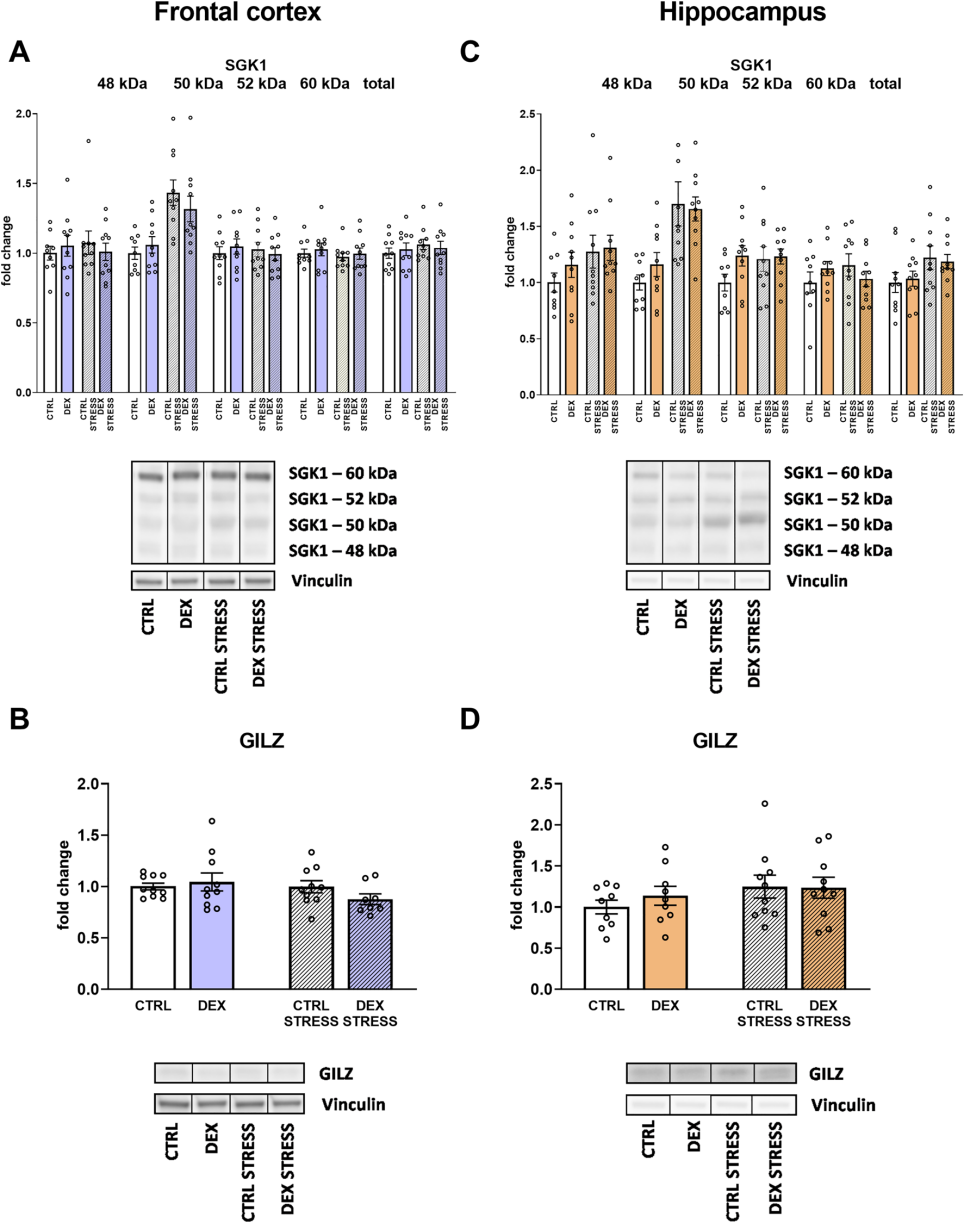
Effect of prenatal dexamethasone treatment and acute stress in adulthood on the level of different forms (48, 50, 52, and 60 kDa) and total SGK1 kinase (**A**), as well as GILZ protein (**B**) in the frontal cortex and hippocampus (**C** and **D**, respectively) of male rats; the level of proteins was determined with Western blot and expressed as a fold change; bar graphs represent the mean ± SEM. Statistics: two-way ANOVA; *n* = 8–10; in the case of isoform 50 kDa, stress main effect was significant *F*_1,35_ = 20.786; *p* = 0.00006 in the frontal cortex and *F*_1,35_ = 20.740; *p* = 0.00006 in the hippocampus; moreover, regarding the total form of SGK1 in the hippocampus, stress main effect was significant at *F*_1,36_ = 5.060; *p* = 0.031

Then, we measured the total and isoforms (48, 50, 52, and 60 kDa) of SGK1 protein, and in both brain areas, acute stress triggered the increase in the level of 50 kDa isoform (stress effect (*F*_1,35_ = 20.786; *p* = 0.00006 for frontal cortex and stress effect *F*_1,35_ = 20.740; *p* = 0.00006 for hippocampus) (Fig. 9A, C). Additionally, in the case of the hippocampus, the effect of stress was also observed in the level of total form of the SGK1 protein (stress effect *F*_1,36_ = 5.060; *p* = 0.031) (Fig. 9C).

No differences in glucocorticoid-induced leucine zipper (GILZ) were detected in either the frontal cortex or the hippocampus (Fig. 9B, D).

#### The effects of dexamethasone treatment and acute stress on MeCP2 levels in the nuclear fraction of the frontal cortex and hippocampus

**Fig. 10:**
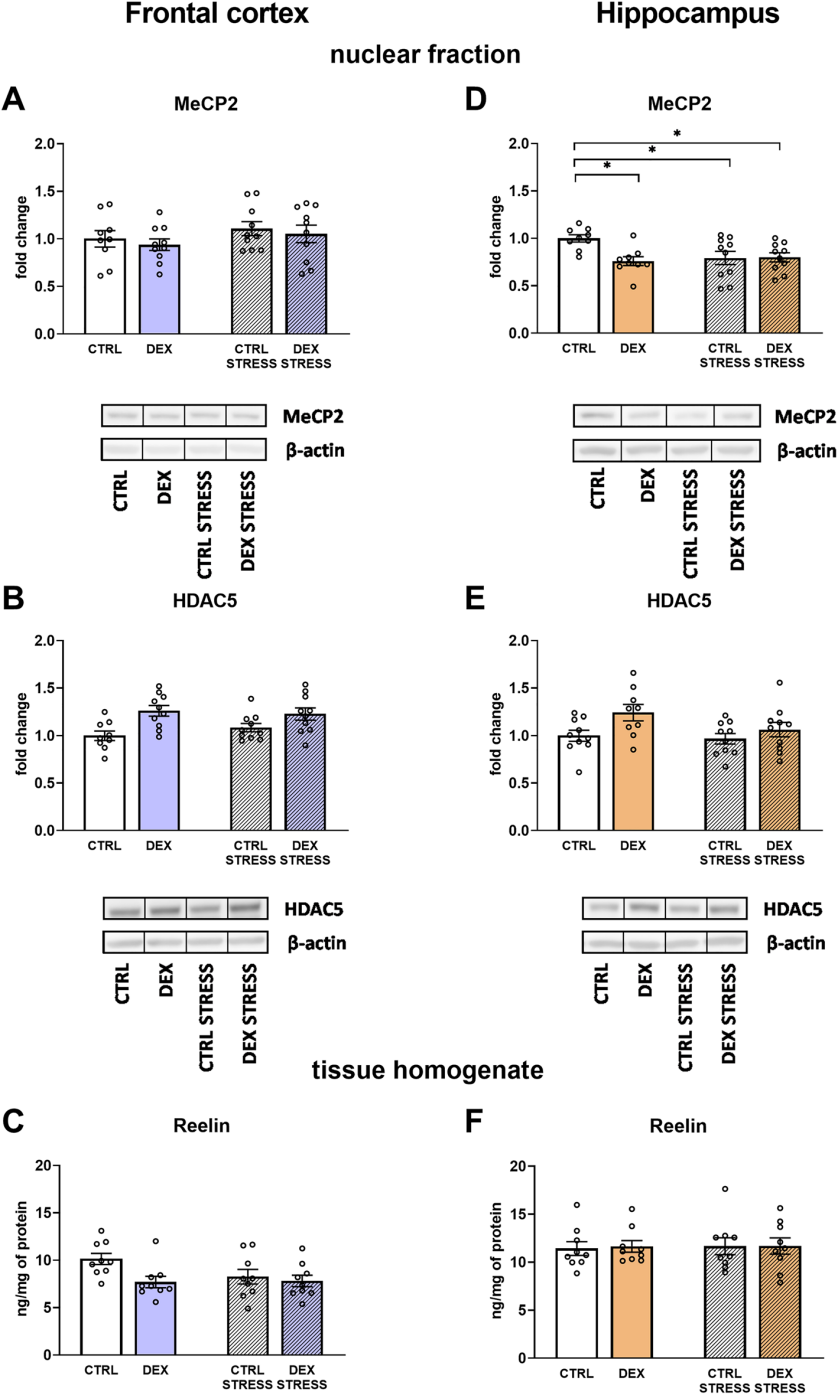
Effect of prenatal dexamethasone treatment and acute stress in adulthood on the level of MeCP2 (**A**) and HDAC5 (**B**) in the nuclear fraction and reelin glycoprotein in tissue homogenate (**C**) in the frontal cortex and hippocampus (**D**, **E**, and **F**, respectively) of male rats; the level of proteins was assessed with Western blot (MeCP2 and HDAC5) or ELISA (reelin) and expressed as fold change or in ng/mg of protein (reelin); bar graphs represent the mean ± SEM. Statistics: two-way ANOVA, followed by the Duncan post hoc test; *n* = 9–10; *p < 0.05; in the case of HDAC5, DEX main effect was significant at *F*_1,35_ = 13.786; *p* = 0.0007 in the frontal cortex and *F*_1,35_ = 6.068; *p* = 0.019 in the hippocampus; also, regarding reelin in the frontal cortex, DEX main effect was significant at *F*_1,32_ = 4.976; *p* = 0.033

In the frontal cortex, no changes in the level of MeCP2 were observed (Fig. 10A), whereas in the hippocampus, both stress and DEX led to downregulation of this methyl-CpG-binding protein level (DEX × stress effect *F*_1,34_ = 5.444; *p* = 0.026) (Fig. 10D).

#### The effects of dexamethasone treatment and acute stress on HDAC 1, 2, 4, and 5 protein levels and Dnmt1, 3a, and 3b in the nuclear fraction of the frontal cortex and hippocampus

The levels of HDAC1, HDAC2, and HDAC4 did not differ between the examined groups in either the frontal cortex or the hippocampus (Table 1).

**Table 1:**
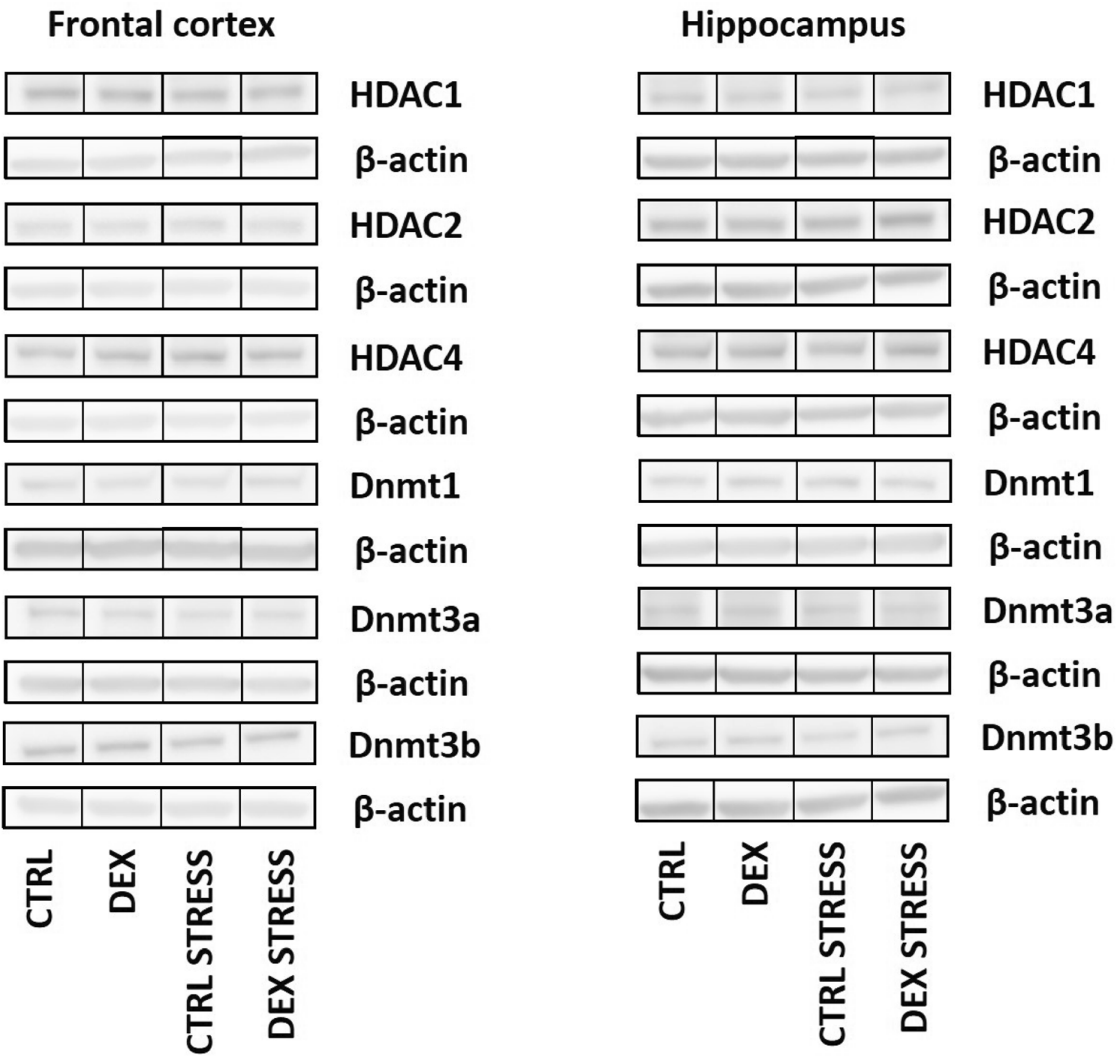
Effect of prenatal dexamethasone treatment and acute stress in adulthood on the level of selected proteins involved in epigenetic control. The levels of HDAC1, HDAC2, HDAC4, Dnmt1, Dnmt3a, and Dnmt3b were measured by Western blotting in the isolated nuclear fraction of frontal cortex and hippocampus and expressed as the mean fold change ± SEM. Statistics: two-way ANOVA; *n* = 8–10

Regarding the deacetylase HDAC5 level, it was significantly increased in the frontal cortex (DEX effect *F*_1,35_ = 13.786; *p* = 0.0007) (Fig. 10B) and in the hippocampus (DEX effect *F*_1,35_ = 6.068; *p* = 0.019) of the prenatally DEX-treated animals (Fig. 10E).

No changes in the protein concentration between the groups were disclosed for all DNA methyltransferases in the studied brain structures (Table 1).

#### The effects of dexamethasone treatment and acute stress on reelin levels in frontocortical and hippocampal tissue homogenates

Studies concerning the concentration of the reelin, protein involved in the processes of synaptic plasticity, showed a significant decrease in the level of this protein in the frontal cortex of animals whose mothers were given DEX during the pregnancy (DEX effect *F*_1,32_ = 4.976; *p* = 0.033) (Fig. 10C). No such dependency was found in the hippocampus (Fig. 10F).

## Discussion

Many studies indicate that stress or excess exposure to glucocorticoids in the prenatal or early postnatal period increases the risk of developing depression later in life. Adverse factors operating in prenatal life can lead to many long-lasting changes, such as impaired behavior, cognitive function, HPA axis activity alterations, neurotransmitter and neuropeptide level modification, and disturbances in synaptic plasticity and metabolic processes in the brain [25]. However, little is yet known about which glucocorticoid-induced changes are responsible for increasing the risk of depression in the offspring and whether they could be a target for pharmacotherapy of this disease in future.

Our and other authors’ studies indicate that both in the pathogenesis of depression and in the prodepressant effect of glucocorticoids, metabolic changes in the brain play an important role [26–30]. In the current study conducted in an animal model of depression based on the administration of dexamethasone (DEX) in the last week of gestation in a rat, we investigated the mechanisms involved in the regulation of important metabolic pathways in the brain in male offspring. It has been demonstrated that prenatal exposure to DEX decreased orexin A and B levels in the frontal cortex and orexin B levels in the hippocampus; however, it did not affect the level of receptors for these neuropeptides in any of the examined brain structures. Consistent with these results, lowered levels of orexins were found in both animal models of depression and patients with depressive disorders [31]. Orexin neurons are known to play an important role in stress-induced psychiatric disorders, including depression and posttraumatic stress disorder (PTSD). They contribute to the regulation of metabolic processes, the sleep/wake rhythm, emotions, the dopaminergic reward system, and learning and memory processes [32]. In the model of depression used in this study, the reduced level of orexins can be associated both with the previously demonstrated depression-like and anxiety-like behaviors in animals exposed to DEX [23] and with the finding of the present study that prenatal administration of DEX caused disturbances of cognitive processes, as determined in the NOL and NOR tests. We have shown that prenatal exposure to DEX resulted in impairment of both spatial memory, which is mainly dependent on hippocampal function and determined by the NOL test, and deficits in recognition memory, tested by the NOR test, in which the integration of the hippocampus and cortical areas plays an important role. The demonstrated memory impairment may be due to the downregulation of the orexin system, which is essential for the consolidation of hippocampus-dependent memory [33]. Peripheral and intrahippocampal administration of orexin has been found to improve cognition in experimental animals, and these effects are mainly associated with the enhancement of long-term potentiation [34, 35]. The neuroprotective effect of orexins is associated mainly with the inhibition of neuroinflammation, but these peptides also play a crucial role in the regulation of energy metabolism, and their reduced function contributes to obesity [36]. Downregulation of orexin signaling is implicated not only in the pathogenesis of narcolepsy but also possibly in other neurodegenerative diseases, such as Parkinson’s, Alzheimer’s, and Huntington’s diseases [37]. In the frontal cortex of animals prenatally exposed to DEX, we observed not only a decrease in orexin levels but also a decline in the levels of AMPK, sirtuin 1 (SIRT1), and PGC1α, which are important regulators of mitochondrial metabolism and mitochondrial biogenesis. These data confirm both our previous research and studies conducted by other authors indicating that depression leads to metabolic disturbances in the brain, mainly mitochondrial dysfunction. We have previously shown a decrease in oxidative phosphorylation and ATP production in the rat frontal cortex in the same model of depression as used in this study [23]. Thus, these observations may result from the downregulation of PGC1α, an important regulator of oxidative phosphorylation and mitochondrial biogenesis, and from reducing the levels of AMPK and SIRT1, key factors regulating PGC1α. SIRT1 is a metabolic-sensor protein that is significantly involved in adequate adaptation to fasting and influences several signaling pathways by deacetylating PGC1 to regulate its expression and activity. Thus, prenatal exposure to DEX may impair mitochondrial function and consequently ATP production via inhibition of the AMPK-SIRT1-PGC1α signaling pathway.

The second studied pathway, FNDC5/irisin/BDNF, which can be stimulated by PGC1α and physical activity, did not seem to play a significant role in disturbances occurring in the used model of depression. We showed no changes in the levels of FNDC5, the precursor of irisin and mature irisin, in animals prenatally exposed to DEX, and stress in adulthood even increased levels of this myokine. In the brain, irisin plays an important role in synaptic plasticity, memory processes, and exercise-induced neurogenesis [38]. FNDC5/irisin increases brain levels of BDNF, and physical activity has been shown to improve cognitive functions mainly by increasing the expression of FNDC5/irisin and BDNF in the hippocampus [39]. As in the case of FNDC5 and irisin, the level of BDNF was not reduced in any of the examined brain structures in animals prenatally exposed to DEX. This is an unexpected result, as reduced expression of FNDC5/irisin, and BDNF has been observed in patients suffering from depression and in some animal models of depression [39, 40]. However, the previously observed changes in FNDC5 levels depend on the animal model of depression used, and, for example, in the model induced by administration of corticosterone, even increased expression of FNDC5 was observed in the frontal cortex in contrast to the decrease in this factor in the model based on LPS administration. These inconsistencies were explained by other prodepressive behaviors observed in these models [40].

Similar to the lack of downregulation of BDNF, we also did not show a lowering of the level of another growth factor tested, i.e., IGF-1, or the transcription factor CREB in animals prenatally exposed to DEX. Interestingly, stress in adult animals even increased the levels of active, phosphorylated forms of both the IGF receptor and CREB factor. These results are inconsistent with the decreases in pCREB and BDNF levels, as well as the weakening of IGF-1 activity in the brain, often shown in various models of depression. However, data on the reduction of neurotrophin levels in depression and their role in the pathogenesis of this disease, including disturbances of synaptic plasticity, cell metabolism, and the development of mood disorders, are inconclusive [41]. Most animal models of depression are based on the application of mild, long-term/chronic stress, but its effects depend on many factors, such as the period of life over which they act, which may explain these discrepancies.

In the next step, we wanted to determine whether prenatal exposure to DEX could alter the level of corticosterone receptors. In the frontal cortex, we did not detect any changes in the levels of glucocorticoid receptors (GRs), which are primarily involved in the stress response, mineralocorticoid receptors (MRs), which are mainly involved in the maintenance of the basal activity of the HPA axis, or FK506-binding protein 51 (FKBP51), which inhibits GR function. In the hippocampus, a DEX-dependent increase in the FKBP51 level suggests a weakening of GR function, and the simultaneous increase in the MR concentration indicates that there was a change in the MR/GR balance in this brain structure, a change that, according to the current state of knowledge, should shift the action of GCs toward neuroprotection. This is because many studies have shown that the weakening of MR function causes degeneration of the granule cells of the dentate gyrus of the hippocampus, while excessive GR stimulation hurts the morphology and function of neurons [42–44]. However, changes in GR target gene products did not confirm a reduction in GR function in the hippocampus. Since GR function depends, in addition to the FKBP51 protein, on many factors, it is currently recommended to evaluate their function by determining the expression of their target genes, which include glucocorticoid-induced leucine zipper (GILZ) or serum/glucocorticoid-regulated kinase 1 (SGK1). The fact that the products of these genes were not changed in any of the examined brain structures indicates that prenatal exposure to DEX did not cause permanent changes in GR function. An increase in one of the SGK1 isoforms in both examined brain structures was observed only after stress, both in control animals and those exposed to DEX.

By measuring blood corticosterone levels, we found that animals receiving DEX prenatally, in adulthood, one hour after acute stress, had significantly lower corticosterone levels than animals not exposed to that steroid. A frequently observed modification in animal models of depression is an increased level of corticosterone after stress, lasting longer than in control animals, which indicates a weakening of the feedback mechanism regulating the activity of the HPA axis. The weakening of this mechanism may result from lower GR function not only in the hypothalamus but also in the frontal cortex and hippocampus. The decrease in corticosterone levels observed in the present study suggests a stronger inhibitory effect of GR on corticoliberin synthesis, but this excludes the fact that GR function, as determined by SGK1 levels, was similarly elevated in the hippocampus and frontal cortex after stress in control animals and animals prenatally treated with DEX. We did not investigate GR function in the hypothalamus, the main structure involved in regulating the HPA axis, but GR levels in the hypothalamus are less likely to be altered than in the frontal cortex and hippocampus, so it is more feasible that the observed reduction in corticosterone levels may be due to a direct effect of DEX on the adrenal glands. Prenatal administration of DEX by activating the GR in the adrenal gland has been shown to cause adrenal insufficiency in male rats [45]. However, in Chen et al. (2022), DEX was administered at higher doses than in the current research, so it is possible that for this reason, we observed a decrease in corticosterone concentration only after stress but not under basal conditions.

Unfavorable factors acting in the prenatal and/or early postnatal period may cause epigenetic changes that result in long-term effects on neuronal function and, as a consequence, increase the risk of depression development later in life. It has also been shown that prenatal exposure to DEX can lead to modifications of DNA and histone proteins and affect gene transcription [46, 47]. We have shown that prenatal exposure to DEX, among the tested histone deacetylases, increased the level of HDAC5 in both examined brain structures. Importantly, increased expression of HDAC5 protein has already been found in an animal model of depression, and lowering the level of this enzyme by antidepressants is critical for their therapeutic effect [48, 49]. Moreover, in rats prenatally exposed to DEX, the downregulation of adrenal corticosterone synthesis was associated with upregulation of HDAC5 and its inhibition of transcription of steroidogenic factor 1 [45]. Thus, the elevated HDAC5 level demonstrated in the present study in animals receiving DEX prenatally is a significant biochemical change observed both in leukocytes in depressed individuals and the brain in animal models of these diseases, and this alteration may be involved in the behavioral and biochemical abnormalities found in depression. In addition, if the level of this enzyme is also increased in the adrenal glands, it may contribute to the hypofunction of this gland [45]. Prenatal administration of DEX did not change the level of any of the tested DNA methyltransferases, while in the hippocampus, it decreased the concentration of methyl-CpG-binding protein 2 (MeCP2), which is a transcription regulator particularly highly expressed in the brain, especially in neurons. MeCP2 binds methylated CpG dinucleotides and forms a complex with histone deacetylase, which triggers chromatin structure remodeling by histone deacetylation and represses transcription. MeCP2 is downregulated in many stress-induced psychiatric disorders and is thought to be a marker of vulnerability to stress, and a link between MeCP2 and early stress experiences has been suggested [50]. Among other things, it has been shown that the binding of MeCP2 and Dnmt1 to the promoter of the gene encoding reelin leads to the inhibition of the synthesis of this protein, which is important in the process of neuronal migration during the developmental period and synaptic plasticity and neurogenesis in the adult brain [51, 52]. However, in our study, a decreased level of reelin after DEX administration was observed in the frontal cortex, while the decrease in MeCP2 in the hippocampus and the level of Dnmt1 did not change; therefore, the reduction in reelin concentration in the frontal cortex is unlikely to be related to MeCP2. The reduction in reelin levels detected in the frontal cortex may be important in inducing the behavioral abnormalities found in this model of depression since such a relationship exists in people with depression. Patients with neuropsychiatric disorders have been shown to have reduced expression of reelin, while an increase in its expression improves neurological functions, which makes reelin a promising therapeutic target in the treatment of neuropsychiatric disorders in future [53].

In conclusion, the present study showed that prenatal exposure to DEX decreased the levels of orexin A and B and the AMPK-SIRT1-PGC1α transduction pathway in the frontal cortex, which may be the reason for the decrease in mitochondrial metabolism and ATP production observed in our previous study [23]. Moreover, a decrease in reelin levels in the frontal cortex may contribute to impaired synaptic plasticity and the process of neurogenesis and, consequently, the behavioral disturbances observed in depression. In contrast to the frontal cortex, in the hippocampus, only orexin B was reduced, and changes in the MR/GR ratio suggest the induction of a neuroprotective mechanism. This is consistent with our and other authors’ observations that in stress- or glucocorticoid-related models of depression, metabolic and/or neurodegenerative changes occur mainly in the frontal cortex, while in the hippocampus, they are weaker because adaptive mechanisms are induced. Moreover, prenatal exposure to DEX induced an increase in HDAC5 levels in both examined brain structures and a decrease in MeCP2 in the hippocampus, which are changes typical of depression and susceptibility to stress, but their impact on the transcription of specific genes in this model of depression will be the subject of further research.

### Supplementary Information

Below is the link to the electronic supplementary material.

Supplementary file1 (PDF 6690 KB)

## Data Availability

The datasets generated during this study are available from the corresponding author upon reasonable request from qualified researchers.

## Abbreviations

AMPK: AMP-activated protein kinase
ATP: Adenosine triphosphate
BDNF: Brain-derived neurotrophic factor
CNS: Central nervous system
CORT: Corticosterone
CREB: CAMP response element-binding protein
DEX: Dexamethasone
Dnmt: DNA methyltransferase
DTT: Dithiothreitol
FKBP51: FK506-binding protein 51
FNDC5: Fibronectin type III domain-containing protein 5
GCs: Glucocorticoids
GILZ: Glucocorticoid-induced leucine zipper protein
GR: Glucocorticoid receptor
HDAC: Histone deacetylase
HPA: Hypothalamic–pituitary–adrenal axis
IGF1: Insulin-like growth factor 1
IGF1R: Insulin-like growth factor 1 receptor
LPS: Lipopolysaccharide
MeCP2: Methyl-CpG-binding protein 2
MR: Mineralocorticoid receptor
PGC1α: Peroxisome proliferator-activated receptor gamma coactivator 1-alpha
PPARγ: Proliferator-activated receptor gamma
ROS: Reactive oxygen species
sGC: Synthetic glucocorticoids
SGK1: Serum/glucocorticoid-regulated kinase 1
SIRT1: Sirtuin 1
TBS: Tris-buffered saline
