# Supplementary material for: Contribution of changes in the orexin system and energy sensors in the brain in depressive disorder - a study in an animal model
Source: Pharmacol Rep. 2024 Jan 9;76(1):51–71. doi: 10.1007/s43440-023-00559-0 (PMC10830606; doi:10.1007/s43440-023-00559-0)
Supplement: Supplementary file 1 — Supplementary file1 (PDF 6690 KB) [file 43440_2023_559_MOESM1_ESM.pdf]

Supplementary materials

Western blot membranes

BDNF

Frontal cortex

membrane 1 and membrane 2

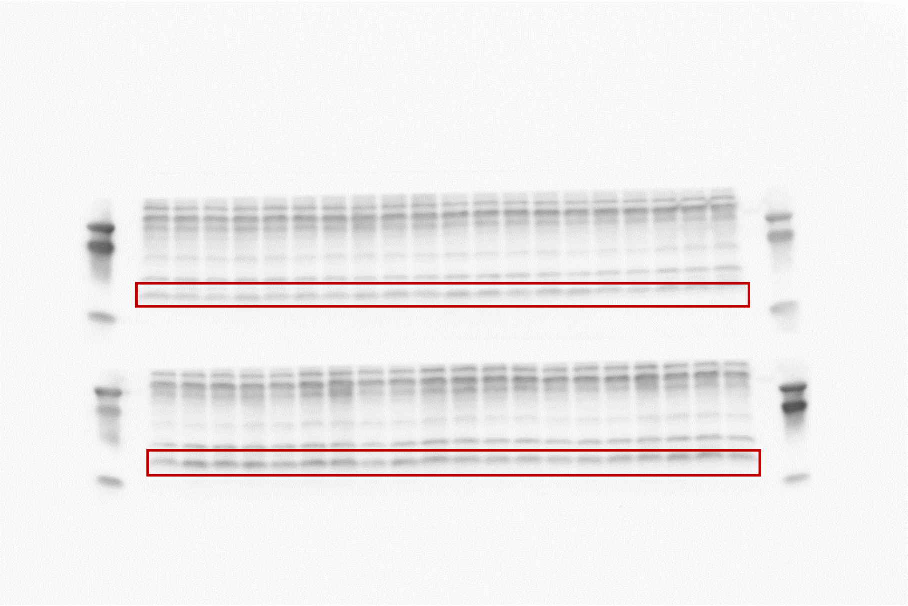

Samples from the left side - membrane 1:
CTRL, CTRL + STRESS, DEX, DEX + STRESS, CTRL, CTRL + STRESS, DEX, DEX + STRESS, CTRL, CTRL + STRESS, DEX, DEX + STRESS, CTRL, CTRL + STRESS, DEX, DEX + STRESS, CTRL, CTRL + STRESS, DEX, DEX + STRESS.

Samples from the left side - membrane 2:
CTRL, CTRL + STRESS, DEX, DEX + STRESS, CTRL, CTRL + STRESS, DEX, DEX + STRESS, CTRL, CTRL + STRESS, DEX, DEX + STRESS, CTRL, CTRL + STRESS, DEX, DEX + STRESS, CTRL, CTRL + STRESS, DEX, DEX + STRESS.

The results of the experiment are shown in Figure 5.

β-actin as loading control to BDNF

Frontal cortex

membrane 1 and membrane 2

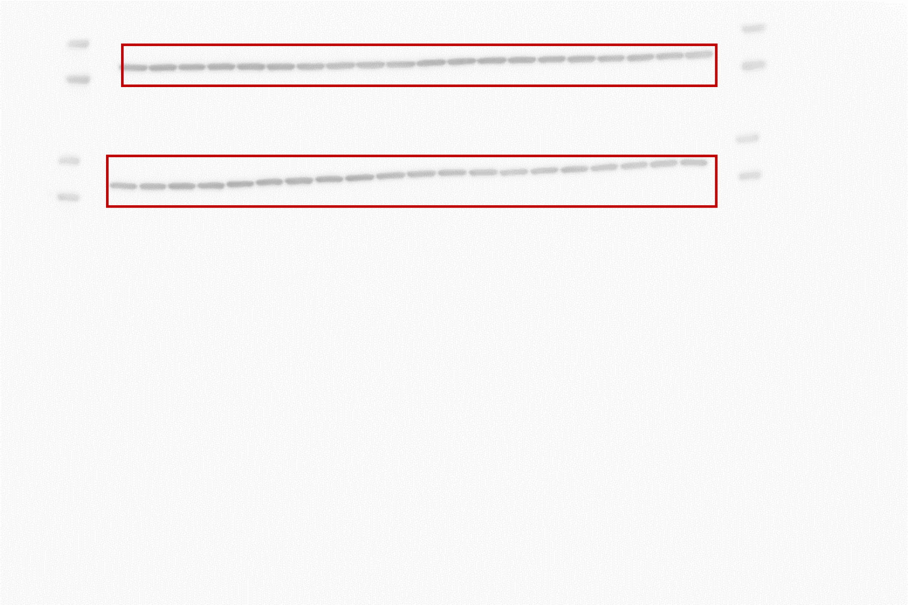

BDNF

Hippocampus

membrane 1 and membrane 2

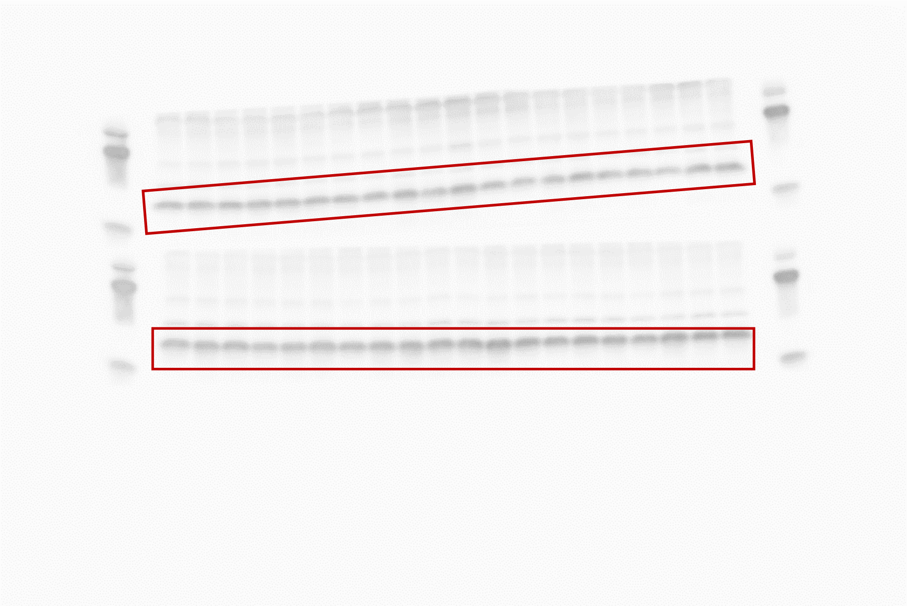

Samples from the left side - membrane 1:
CTRL, CTRL + STRESS, DEX, DEX + STRESS, CTRL, CTRL + STRESS, DEX, DEX + STRESS, CTRL, CTRL + STRESS, DEX, DEX + STRESS, CTRL, CTRL + STRESS, DEX, DEX + STRESS, CTRL, CTRL + STRESS, DEX, DEX + STRESS.

Samples from the left side - membrane 2:
CTRL, CTRL + STRESS, DEX, DEX + STRESS, CTRL, CTRL + STRESS, DEX, DEX + STRESS, CTRL, CTRL + STRESS, DEX, DEX + STRESS, CTRL, CTRL + STRESS, DEX, DEX + STRESS, CTRL, CTRL + STRESS, DEX, DEX + STRESS.

The results of the experiment are shown in Figure 5.

β-actin as loading control to BDNF

Hippocampus

membrane 1 and membrane 2

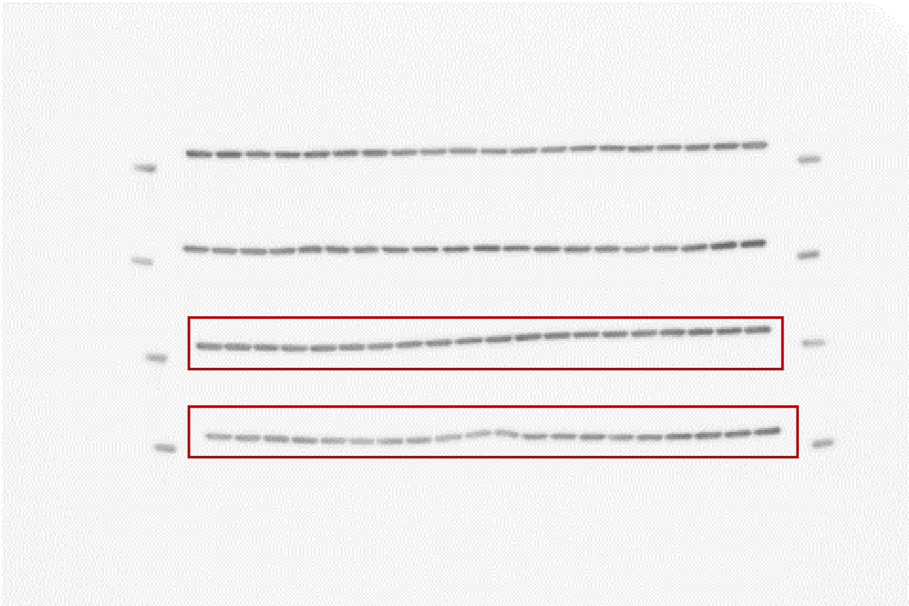

pIGF1-R

Frontal cortex

membrane 1 and membrane 2

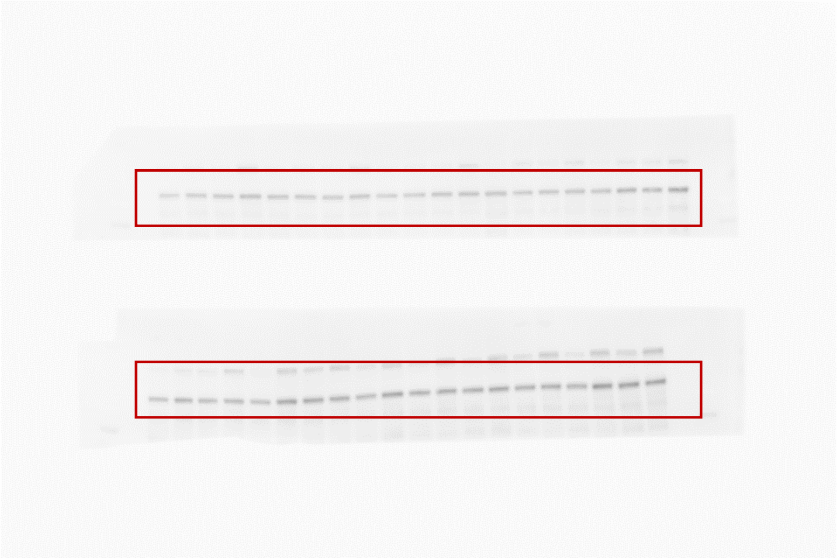

β-actin as loading control to pIGF1-R

Frontal cortex

membrane 1 and membrane 2

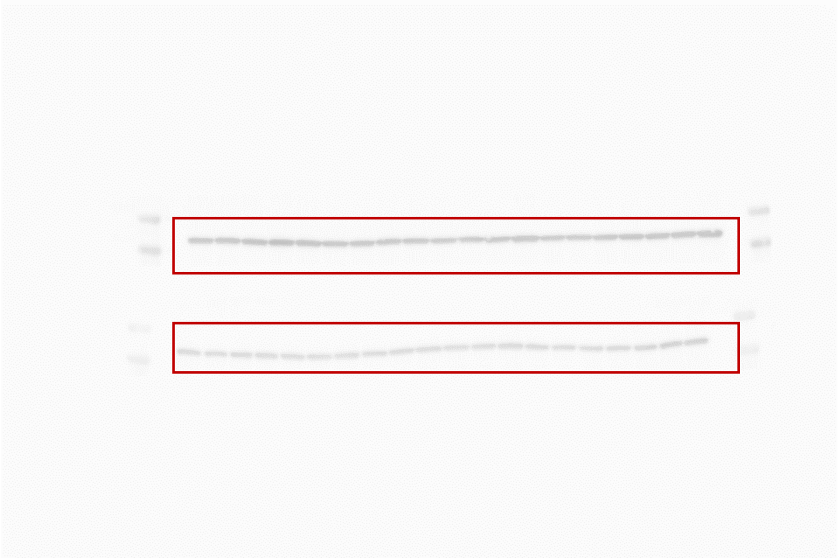

Samples from the left side - membrane 1:
CTRL, CTRL + STRESS, DEX, DEX + STRESS, CTRL, CTRL + STRESS, DEX, DEX + STRESS, CTRL, CTRL + STRESS, DEX, DEX + STRESS, CTRL, CTRL + STRESS, DEX, DEX + STRESS, CTRL, CTRL + STRESS, DEX, DEX + STRESS.

Samples from the left side - membrane 2:
CTRL, CTRL + STRESS, DEX, DEX + STRESS, CTRL, CTRL + STRESS, DEX, DEX + STRESS, CTRL, CTRL + STRESS, DEX, DEX + STRESS, CTRL, CTRL + STRESS, DEX, DEX + STRESS, CTRL, CTRL + STRESS, DEX, DEX + STRESS.

The results of the experiment are shown in Figure 6.

pIGF1-R

Hippocampus

membrane 1 and membrane 2

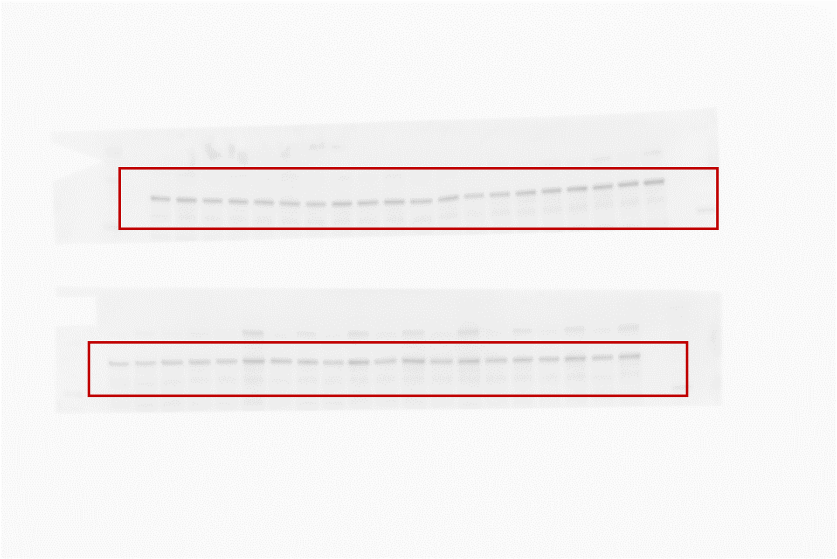

β-actin as loading control to pIGF1-R

Hippocampus

membrane 1 and membrane 2

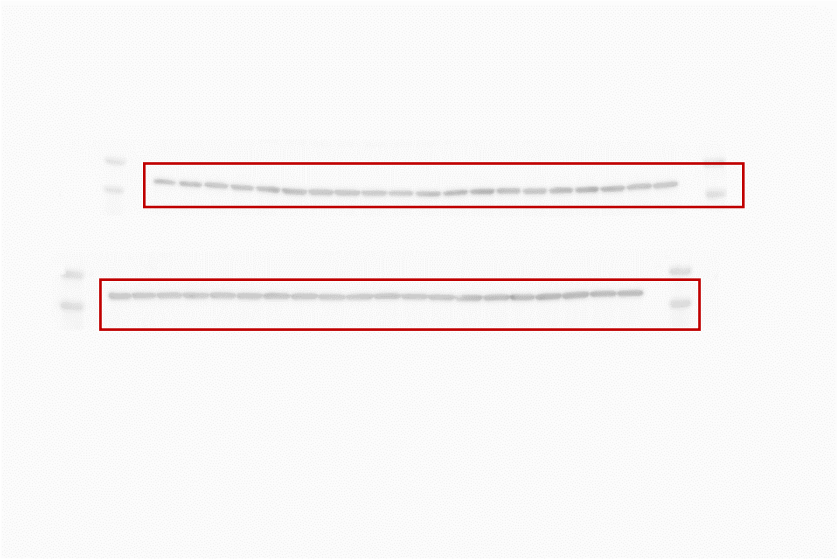

Samples from the left side - membrane 1:
CTRL, CTRL + STRESS, DEX, DEX + STRESS, CTRL, CTRL + STRESS, DEX, DEX + STRESS, CTRL, CTRL + STRESS, DEX, DEX + STRESS, CTRL, CTRL + STRESS, DEX, DEX + STRESS, CTRL, CTRL + STRESS, DEX, DEX + STRESS.

Samples from the left side - membrane 2:
CTRL, CTRL + STRESS, DEX, DEX + STRESS, CTRL, CTRL + STRESS, DEX, DEX + STRESS, CTRL, CTRL + STRESS, DEX, DEX + STRESS, CTRL, CTRL + STRESS, DEX, DEX + STRESS, CTRL, CTRL + STRESS, DEX, DEX + STRESS.

The results of the experiment are shown in Figure 6.

IGF1-R

Frontal cortex

membrane 1 and membrane 2

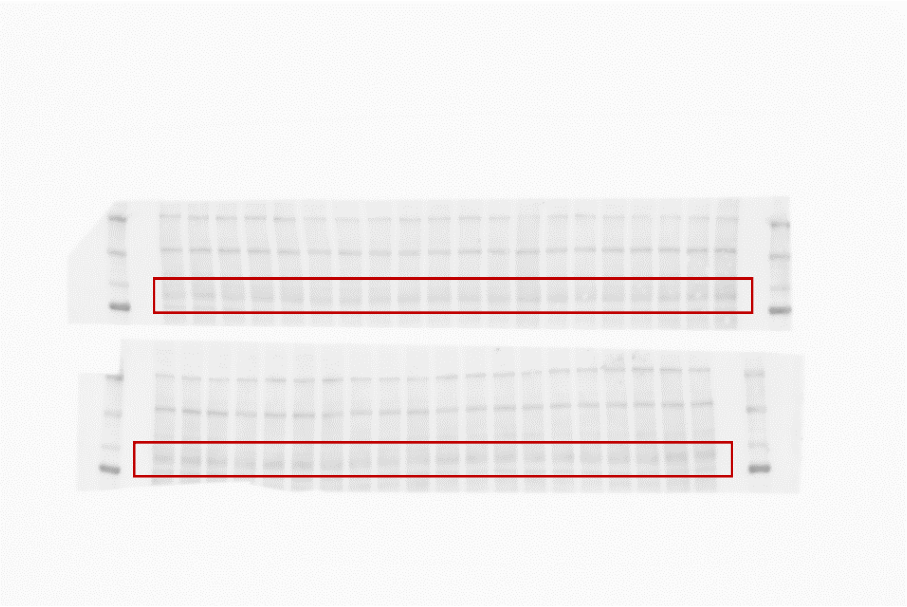

Samples from the left side - membrane 1:
CTRL, CTRL + STRESS, DEX, DEX + STRESS, CTRL, CTRL + STRESS, DEX, DEX + STRESS, CTRL, CTRL + STRESS, DEX, DEX + STRESS, CTRL, CTRL + STRESS, DEX, DEX + STRESS, CTRL, CTRL + STRESS, DEX, DEX + STRESS, CTRL, CTRL + STRESS, DEX, DEX + STRESS.

Samples from the left side - membrane 2:
CTRL, CTRL + STRESS, DEX, DEX + STRESS, CTRL, CTRL + STRESS, DEX, DEX + STRESS, CTRL, CTRL + STRESS, DEX, DEX + STRESS, CTRL, CTRL + STRESS, DEX, DEX + STRESS, CTRL, CTRL + STRESS, DEX, DEX + STRESS.

The results of the experiment are shown in Figure 6.

β-actin as loading control to IGF1-R

Frontal cortex

membrane 1 and membrane 2

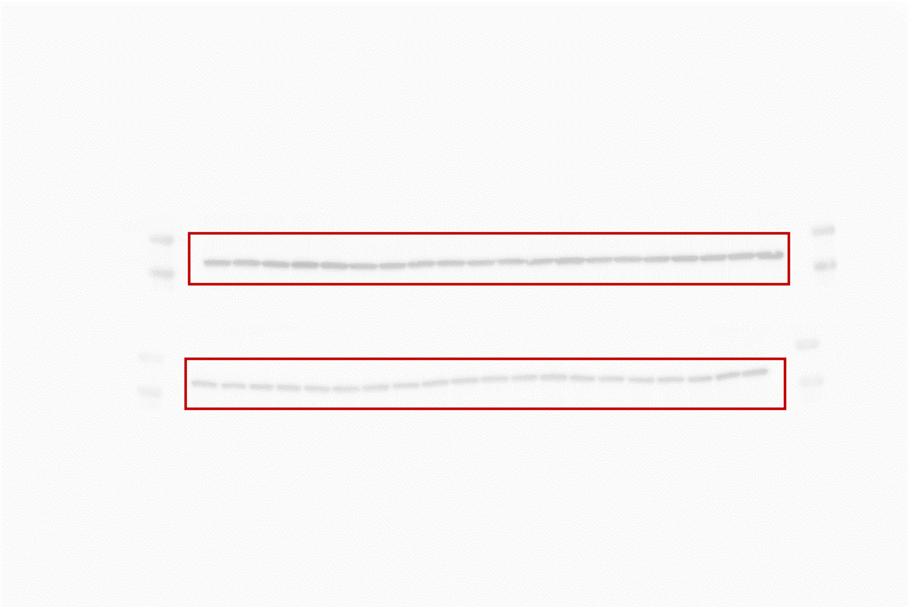

IGF1-R

Hippocampus

membrane 1 and membrane 2

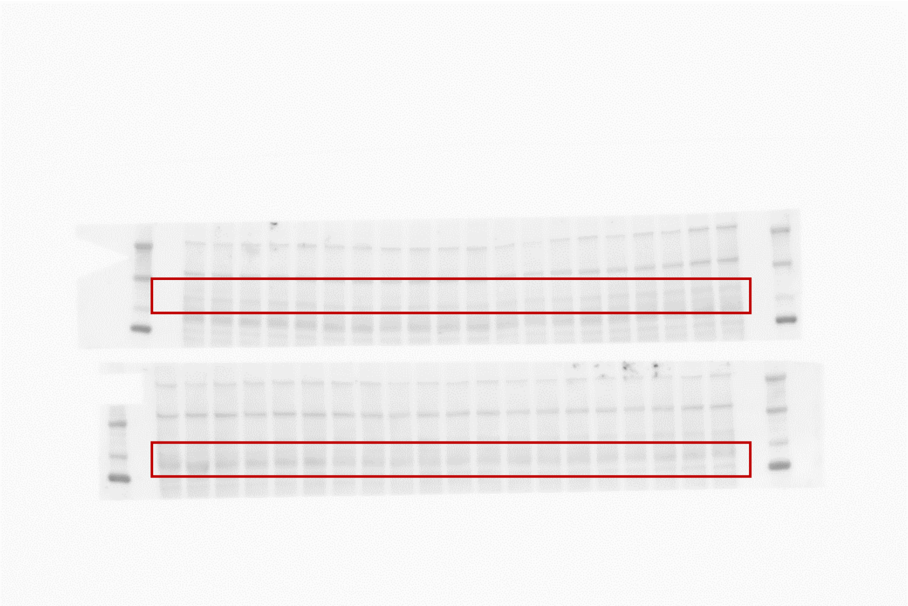

β-actin as loading control to IGF1-R

Hippocampus

membrane 1 and membrane 2

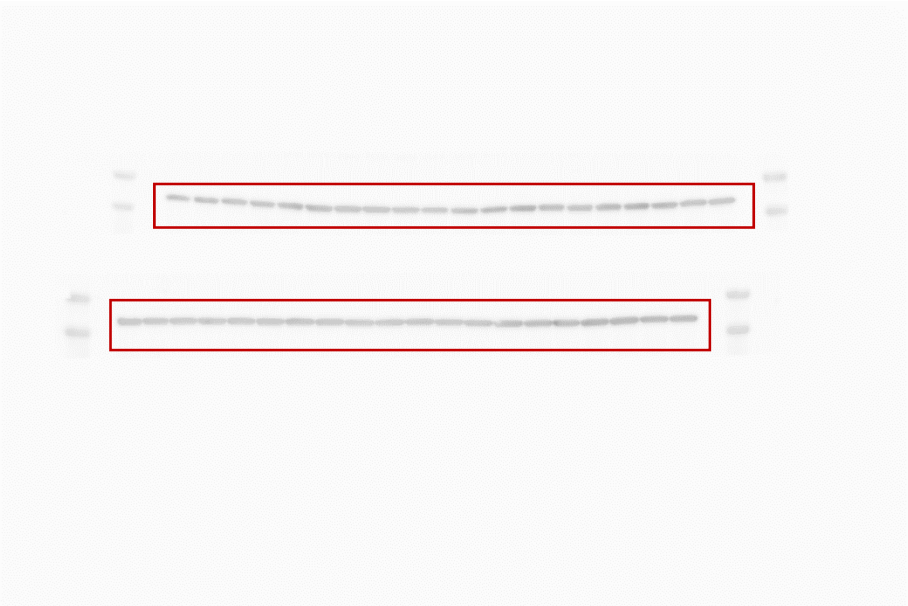

Samples from the left side - membrane 1:
CTRL, CTRL + STRESS, DEX, DEX + STRESS, CTRL, CTRL + STRESS, DEX, DEX + STRESS, CTRL, CTRL + STRESS, DEX, DEX + STRESS, CTRL, CTRL + STRESS, DEX, DEX + STRESS, CTRL, CTRL + STRESS, DEX, DEX + STRESS.

Samples from the left side - membrane 2:
CTRL, CTRL + STRESS, DEX, DEX + STRESS, CTRL, CTRL + STRESS, DEX, DEX + STRESS, CTRL, CTRL + STRESS, DEX, DEX + STRESS, CTRL, CTRL + STRESS, DEX, DEX + STRESS, CTRL, CTRL + STRESS, DEX, DEX + STRESS.

The results of the experiment are shown in Figure 6.

pCREB

Frontal cortex

membrane 1 and membrane 2

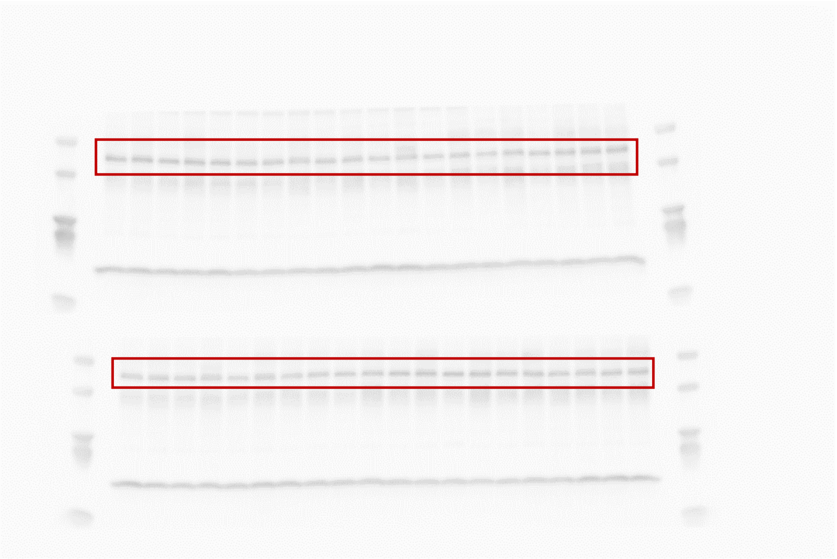

CREB

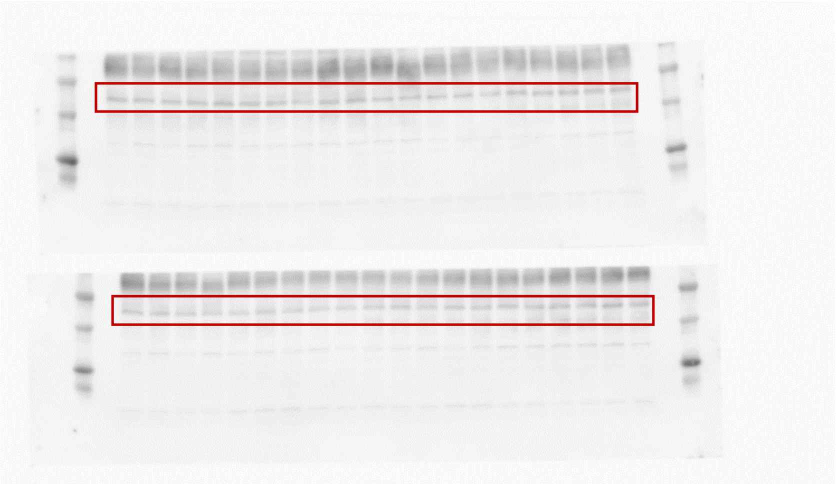

Vinculin as loading control to pCREB i CREB

Frontal cortex

membrane 1 and membrane 2

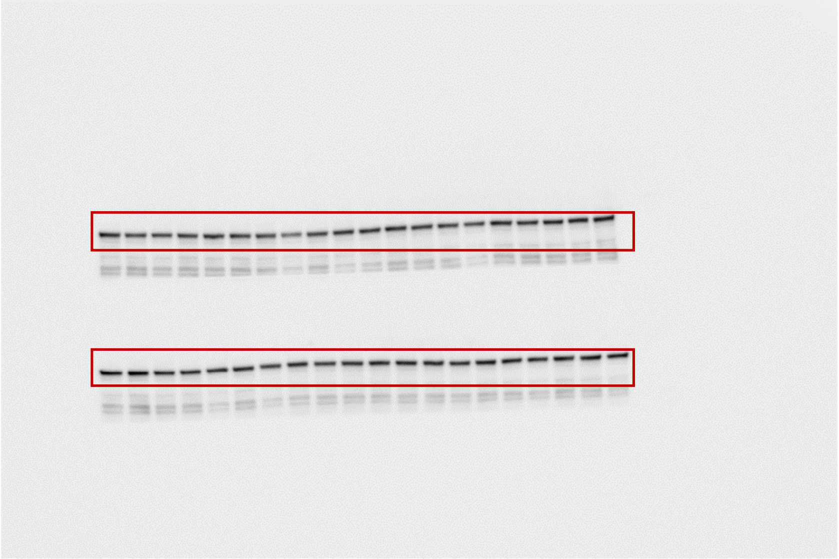

Samples from the left side - membrane 1:
CTRL, CTRL + STRESS, DEX, DEX + STRESS, CTRL, CTRL + STRESS, DEX, DEX + STRESS, CTRL, CTRL + STRESS, DEX, DEX + STRESS, CTRL, CTRL + STRESS, DEX, DEX + STRESS, CTRL, CTRL + STRESS, DEX, DEX + STRESS.

Samples from the left side - membrane 2:
CTRL, CTRL + STRESS, DEX, DEX + STRESS, CTRL, CTRL + STRESS, DEX, DEX + STRESS, CTRL, CTRL + STRESS, DEX, DEX + STRESS, CTRL, CTRL + STRESS, DEX, DEX + STRESS, CTRL, CTRL + STRESS, DEX, DEX + STRESS.

The results of the experiment are shown in Figure 7.

pCREB

Hippocampus

membrane 1 and membrane 2

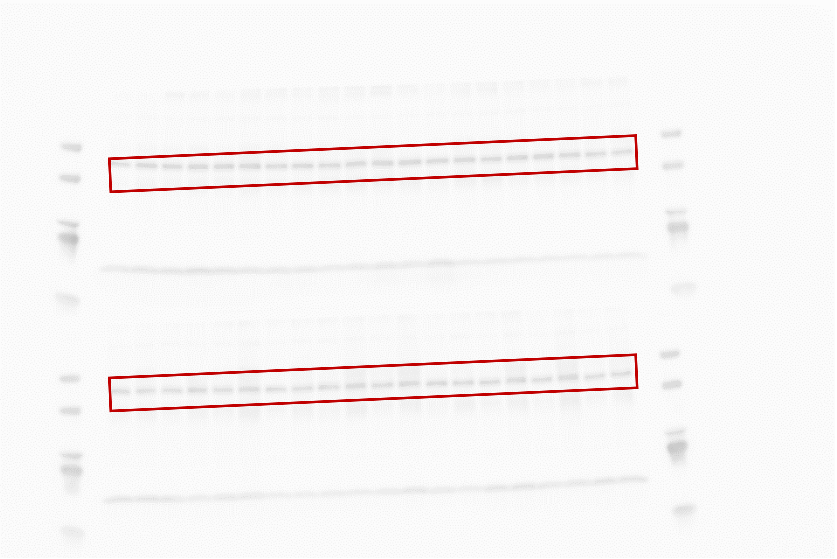

CREB

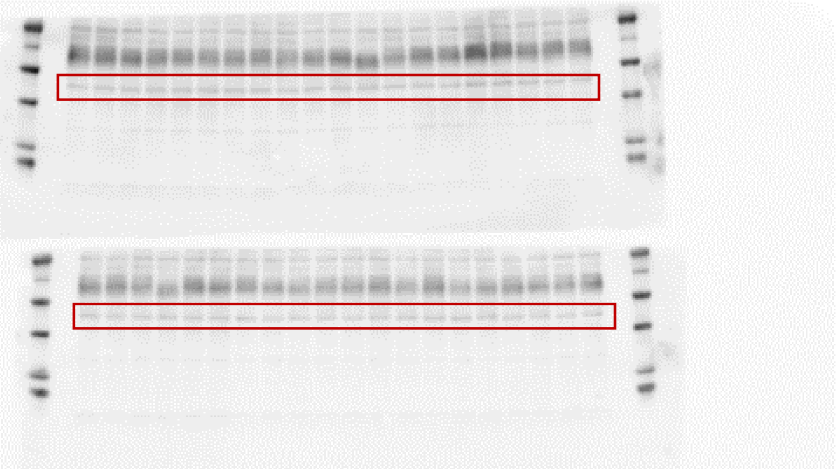

Vinculin as loading control to pCREB i CREB

Hippocampus

membrane 1 and membrane 2

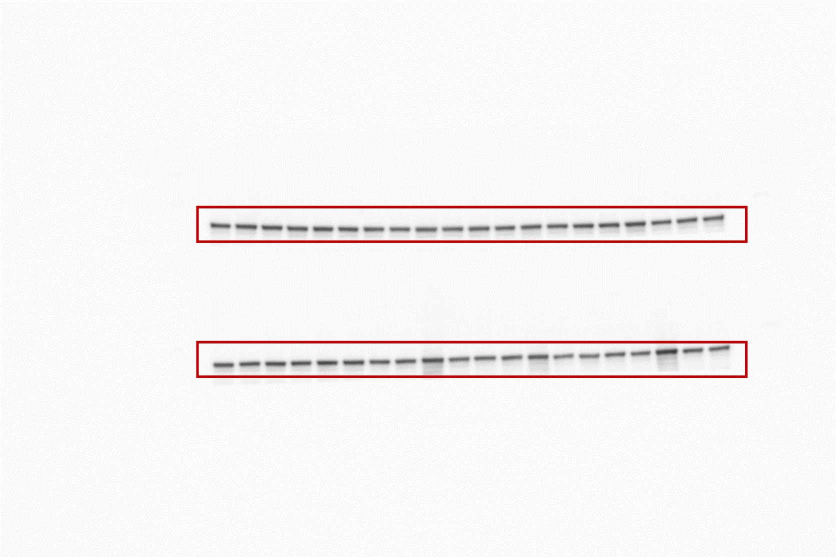

Samples from the left side - membrane 1:
CTRL, CTRL + STRESS, DEX, DEX + STRESS, CTRL, CTRL + STRESS, DEX, DEX + STRESS, CTRL, CTRL + STRESS, DEX, DEX + STRESS, CTRL, CTRL + STRESS, DEX, DEX + STRESS, CTRL, CTRL + STRESS, DEX, DEX + STRESS.

Samples from the left side - membrane 2:
CTRL, CTRL + STRESS, DEX, DEX + STRESS, CTRL, CTRL + STRESS, DEX, DEX + STRESS, CTRL, CTRL + STRESS, DEX, DEX + STRESS, CTRL, CTRL + STRESS, DEX, DEX + STRESS, CTRL, CTRL + STRESS, DEX, DEX + STRESS.

The results of the experiment are shown in Figure 7.

GR

Frontal cortex

membrane 1 and membrane 2

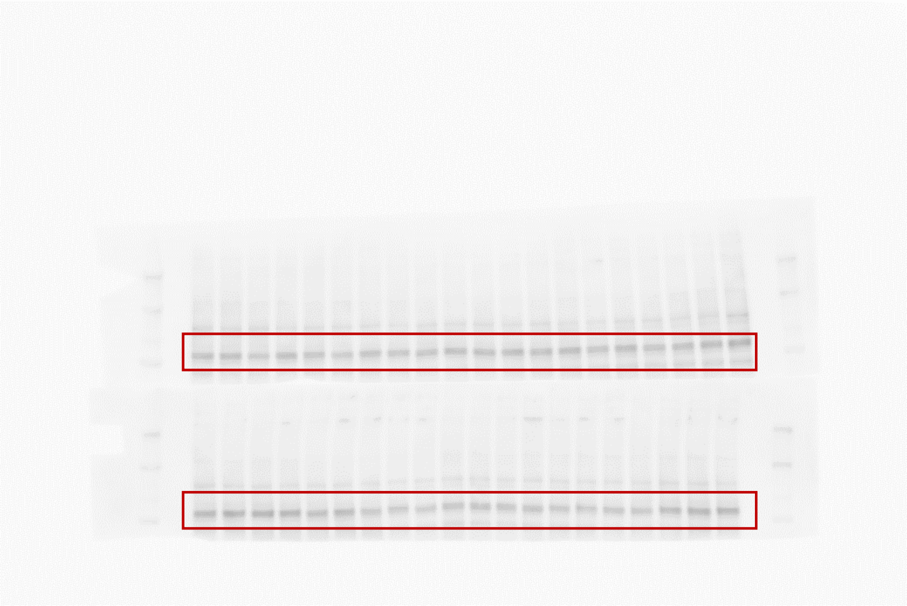

Samples from the left side - membrane 1:
CTRL, CTRL + STRESS, DEX, DEX + STRESS, CTRL, CTRL + STRESS, DEX, DEX + STRESS, CTRL, CTRL + STRESS, DEX, DEX + STRESS, CTRL, CTRL + STRESS, DEX, DEX + STRESS, CTRL, CTRL + STRESS, DEX, DEX + STRESS.

Samples from the left side - membrane 2:
CTRL, CTRL + STRESS, DEX, DEX + STRESS, CTRL, CTRL + STRESS, DEX, DEX + STRESS, CTRL, CTRL + STRESS, DEX, DEX + STRESS, CTRL, CTRL + STRESS, DEX, DEX + STRESS, CTRL, CTRL + STRESS, DEX, DEX + STRESS.

The results of the experiment are shown in Figure 8.

β-actin as loading control to GR

Frontal cortex

membrane 1 and membrane 2

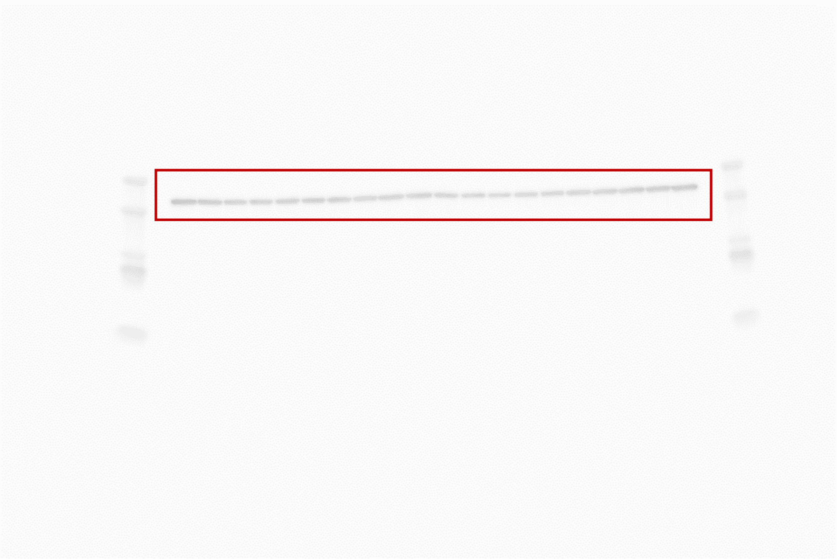

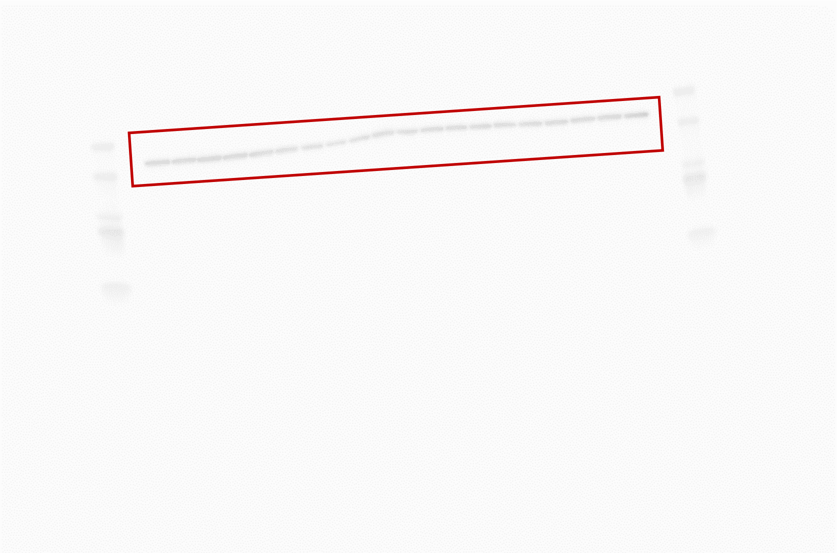

GR

Hippocampus

membrane 1 and membrane 2

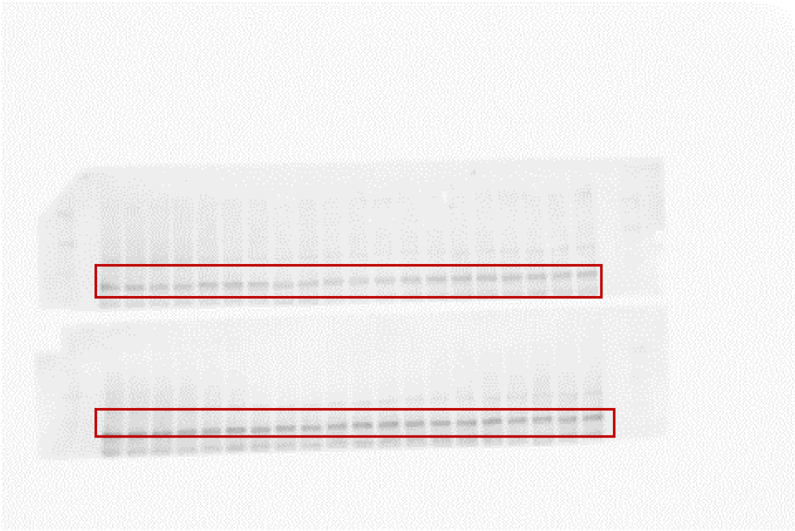

Samples from the left side - membrane 1:
CTRL, CTRL + STRESS, DEX, DEX + STRESS, CTRL, CTRL + STRESS, DEX, DEX + STRESS, CTRL, CTRL + STRESS, DEX, DEX + STRESS, CTRL, CTRL + STRESS, DEX, DEX + STRESS, CTRL, CTRL + STRESS, DEX, DEX + STRESS, CTRL, CTRL + STRESS, DEX, DEX + STRESS.

Samples from the left side - membrane 2:
CTRL, CTRL + STRESS, DEX, DEX + STRESS, CTRL, CTRL + STRESS, DEX, DEX + STRESS, CTRL, CTRL + STRESS, DEX, DEX + STRESS, CTRL, CTRL + STRESS, DEX, DEX + STRESS, CTRL, CTRL + STRESS, DEX, DEX + STRESS.

The results of the experiment are shown in Figure 8.

β-actin as loading control to GR

Hippocampus

membrane 1 and membrane 2

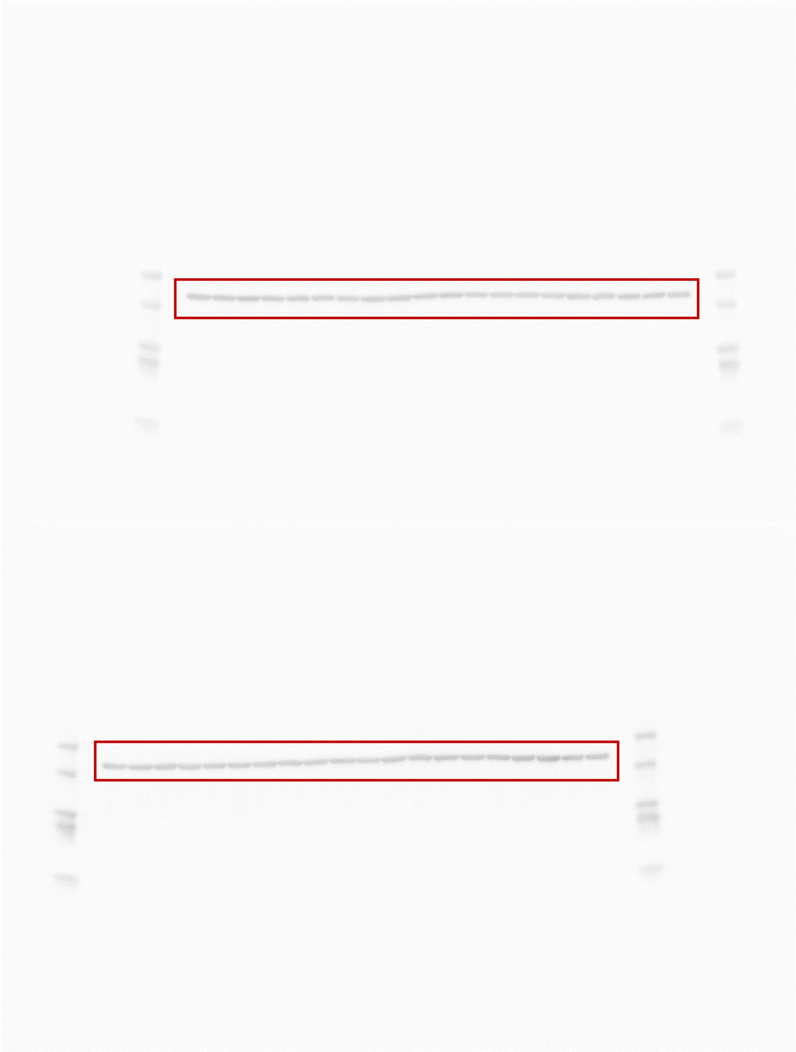

MR

Frontal cortex

membrane 1 and membrane 2

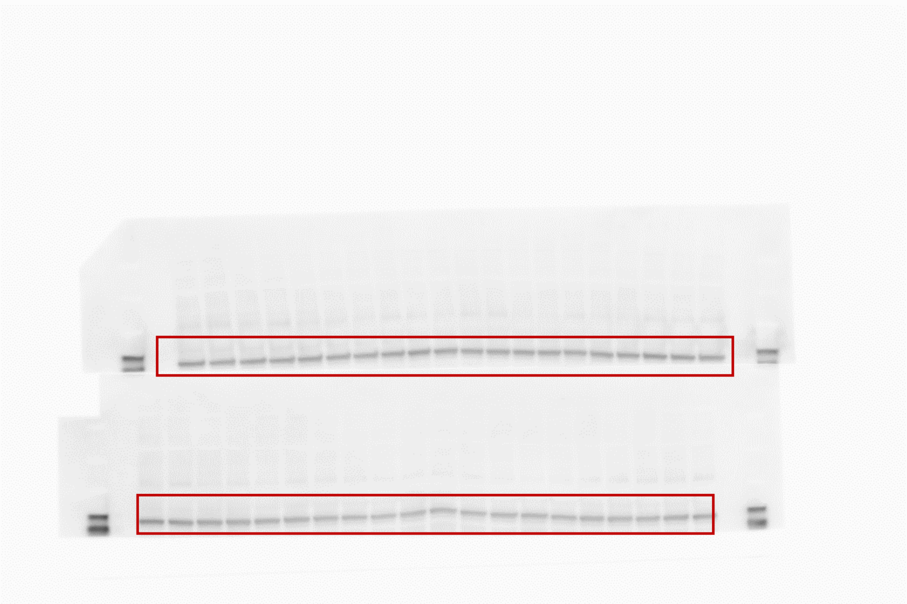

Samples from the left side - membrane 1:
CTRL, CTRL + STRESS, DEX, DEX + STRESS, CTRL, CTRL + STRESS, DEX, DEX + STRESS, CTRL, CTRL + STRESS, DEX, DEX + STRESS, CTRL, CTRL + STRESS, DEX, DEX + STRESS, CTRL, CTRL + STRESS, DEX, DEX + STRESS.

Samples from the left side - membrane 2:
CTRL, CTRL + STRESS, DEX, DEX + STRESS, CTRL, CTRL + STRESS, DEX, DEX + STRESS, CTRL, CTRL + STRESS, DEX, DEX + STRESS, CTRL, CTRL + STRESS, DEX, DEX + STRESS, CTRL, CTRL + STRESS, DEX, DEX + STRESS.

The results of the experiment are shown in Figure 8.

β-actin as loading control to MR

Frontal cortex

membrane 1 and membrane 2

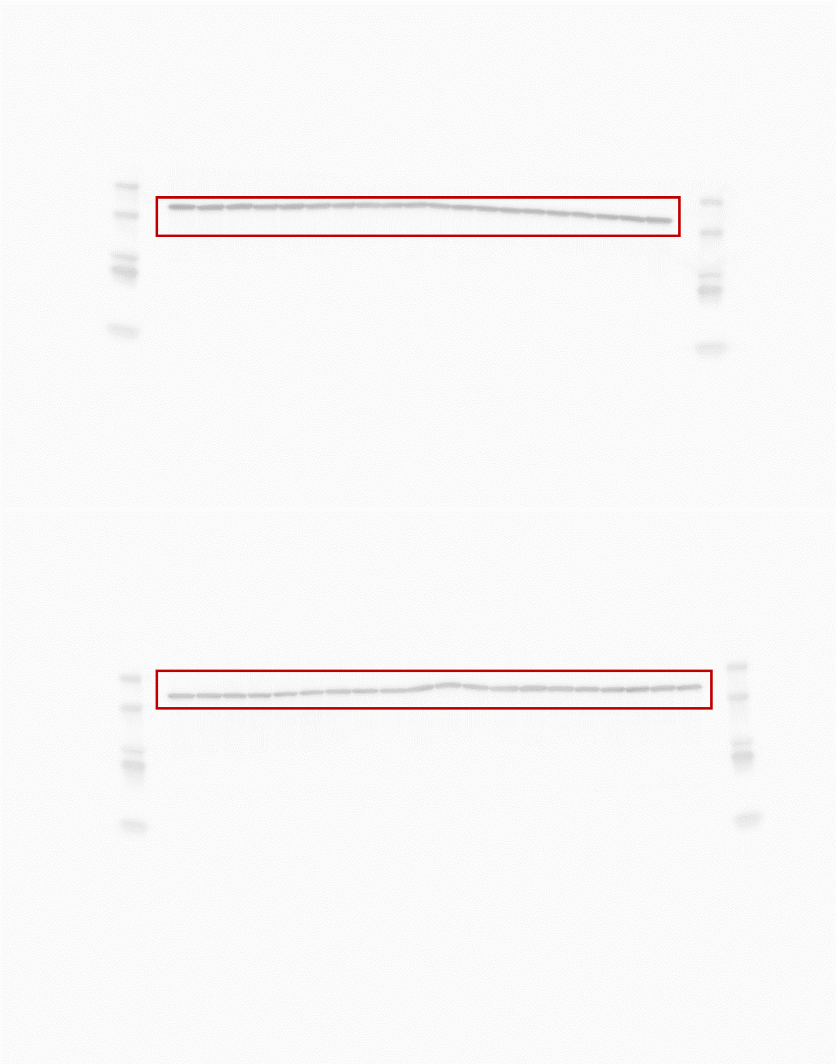

MR

Hippocampus

membrane 1 and membrane 2

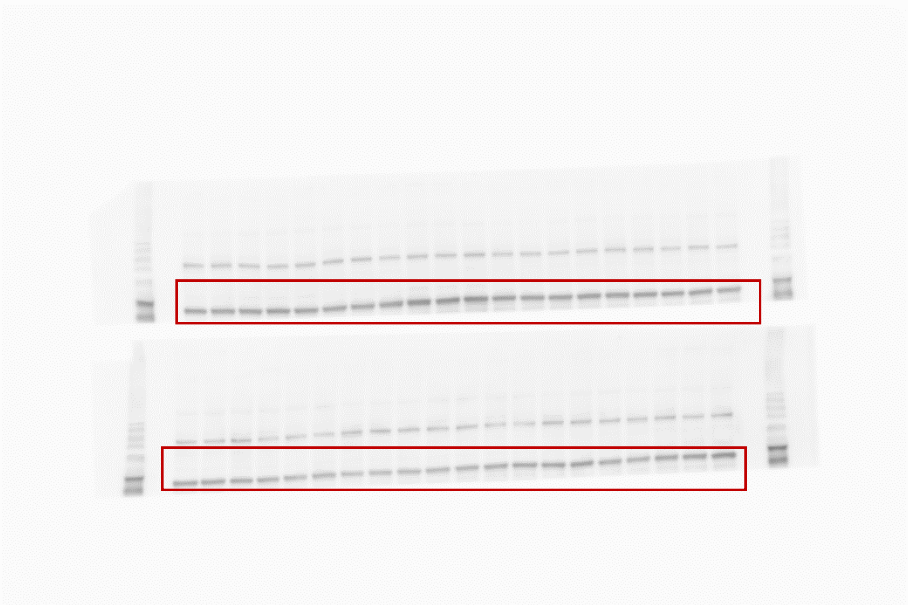

Samples from the left side - membrane 1:
CTRL, CTRL + STRESS, DEX, DEX + STRESS, CTRL, CTRL + STRESS, DEX, DEX + STRESS, CTRL, CTRL + STRESS, DEX, DEX + STRESS, CTRL, CTRL + STRESS, DEX, DEX + STRESS, CTRL, CTRL + STRESS, DEX, DEX + STRESS, CTRL, CTRL + STRESS, DEX, DEX + STRESS.

Samples from the left side - membrane 2:
CTRL, CTRL + STRESS, DEX, DEX + STRESS, CTRL, CTRL + STRESS, DEX, DEX + STRESS, CTRL, CTRL + STRESS, DEX, DEX + STRESS, CTRL, CTRL + STRESS, DEX, DEX + STRESS, CTRL, CTRL + STRESS, DEX, DEX + STRESS.

The results of the experiment are shown in Figure 8.

β-actin as loading control to MR

Hippocampus

membrane 1 and membrane 2

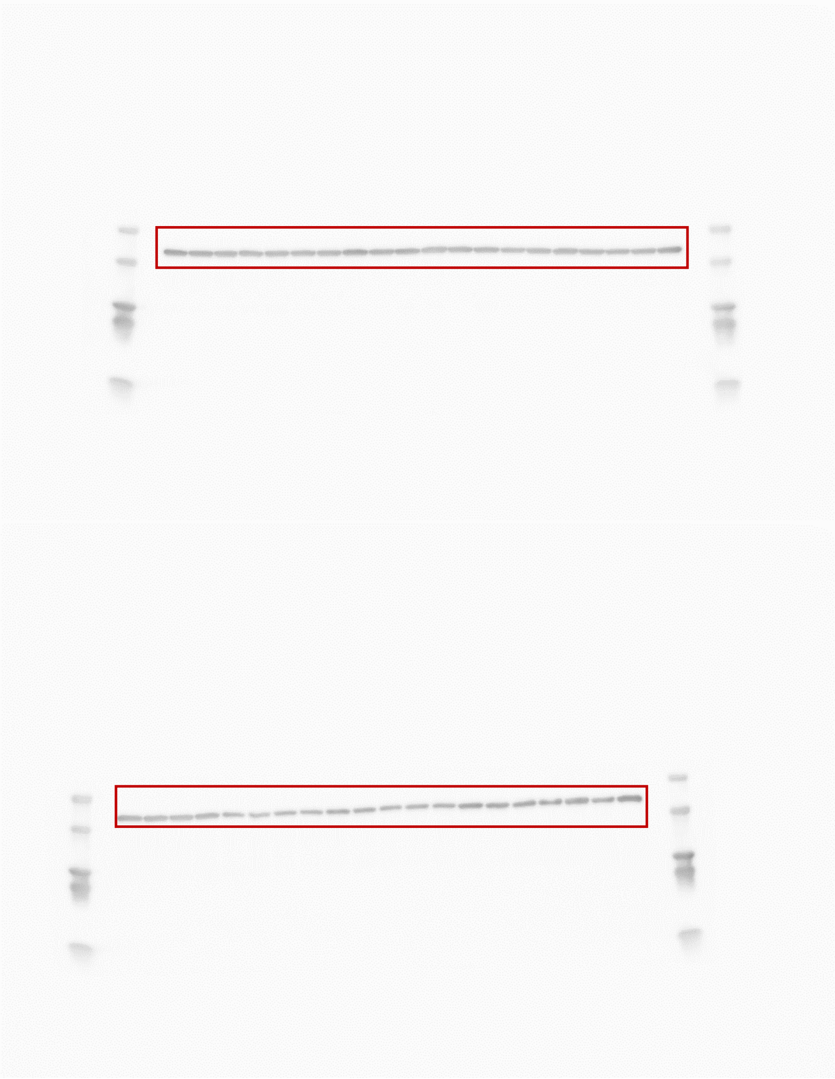

SGK1

Frontal cortex

membrane 1 and membrane 2

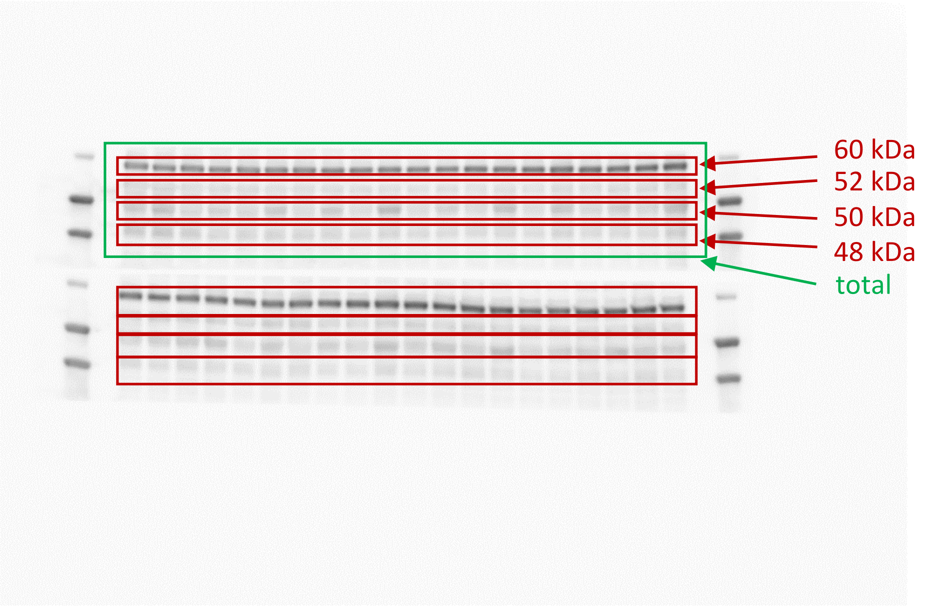

Vinculin as loading control to SGK1

Frontal cortex

membrane 1 and membrane 2

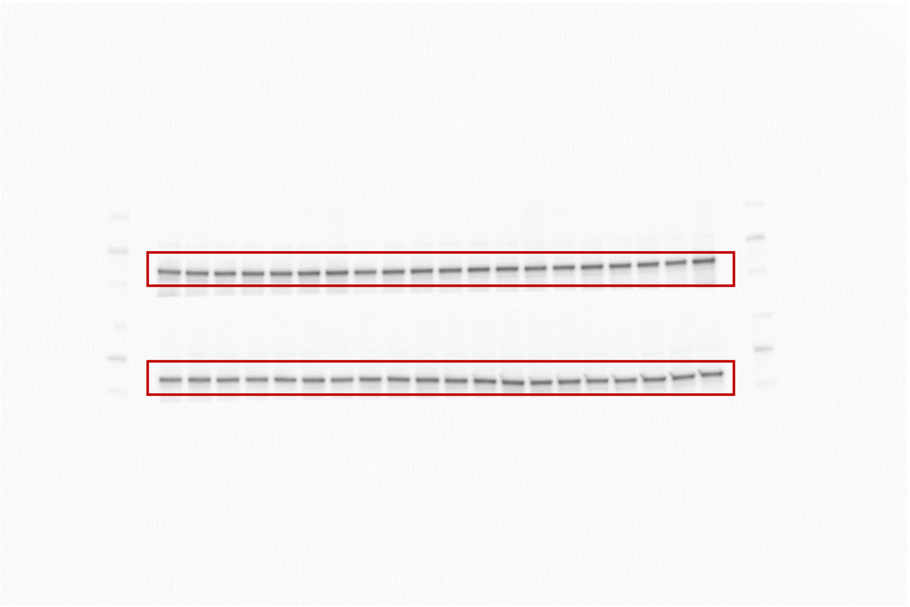

Samples from the left side - membrane 1:
CTRL, CTRL + STRESS, DEX, DEX + STRESS, CTRL, CTRL + STRESS, DEX, DEX + STRESS, CTRL, CTRL + STRESS, DEX, DEX + STRESS, CTRL, CTRL + STRESS, DEX, DEX + STRESS, CTRL, CTRL + STRESS, DEX, DEX + STRESS.

Samples from the left side - membrane 2:
CTRL, CTRL + STRESS, DEX, DEX + STRESS, CTRL, CTRL + STRESS, DEX, DEX + STRESS, CTRL, CTRL + STRESS, DEX, DEX + STRESS, CTRL, CTRL + STRESS, DEX, DEX + STRESS, CTRL, CTRL + STRESS, DEX, DEX + STRESS.

The results of the experiment are shown in Figure 9**.**

SGK1

Hippocampus

membrane 1 and membrane 2

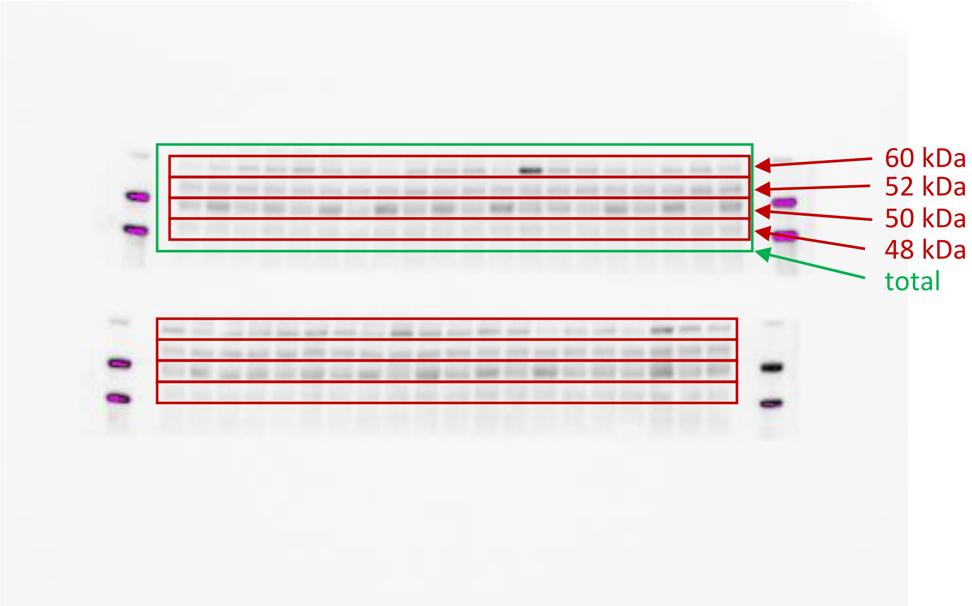

Samples from the left side - membrane 1:
CTRL, CTRL + STRESS, DEX, DEX + STRESS, CTRL, CTRL + STRESS, DEX, DEX + STRESS, CTRL, CTRL + STRESS, DEX, DEX + STRESS, CTRL, CTRL + STRESS, DEX, DEX + STRESS, CTRL, CTRL + STRESS, DEX, DEX + STRESS.

Samples from the left side - membrane 2:
CTRL, CTRL + STRESS, DEX, DEX + STRESS, CTRL, CTRL + STRESS, DEX, DEX + STRESS, CTRL, CTRL + STRESS, DEX, DEX + STRESS, CTRL, CTRL + STRESS, DEX, DEX + STRESS, CTRL, CTRL + STRESS, DEX, DEX + STRESS.

The results of the experiment are shown in Figure 9.

Vinculin as loading control to SGK1

Hippocampus

membrane 1 and membrane 2

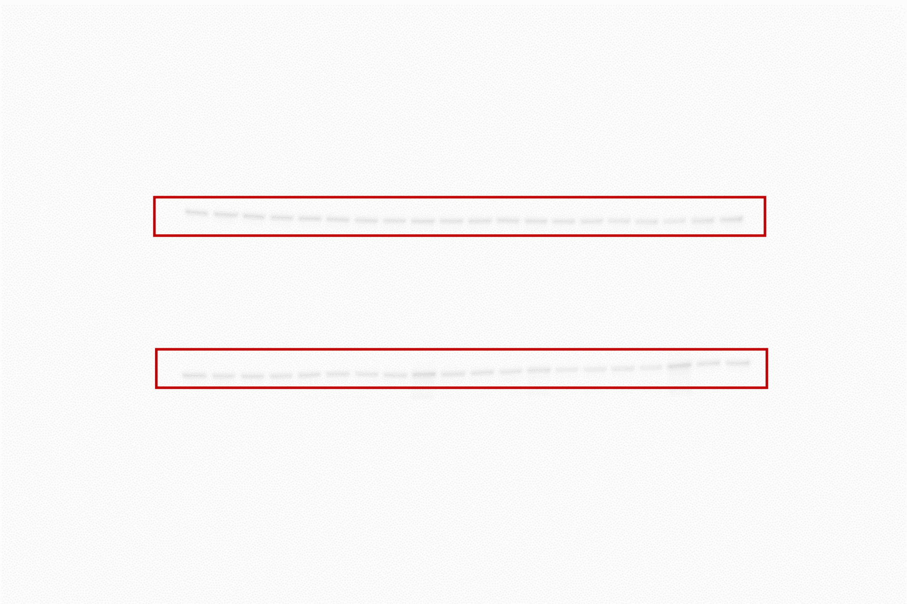

GILZ

Frontal cortex

membrane 1 and membrane 2

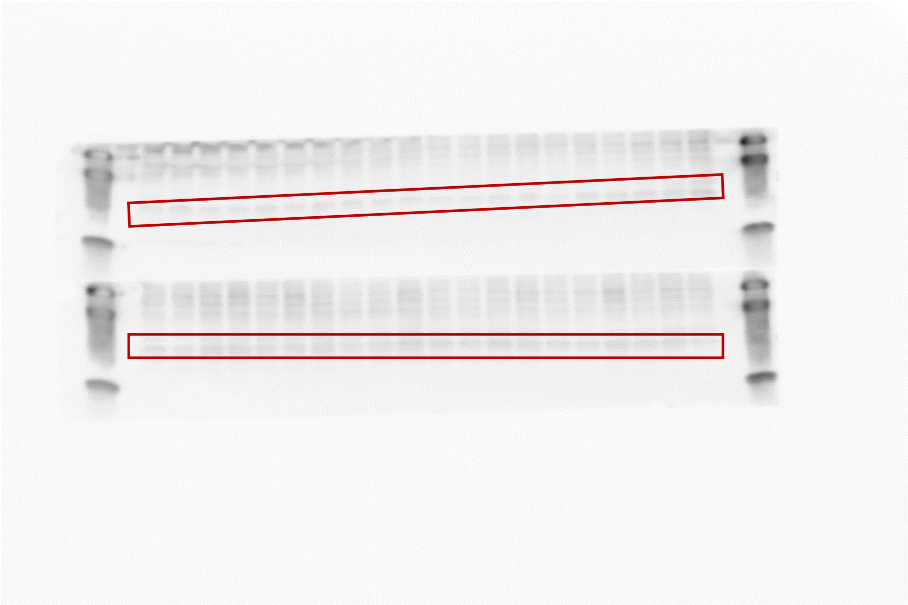

Vinculin as loading control to GILZ

Frontal cortex

membrane 1 and membrane 2

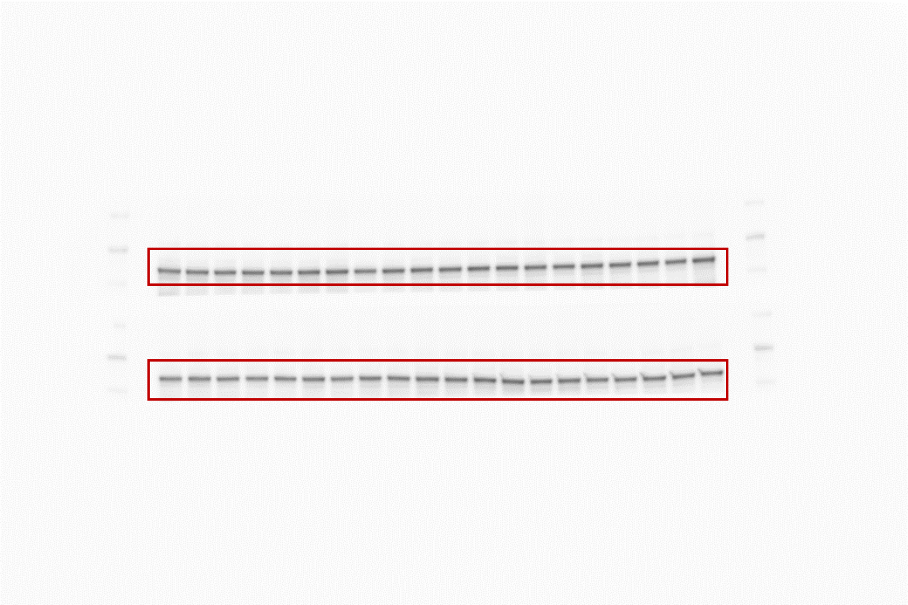

Samples from the left side - membrane 1:
CTRL, CTRL + STRESS, DEX, DEX + STRESS, CTRL, CTRL + STRESS, DEX, DEX + STRESS, CTRL, CTRL + STRESS, DEX, DEX + STRESS, CTRL, CTRL + STRESS, DEX, DEX + STRESS, CTRL, CTRL + STRESS, DEX, DEX + STRESS.

Samples from the left side - membrane 2:
CTRL, CTRL + STRESS, DEX, DEX + STRESS, CTRL, CTRL + STRESS, DEX, DEX + STRESS, CTRL, CTRL + STRESS, DEX, DEX + STRESS, CTRL, CTRL + STRESS, DEX, DEX + STRESS, CTRL, CTRL + STRESS, DEX, DEX + STRESS.

The results of the experiment are shown in Figure 9.

GILZ

Hippocampus

membrane 1 and membrane 2

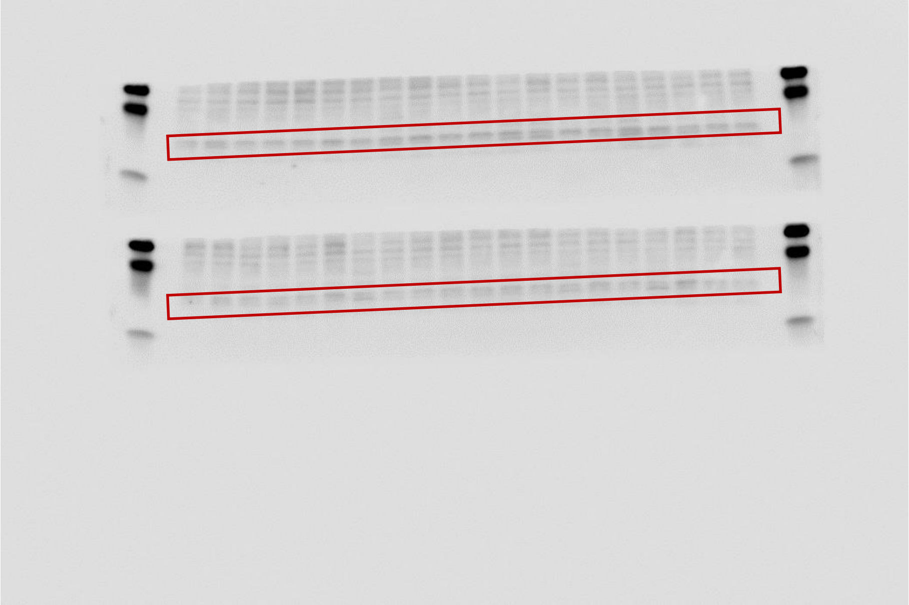

Vinculin as loading control to GILZ

Hippocampus

membrane 1 and membrane 2

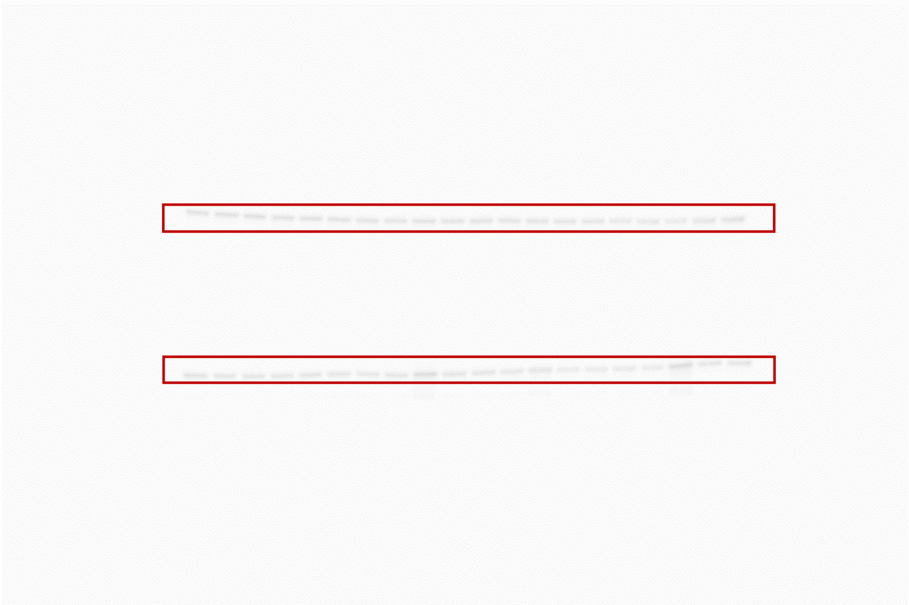

Samples from the left side - membrane 1:
CTRL, CTRL + STRESS, DEX, DEX + STRESS, CTRL, CTRL + STRESS, DEX, DEX + STRESS, CTRL, CTRL + STRESS, DEX, DEX + STRESS, CTRL, CTRL + STRESS, DEX, DEX + STRESS, CTRL, CTRL + STRESS, DEX, DEX + STRESS.

Samples from the left side - membrane 2:
CTRL, CTRL + STRESS, DEX, DEX + STRESS, CTRL, CTRL + STRESS, DEX, DEX + STRESS, CTRL, CTRL + STRESS, DEX, DEX + STRESS, CTRL, CTRL + STRESS, DEX, DEX + STRESS, CTRL, CTRL + STRESS, DEX, DEX + STRESS.

The results of the experiment are shown in Figure 9.

MeCP2

Frontal cortex (nuclear fraction)

membrane 1 and membrane 2

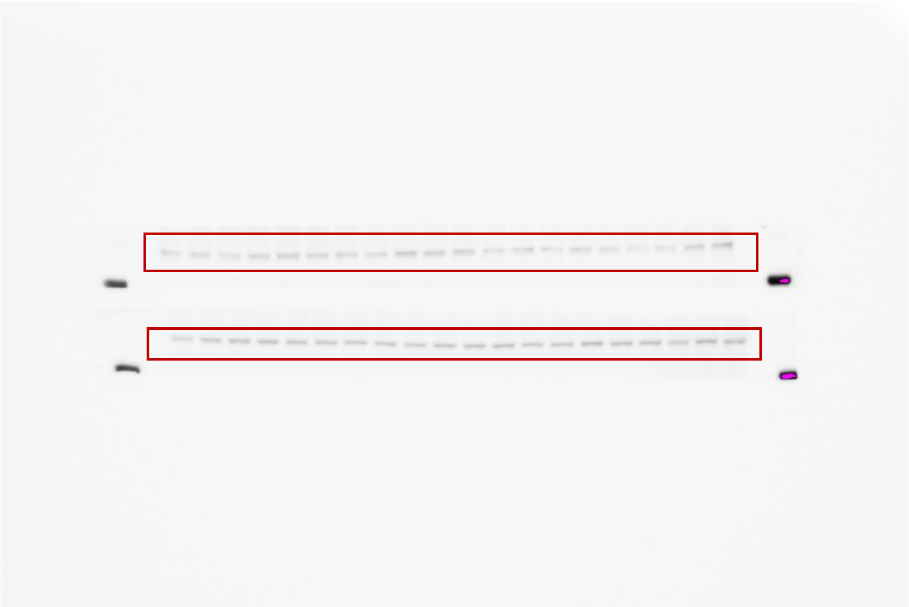

Samples from the left side - membrane 1:
CTRL, DEX, CTRL + STRESS, DEX + STRESS, CTRL, DEX, CTRL + STRESS, DEX + STRESS, CTRL, DEX, CTRL + STRESS, DEX + STRESS, CTRL, DEX, CTRL + STRESS, DEX + STRESS, CTRL, DEX, CTRL + STRESS, DEX + STRESS.

Samples from the left side - membrane 2:
CTRL, DEX, CTRL + STRESS, DEX + STRESS, CTRL, DEX, CTRL + STRESS, DEX + STRESS, CTRL, DEX, CTRL + STRESS, DEX + STRESS, CTRL, DEX, CTRL + STRESS, DEX + STRESS, CTRL, DEX, CTRL + STRESS, DEX + STRESS.

The results of the experiment are shown in Figure 10.

β-actin as loading control to MeCP2

Frontal cortex (nuclear fraction)

membrane 1 and membrane 2

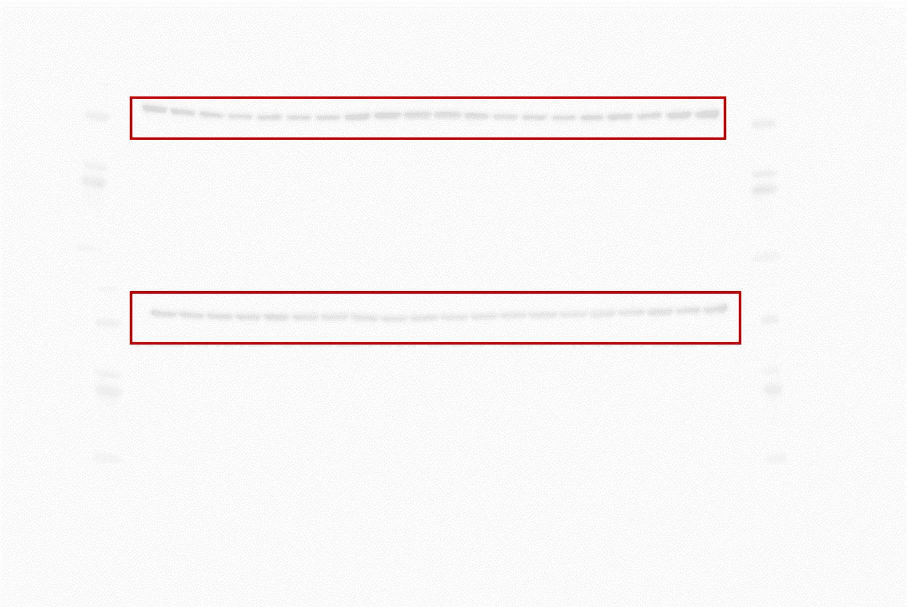

MeCP2

Hippocampus (nuclear fraction)

membrane 1 and membrane 2

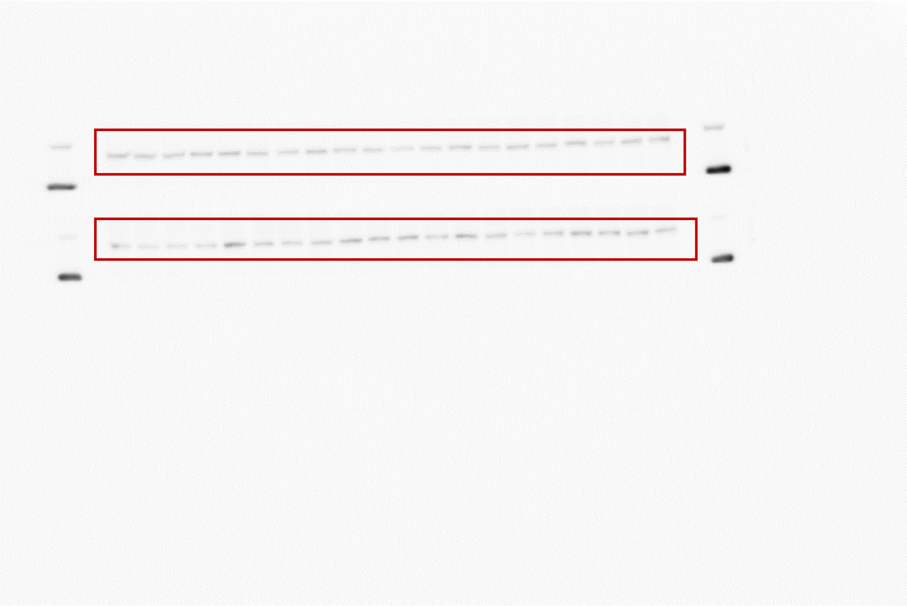

Samples from the left side - membrane 1:
CTRL, DEX, CTRL + STRESS, DEX + STRESS, CTRL, DEX, CTRL + STRESS, DEX + STRESS, CTRL, DEX, CTRL + STRESS, DEX + STRESS, CTRL, DEX, CTRL + STRESS, DEX + STRESS, CTRL, DEX, CTRL + STRESS, DEX + STRESS

Samples from the left side - membrane 2:
CTRL, DEX, CTRL + STRESS, DEX + STRESS, CTRL, DEX, CTRL + STRESS, DEX + STRESS, CTRL, DEX, CTRL + STRESS, DEX + STRESS, CTRL, DEX, CTRL + STRESS, DEX + STRESS, CTRL, DEX, CTRL + STRESS, DEX + STRESS

The results of the experiment are shown in Figure 10.

β-actin as loading control to MeCP2

Hippocampus (nuclear fraction)

membrane 1 and membrane 2

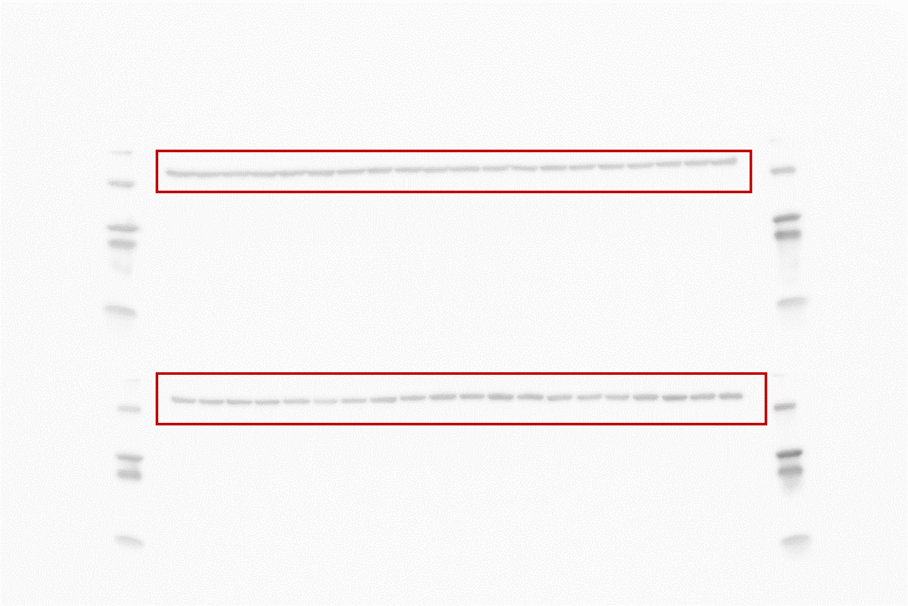

HDAC5

Frontal cortex (nuclear fraction)

membrane 1 and membrane 2

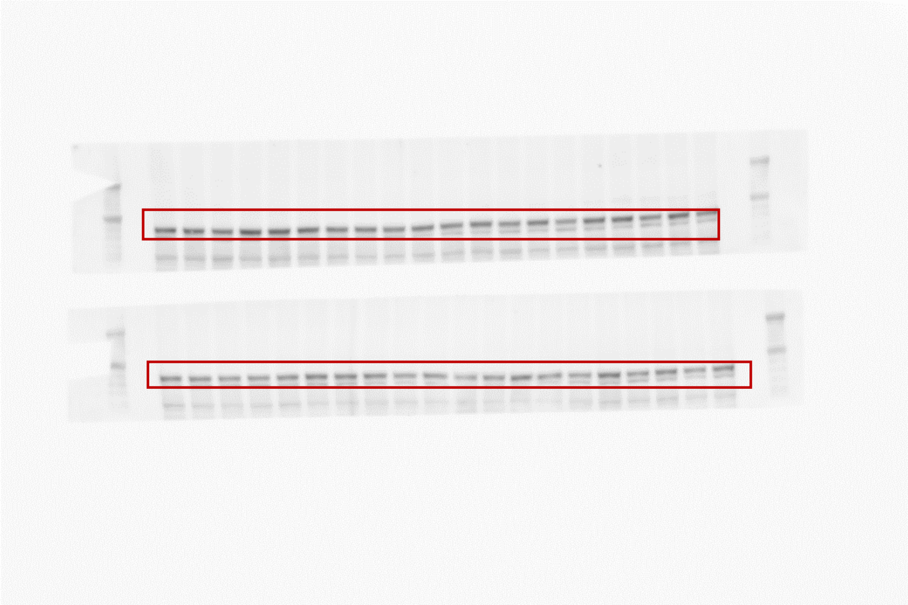

Samples from the left side - membrane 1:
CTRL, DEX, CTRL + STRESS, DEX + STRESS, CTRL, DEX, CTRL + STRESS, DEX + STRESS, CTRL, DEX, CTRL + STRESS, DEX + STRESS, CTRL, DEX, CTRL + STRESS, DEX + STRESS, CTRL, DEX, CTRL + STRESS, DEX + STRESS.

Samples from the left side - membrane 2:
CTRL, DEX, CTRL + STRESS, DEX + STRESS, CTRL, DEX, CTRL + STRESS, DEX + STRESS, CTRL, DEX, CTRL + STRESS, DEX + STRESS, CTRL, DEX, CTRL + STRESS, DEX + STRESS, CTRL, DEX, CTRL + STRESS, DEX + STRESS.

The results of the experiment are shown in Figure 10.

β-actin as loading control to HDAC5

Frontal cortex (nuclear fraction)

membrane 1 and membrane 2

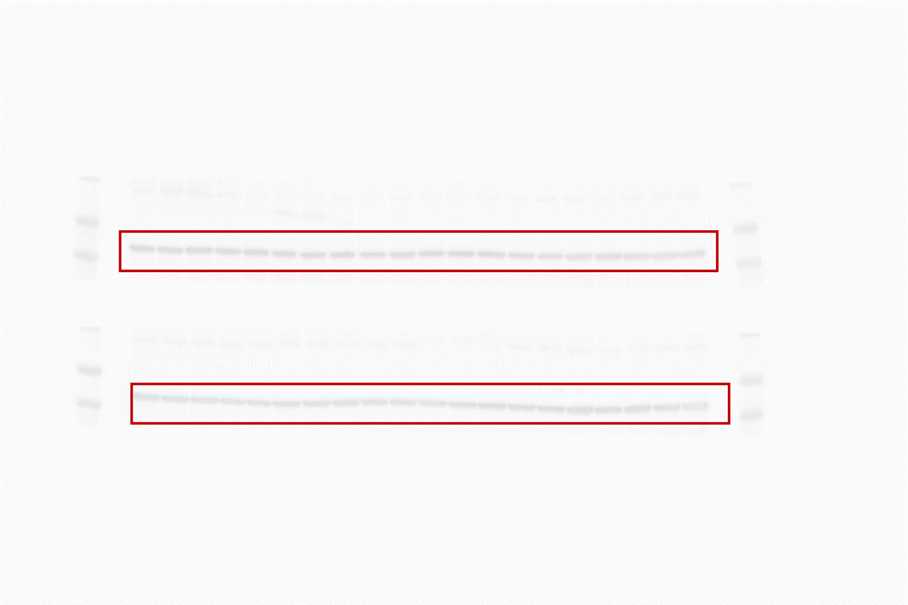

HDAC5

Hippocampus (nuclear fraction)

membrane 1 and membrane 2

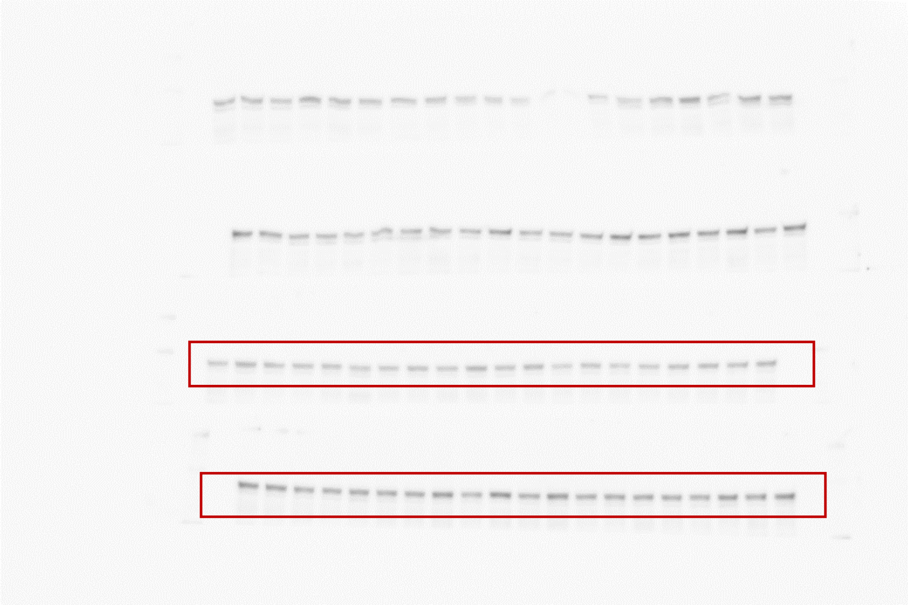

Samples from the left side - membrane 1:
CTRL, DEX, CTRL + STRESS, DEX + STRESS, CTRL, DEX, CTRL + STRESS, DEX + STRESS, CTRL, DEX, CTRL + STRESS, DEX + STRESS, CTRL, DEX, CTRL + STRESS, DEX + STRESS, CTRL, DEX, CTRL + STRESS, DEX + STRESS.

Samples from the left side - membrane 2:
CTRL, DEX, CTRL + STRESS, DEX + STRESS, CTRL, DEX, CTRL + STRESS, DEX + STRESS, CTRL, DEX, CTRL + STRESS, DEX + STRESS, CTRL, DEX, CTRL + STRESS, DEX + STRESS, CTRL, DEX, CTRL + STRESS, DEX + STRESS.

The results of the experiment are shown in Figure 10.

β-actin as loading control to HDAC5

Hippocampus (nuclear fraction)

membrane 1 and membrane 2

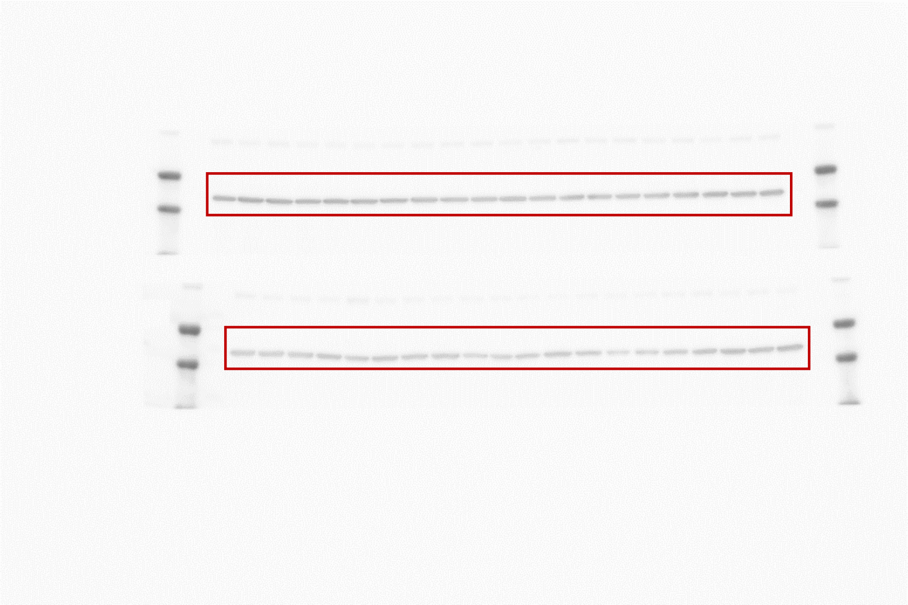

HDAC1

Frontal cortex (nuclear fraction)

membrane 1 and membrane 2

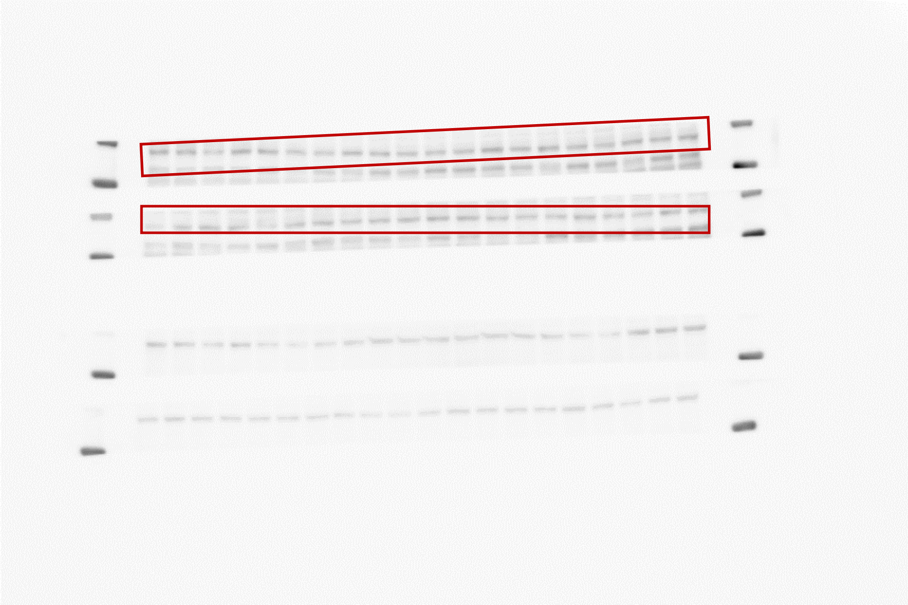

Samples from the left side - membrane 1:
CTRL, DEX, CTRL + STRESS, DEX + STRESS, CTRL, DEX, CTRL + STRESS, DEX + STRESS, CTRL, DEX, CTRL + STRESS, DEX + STRESS, CTRL, DEX, CTRL + STRESS, DEX + STRESS, CTRL, DEX, CTRL + STRESS, DEX + STRESS.

Samples from the left side - membrane 2:
CTRL, DEX, CTRL + STRESS, DEX + STRESS, CTRL, DEX, CTRL + STRESS, DEX + STRESS, CTRL, DEX, CTRL + STRESS, DEX + STRESS, CTRL, DEX, CTRL + STRESS, DEX + STRESS, CTRL, DEX, CTRL + STRESS, DEX + STRESS.

The results of the experiment are shown in Table 1.

β-actin as loading control to HDAC1

Frontal cortex (nuclear fraction)

membrane 1 and membrane 2

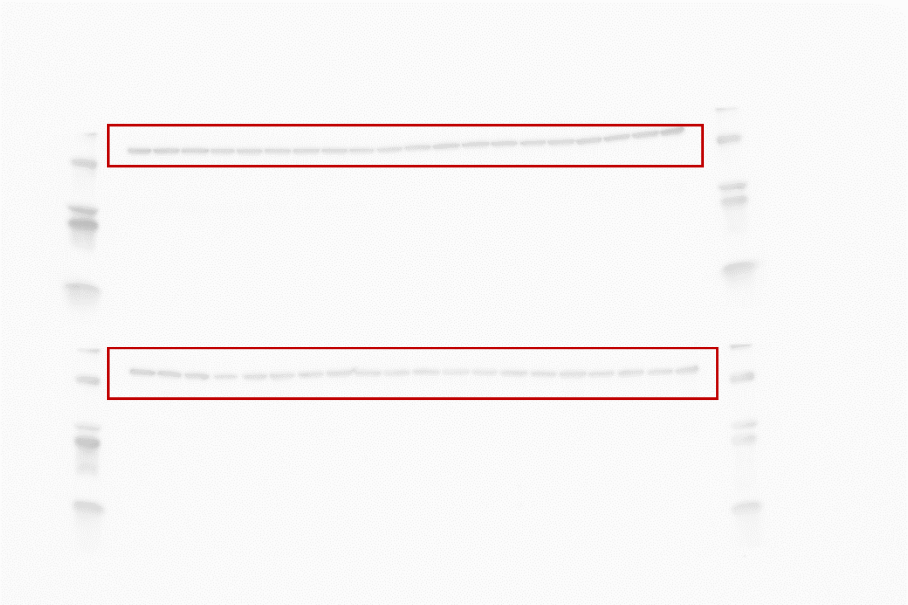

HDAC1

Hippocampus (nuclear fraction)

membrane 1 and membrane 2

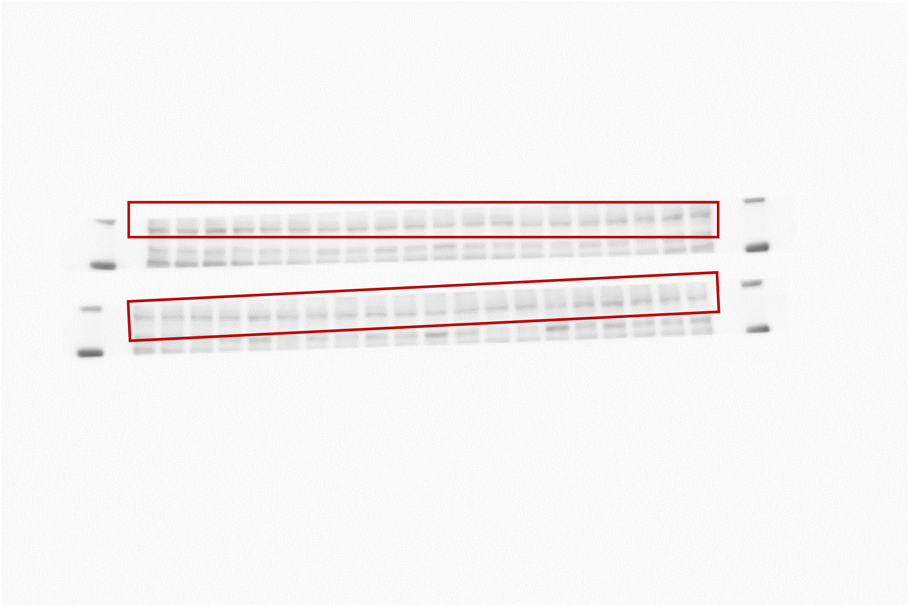

Samples from the left side - membrane 1:
CTRL, DEX, CTRL + STRESS, DEX + STRESS, CTRL, DEX, CTRL + STRESS, DEX + STRESS, CTRL, DEX, CTRL + STRESS, DEX + STRESS, CTRL, DEX, CTRL + STRESS, DEX + STRESS, CTRL, DEX, CTRL + STRESS, DEX + STRESS.

Samples from the left side - membrane 2:
CTRL, DEX, CTRL + STRESS, DEX + STRESS, CTRL, DEX, CTRL + STRESS, DEX + STRESS, CTRL, DEX, CTRL + STRESS, DEX + STRESS, CTRL, DEX, CTRL + STRESS, DEX + STRESS, CTRL, DEX, CTRL + STRESS, DEX + STRESS.

The results of the experiment are shown in Table 1.

β-actin as loading control to HDAC1

Hippocampus (nuclear fraction)

membrane 1 and membrane 2

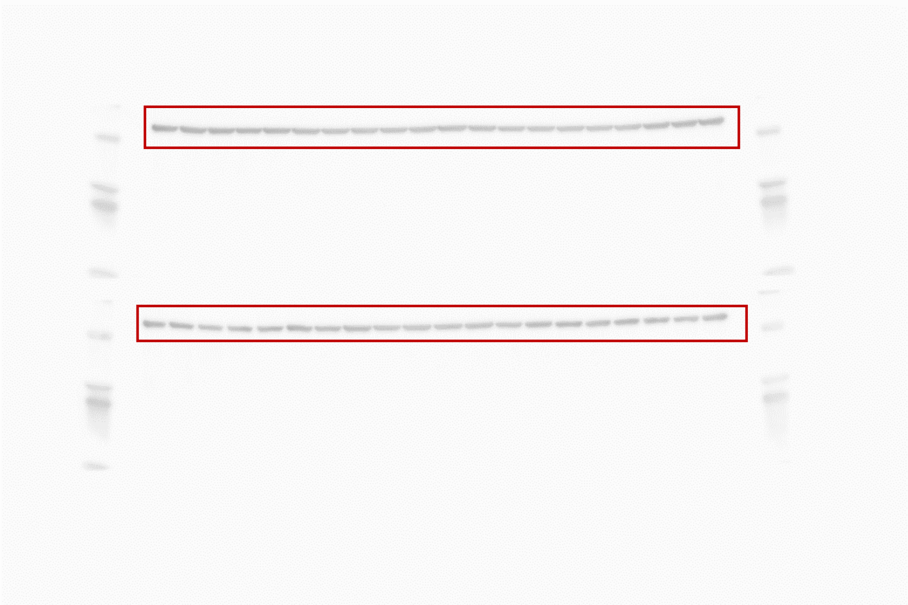

HDAC2

Frontal cortex (nuclear fraction)

membrane 1 and membrane 2

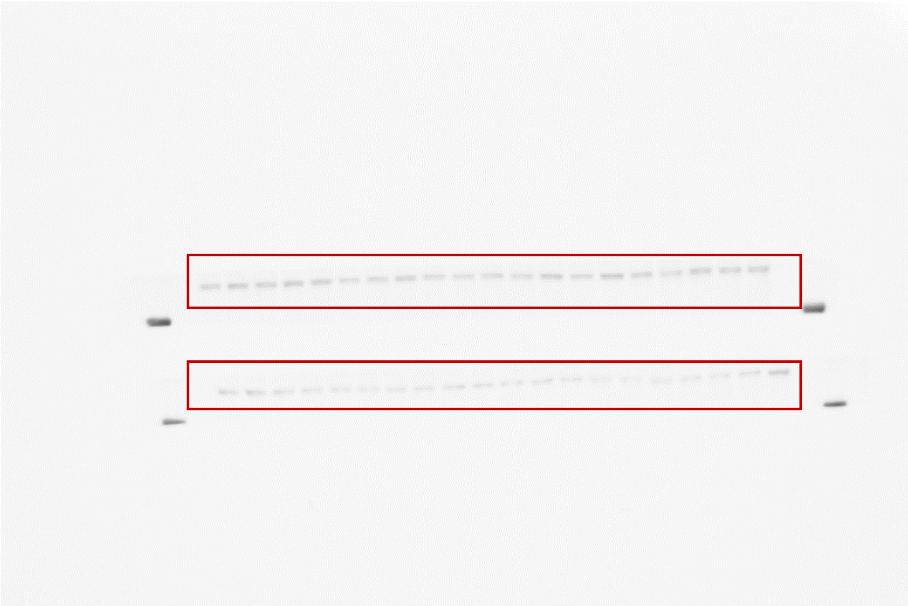

β-actin as loading control to HDAC2

Frontal cortex (nuclear fraction)

membrane 1 and membrane 2

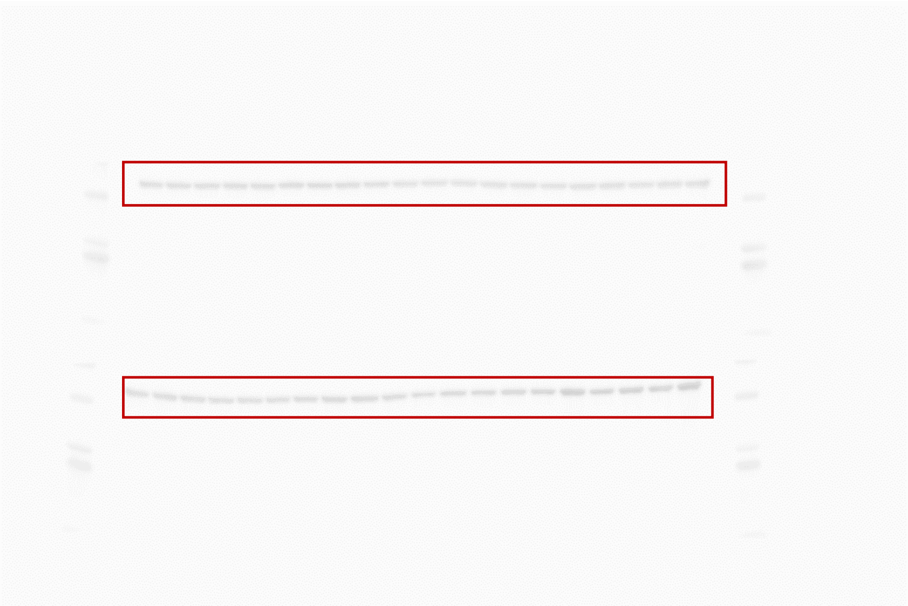

Samples from the left side - membrane 1:
CTRL, DEX, CTRL + STRESS, DEX + STRESS, CTRL, DEX, CTRL + STRESS, DEX + STRESS, CTRL, DEX, CTRL + STRESS, DEX + STRESS, CTRL, DEX, CTRL + STRESS, DEX + STRESS, CTRL, DEX, CTRL + STRESS, DEX + STRESS.

Samples from the left side - membrane 2:
CTRL, DEX, CTRL + STRESS, DEX + STRESS, CTRL, DEX, CTRL + STRESS, DEX + STRESS, CTRL, DEX, CTRL + STRESS, DEX + STRESS, CTRL, DEX, CTRL + STRESS, DEX + STRESS, CTRL, DEX, CTRL + STRESS, DEX + STRESS.

The results of the experiment are shown in Table 1.

HDAC2

Hippocampus (nuclear fraction)

membrane 1 and membrane 2

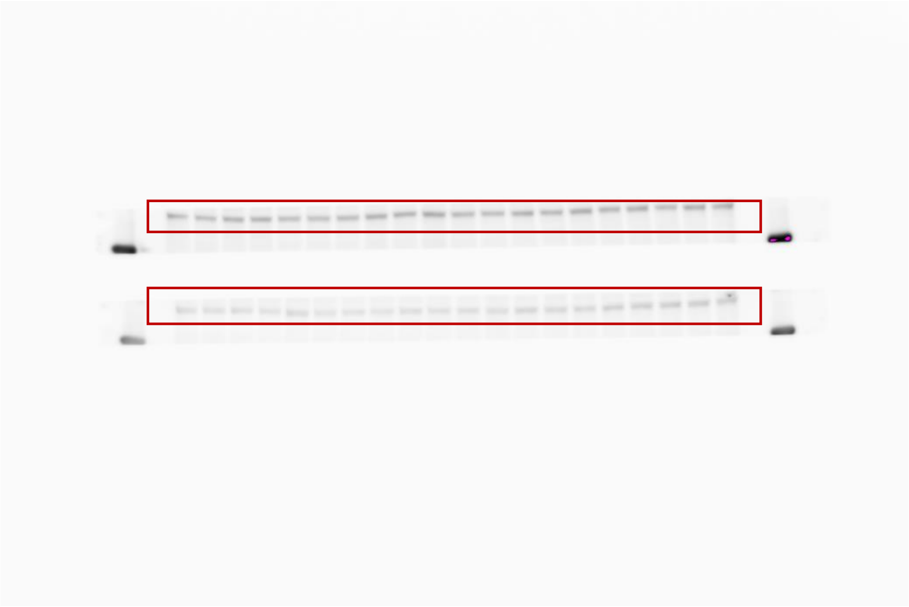

Samples from the left side - membrane 1:
CTRL, DEX, CTRL + STRESS, DEX + STRESS, CTRL, DEX, CTRL + STRESS, DEX + STRESS, CTRL, DEX, CTRL + STRESS, DEX + STRESS, CTRL, DEX, CTRL + STRESS, DEX + STRESS, CTRL, DEX, CTRL + STRESS, DEX + STRESS.

Samples from the left side - membrane 2:
CTRL, DEX, CTRL + STRESS, DEX + STRESS, CTRL, DEX, CTRL + STRESS, DEX + STRESS, CTRL, DEX, CTRL + STRESS, DEX + STRESS, CTRL, DEX, CTRL + STRESS, DEX + STRESS, CTRL, DEX, CTRL + STRESS, DEX + STRESS.

The results of the experiment are shown in Table 1.

β-actin as loading control to HDAC2

Hippocampus (nuclear fraction)

membrane 1 and membrane 2

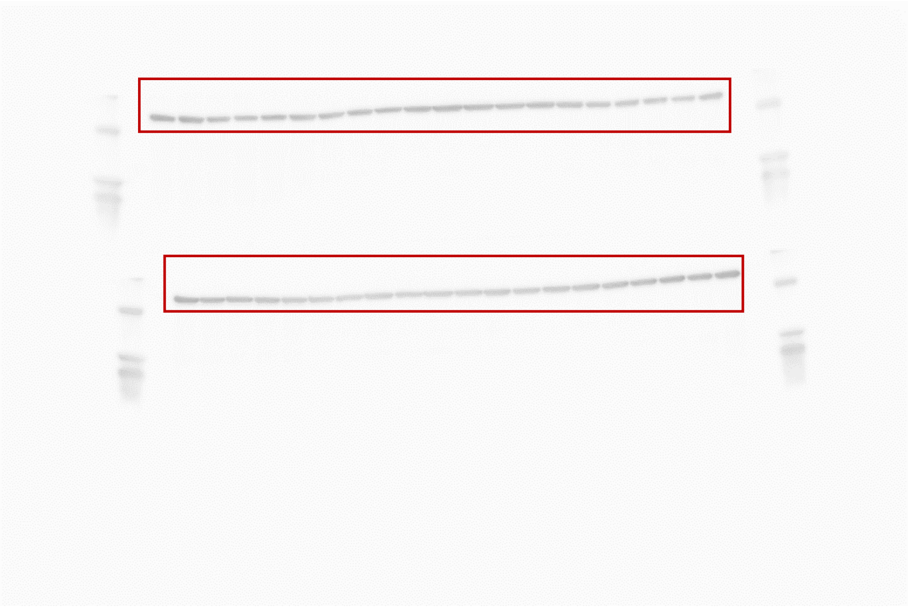

HDAC4

Frontal cortex (nuclear fraction)

membrane 1 and membrane 2

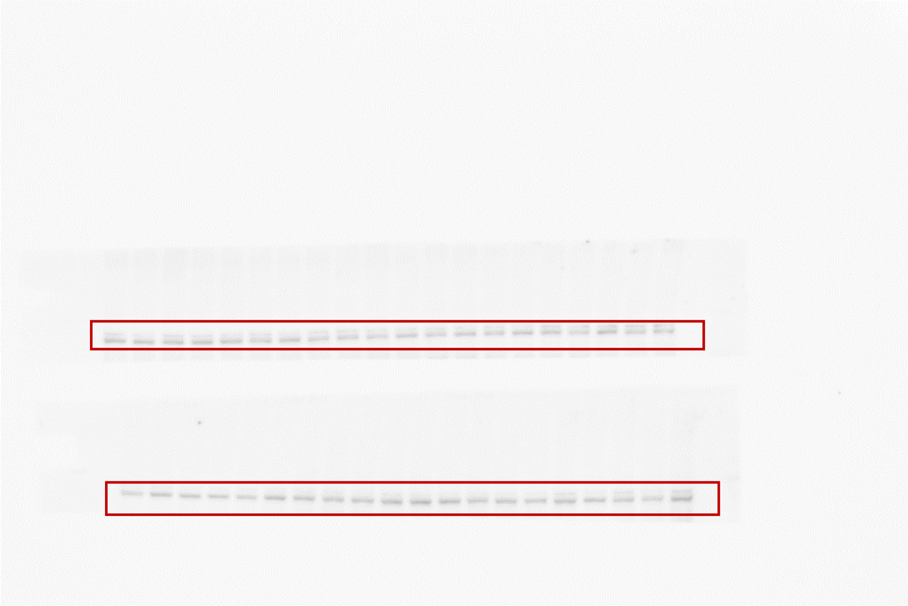

Samples from the left side - membrane 1:
CTRL, DEX, CTRL + STRESS, DEX + STRESS, CTRL, DEX, CTRL + STRESS, DEX + STRESS, CTRL, DEX, CTRL + STRESS, DEX + STRESS, CTRL, DEX, CTRL + STRESS, DEX + STRESS, CTRL, DEX, CTRL + STRESS, DEX + STRESS.

Samples from the left side - membrane 2:
CTRL, DEX, CTRL + STRESS, DEX + STRESS, CTRL, DEX, CTRL + STRESS, DEX + STRESS, CTRL, DEX, CTRL + STRESS, DEX + STRESS, CTRL, DEX, CTRL + STRESS, DEX + STRESS, CTRL, DEX, CTRL + STRESS, DEX + STRESS.

The results of the experiment are shown in Table 1.

β-actin as loading control to HDAC4

Frontal cortex (nuclear fraction)

membrane 1 and membrane 2

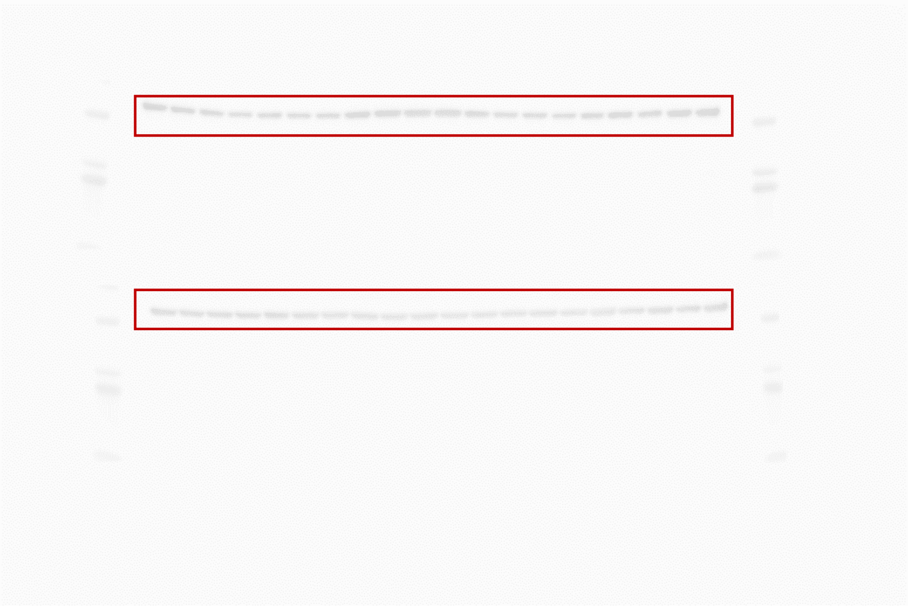

HDAC4

Hippocampus (nuclear fraction)

membrane 1 and membrane 2

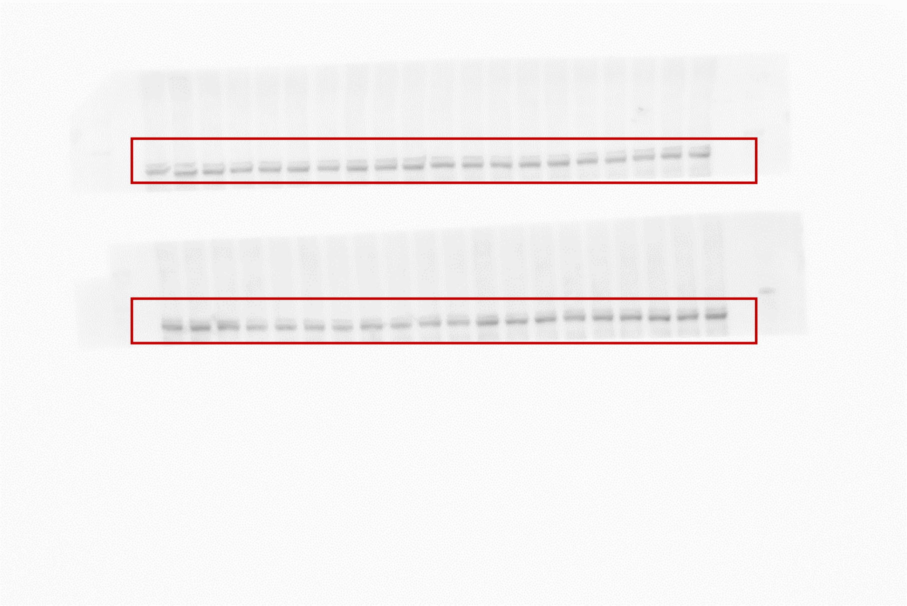

Samples from the left side - membrane 1:
CTRL, DEX, CTRL + STRESS, DEX + STRESS, CTRL, DEX, CTRL + STRESS, DEX + STRESS, CTRL, DEX, CTRL + STRESS, DEX + STRESS, CTRL, DEX, CTRL + STRESS, DEX + STRESS, CTRL, DEX, CTRL + STRESS, DEX + STRESS.

Samples from the left side - membrane 2:
CTRL, DEX, CTRL + STRESS, DEX + STRESS, CTRL, DEX, CTRL + STRESS, DEX + STRESS, CTRL, DEX, CTRL + STRESS, DEX + STRESS, CTRL, DEX, CTRL + STRESS, DEX + STRESS, CTRL, DEX, CTRL + STRESS, DEX + STRESS.

The results of the experiment are shown in Table 1.

β-actin as loading control to HDAC4

Hippocampus (nuclear fraction)

membrane 1 and membrane 2

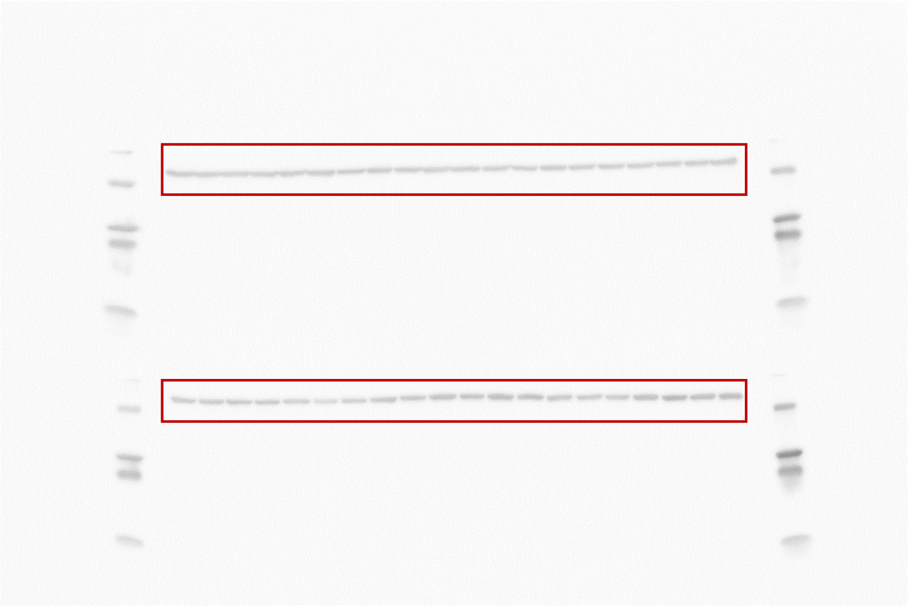

Dnmt1

Frontal cortex (nuclear fraction)

membrane 1 and membrane 2

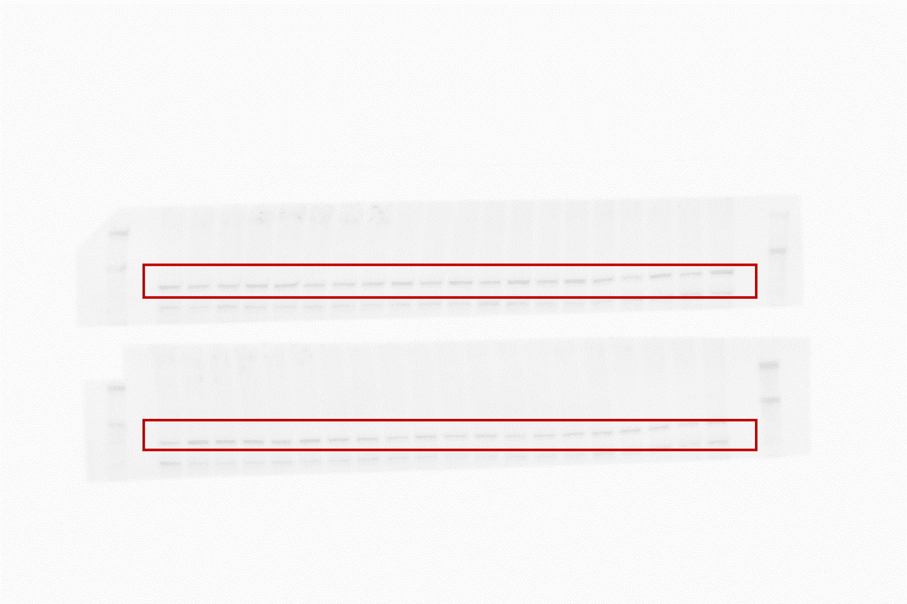

β-actin as loading control to Dnmt1

Frontal cortex (nuclear fraction)

membrane 1 and membrane 2

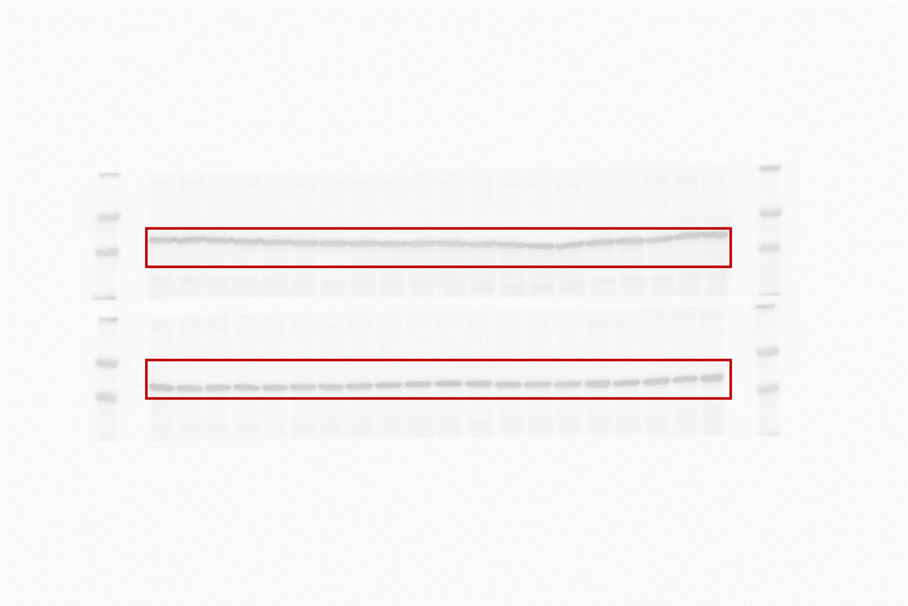

Samples from the left side - membrane 1:
CTRL, DEX, CTRL + STRESS, DEX + STRESS, CTRL, DEX, CTRL + STRESS, DEX + STRESS, CTRL, DEX, CTRL + STRESS, DEX + STRESS, CTRL, DEX, CTRL + STRESS, DEX + STRESS, CTRL, DEX, CTRL + STRESS, DEX + STRESS.

Samples from the left side - membrane 2:
CTRL, DEX, CTRL + STRESS, DEX + STRESS, CTRL, DEX, CTRL + STRESS, DEX + STRESS, CTRL, DEX, CTRL + STRESS, DEX + STRESS, CTRL, DEX, CTRL + STRESS, DEX + STRESS, CTRL, DEX, CTRL + STRESS, DEX + STRESS.

The results of the experiment are shown in Table 1.

Dnmt1

Hippocampus (nuclear fraction)

membrane 1 and membrane 2

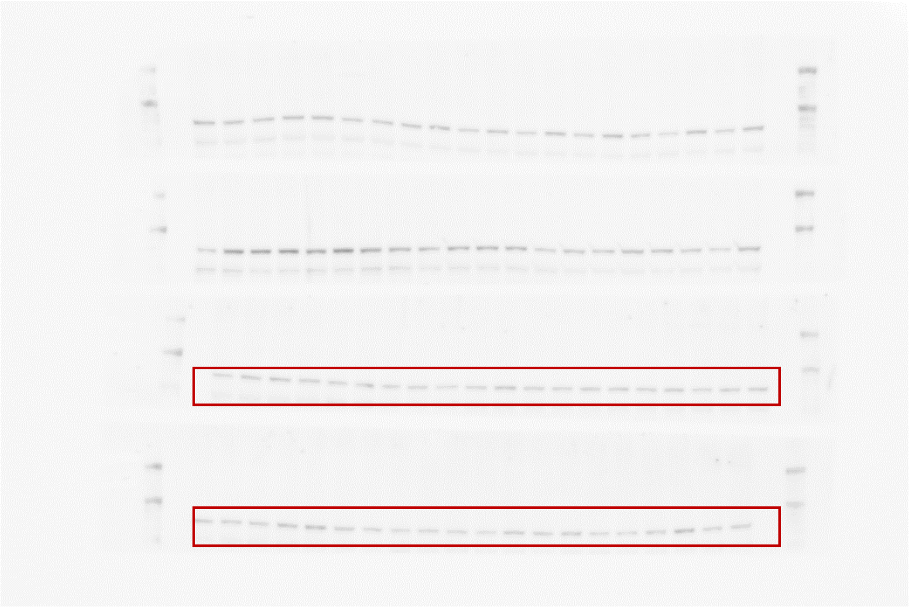

Samples from the left side - membrane 1:
CTRL, DEX, CTRL + STRESS, DEX + STRESS, CTRL, DEX, CTRL + STRESS, DEX + STRESS, CTRL, DEX, CTRL + STRESS, DEX + STRESS, CTRL, DEX, CTRL + STRESS, DEX + STRESS, CTRL, DEX, CTRL + STRESS, DEX + STRESS.

Samples from the left side - membrane 2:
CTRL, DEX, CTRL + STRESS, DEX + STRESS, CTRL, DEX, CTRL + STRESS, DEX + STRESS, CTRL, DEX, CTRL + STRESS, DEX + STRESS, CTRL, DEX, CTRL + STRESS, DEX + STRESS, CTRL, DEX, CTRL + STRESS, DEX + STRESS.

The results of the experiment are shown in Table 1.

β-actin as loading control to Dnmt1

Hippocampus (nuclear fraction)

membrane 1 and membrane 2

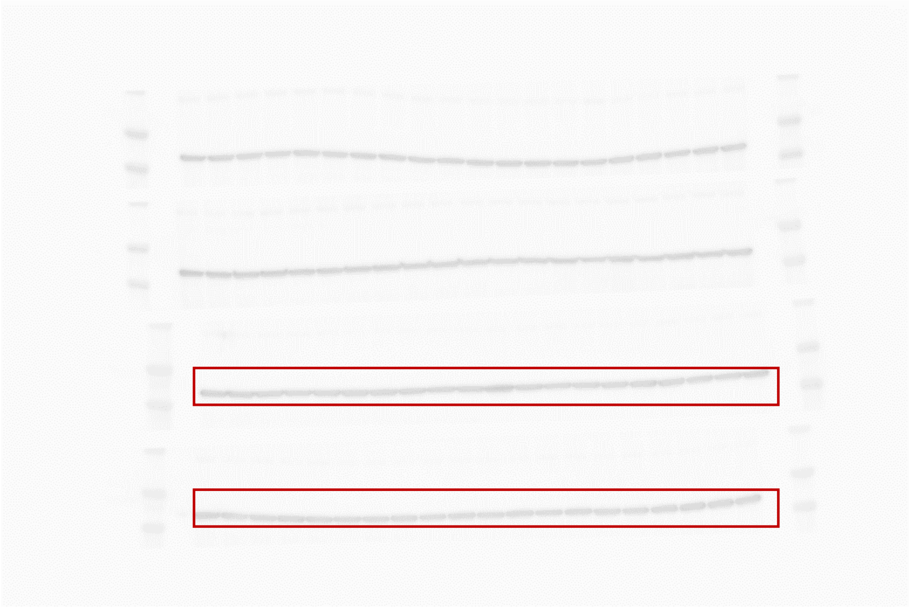

Dnmt3a

Frontal cortex (nuclear fraction)

membrane 1 and membrane 2

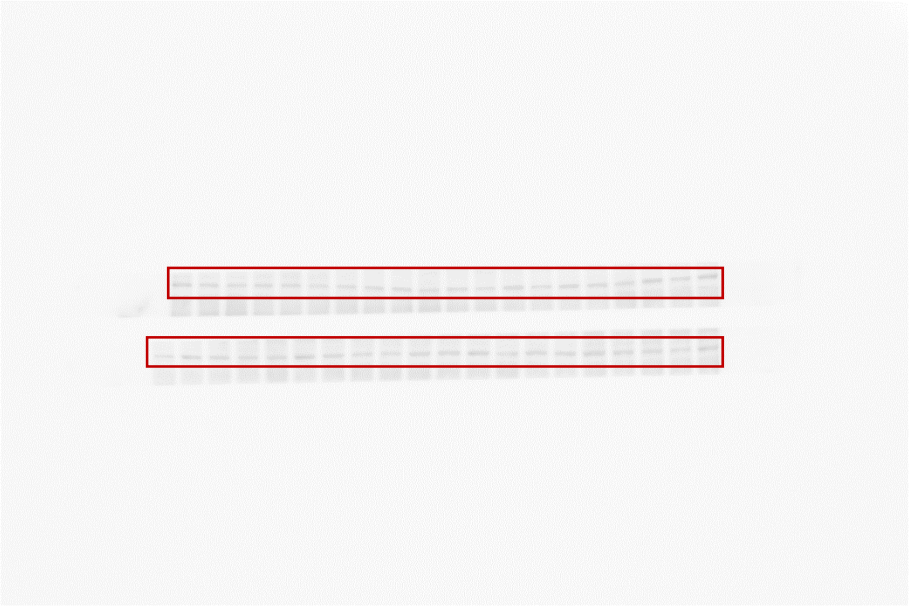

Samples from the left side - membrane 1:
CTRL, DEX, CTRL + STRESS, DEX + STRESS, CTRL, DEX, CTRL + STRESS, DEX + STRESS, CTRL, DEX, CTRL + STRESS, DEX + STRESS, CTRL, DEX, CTRL + STRESS, DEX + STRESS, CTRL, DEX, CTRL + STRESS, DEX + STRESS.

Samples from the left side - membrane 2:
CTRL, DEX, CTRL + STRESS, DEX + STRESS, CTRL, DEX, CTRL + STRESS, DEX + STRESS, CTRL, DEX, CTRL + STRESS, DEX + STRESS, CTRL, DEX, CTRL + STRESS, DEX + STRESS, CTRL, DEX, CTRL + STRESS, DEX + STRESS.

The results of the experiment are shown in Table 1.

β-actin as loading control to Dnmt3a

Frontal cortex (nuclear fraction)

membrane 1 and membrane 2

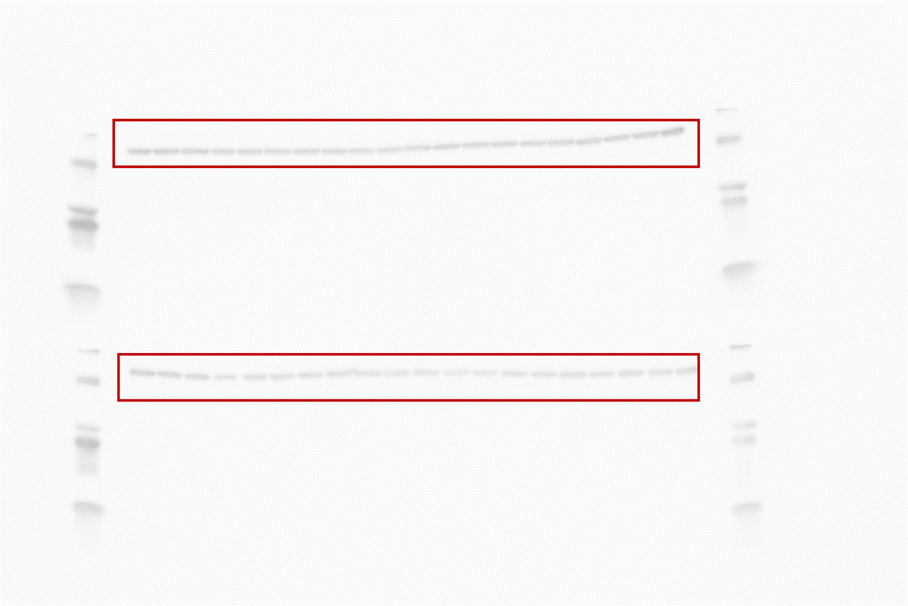

Dnmt3a

Hippocampus (nuclear fraction)

membrane 1 and membrane 2

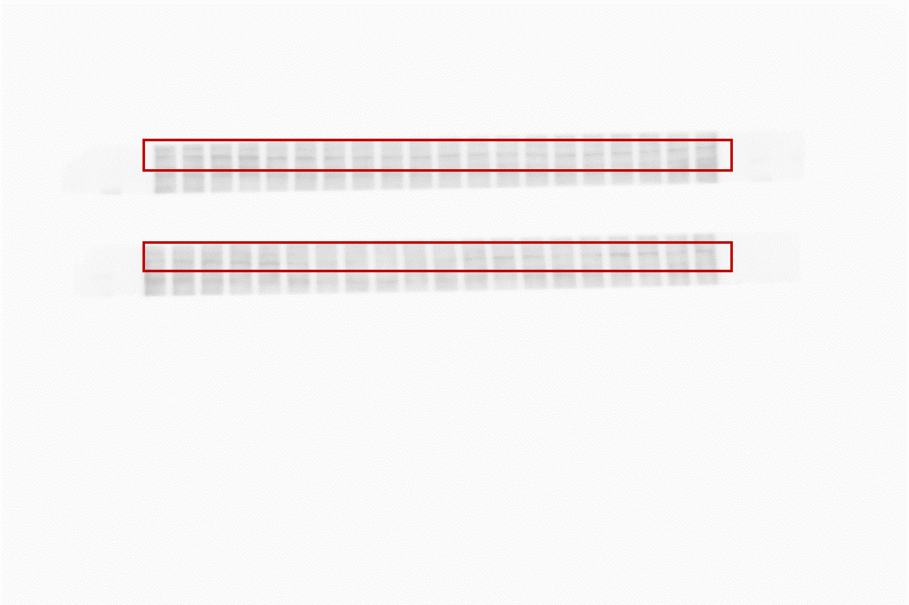

β-actin as loading control to Dnmt3a

Hippocampus (nuclear fraction)

membrane 1 and membrane 2

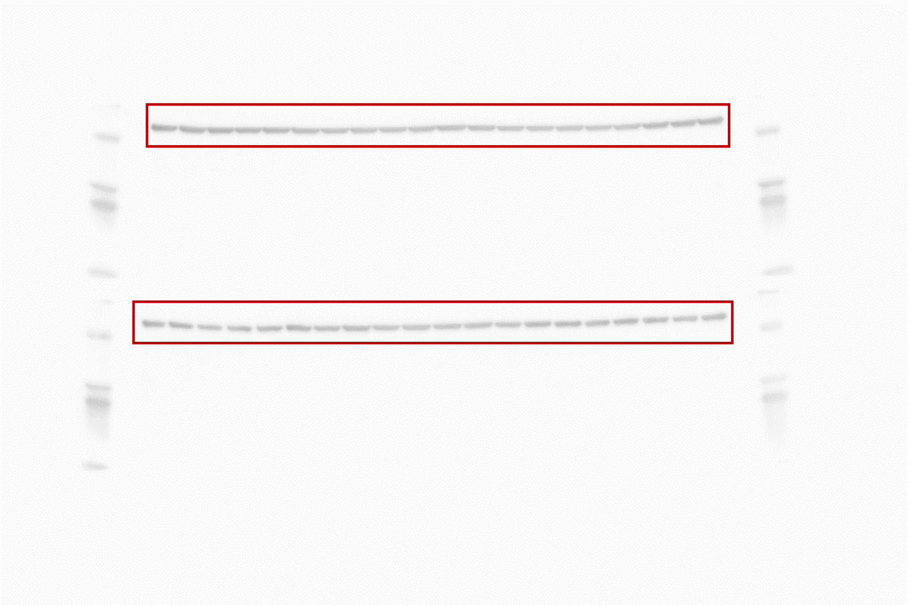

Samples from the left side - membrane 1:
CTRL, DEX, CTRL + STRESS, DEX + STRESS, CTRL, DEX, CTRL + STRESS, DEX + STRESS, CTRL, DEX, CTRL + STRESS, DEX + STRESS, CTRL, DEX, CTRL + STRESS, DEX + STRESS, CTRL, DEX, CTRL + STRESS, DEX + STRESS.

Samples from the left side - membrane 2:
CTRL, DEX, CTRL + STRESS, DEX + STRESS, CTRL, DEX, CTRL + STRESS, DEX + STRESS, CTRL, DEX, CTRL + STRESS, DEX + STRESS, CTRL, DEX, CTRL + STRESS, DEX + STRESS, CTRL, DEX, CTRL + STRESS, DEX + STRESS.

The results of the experiment are shown in Table 1.

Dnmt3b

Frontal cortex (nuclear fraction)

membrane 1 and membrane 2

β-actin as loading control to Dnmt3b

Frontal cortex (nuclear fraction)

membrane 1 and membrane 2

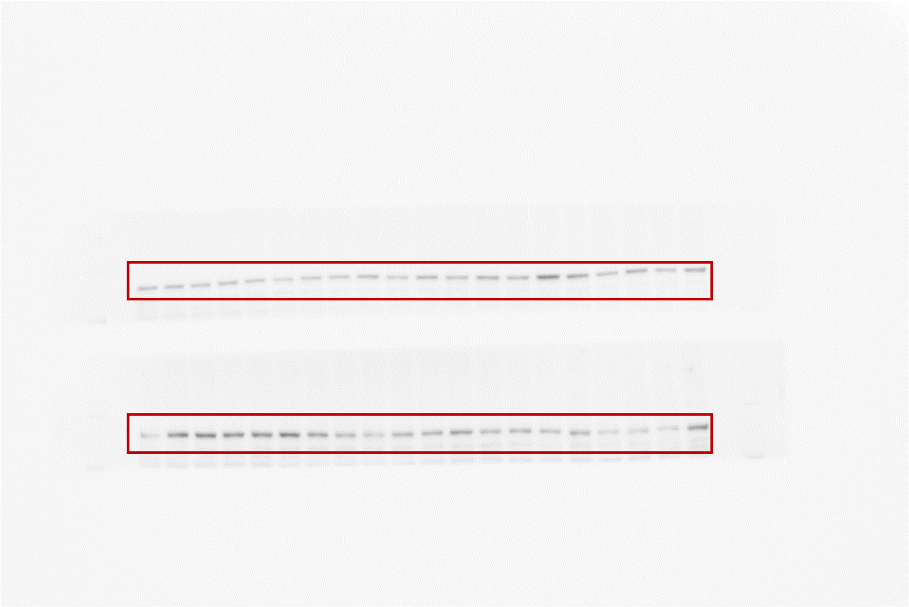

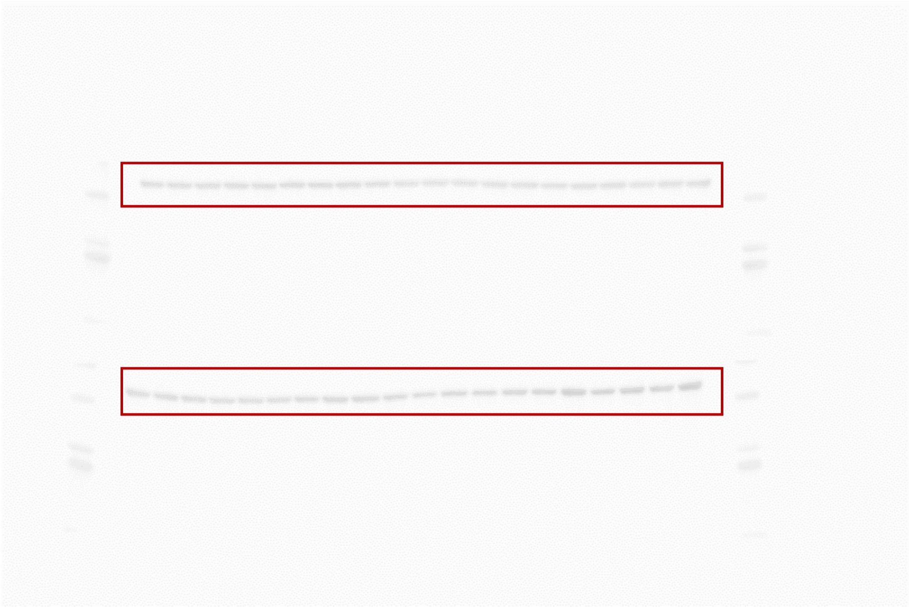

Samples from the left side - membrane 1:
CTRL, DEX, CTRL + STRESS, DEX + STRESS, CTRL, DEX, CTRL + STRESS, DEX + STRESS, CTRL, DEX, CTRL + STRESS, DEX + STRESS, CTRL, DEX, CTRL + STRESS, DEX + STRESS, CTRL, DEX, CTRL + STRESS, DEX + STRESS.

Samples from the left side - membrane 2:
CTRL, DEX, CTRL + STRESS, DEX + STRESS, CTRL, DEX, CTRL + STRESS, DEX + STRESS, CTRL, DEX, CTRL + STRESS, DEX + STRESS, CTRL, DEX, CTRL + STRESS, DEX + STRESS, CTRL, DEX, CTRL + STRESS, DEX + STRESS.

The results of the experiment are shown in Table 1.

Dnmt3b

Hippocampus (nuclear fraction)

membrane 1 and membrane 2

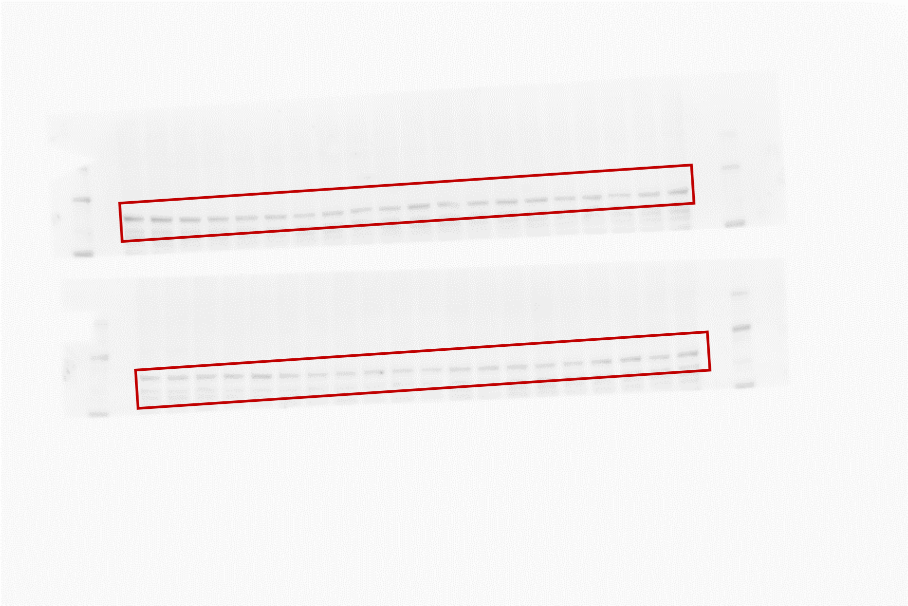

Samples from the left side - membrane 1:
CTRL, DEX, CTRL + STRESS, DEX + STRESS, CTRL, DEX, CTRL + STRESS, DEX + STRESS, CTRL, DEX, CTRL + STRESS, DEX + STRESS, CTRL, DEX, CTRL + STRESS, DEX + STRESS, CTRL, DEX, CTRL + STRESS, DEX + STRESS.

Samples from the left side - membrane 2:
CTRL, DEX, CTRL + STRESS, DEX + STRESS, CTRL, DEX, CTRL + STRESS, DEX + STRESS, CTRL, DEX, CTRL + STRESS, DEX + STRESS, CTRL, DEX, CTRL + STRESS, DEX + STRESS, CTRL, DEX, CTRL + STRESS, DEX + STRESS.

The results of the experiment are shown in Table 1.

β-actin as loading control to Dnmt3b

Hippocampus (nuclear fraction)

membrane 1 and membrane 2

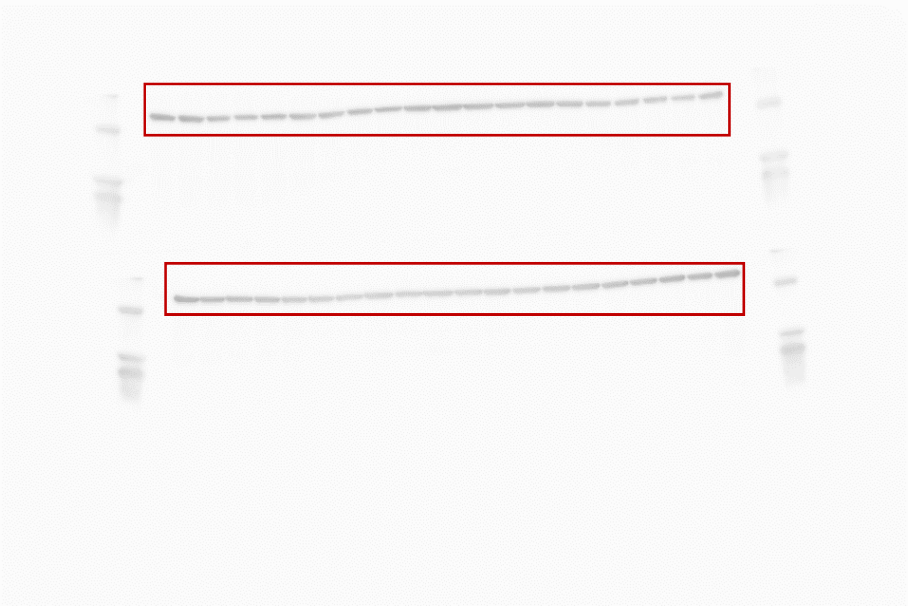

Not all WB samples (presented on pages 2-33) were analyzed (poor quality or outliers were not analyzed).
